# A Review on Surface-Enhanced Raman Scattering

**DOI:** 10.3390/bios9020057

**Published:** 2019-04-17

**Authors:** Roberto Pilot, Raffaella Signorini, Christian Durante, Laura Orian, Manjari Bhamidipati, Laura Fabris

**Affiliations:** 1 Department of Chemical Sciences, University of Padova, 35131 Padova, Italy; raffaella.signorini@unipd.it (R.S.); christian.durante@unipd.it (C.D.); laura.orian@unipd.it (L.O.); 2 Consorzio INSTM, via G. Giusti 9, 50121 Firenze, Italy; 3 Department of Biomedical Engineering, Rutgers University, 599 Taylor Road, Piscataway, NJ 08854, USA; manjari.b@rutgers.edu; 4 Department of Materials Science and Engineering, Rutgers University, 607 Taylor Road, Piscataway, NJ 08854, USA; lfabris@soe.rutgers.edu

**Keywords:** SERS, Raman, surface enhanced, electromagnetic enhancement, chemical enhancement, substrates, underpotential deposition, excitation wavelength, biomedical applications, enhancement factor

## Abstract

Surface-enhanced Raman scattering (SERS) has become a powerful tool in chemical, material and life sciences, owing to its intrinsic features (i.e., fingerprint recognition capabilities and high sensitivity) and to the technological advancements that have lowered the cost of the instruments and improved their sensitivity and user-friendliness. We provide an overview of the most significant aspects of SERS. First, the phenomena at the basis of the SERS amplification are described. Then, the measurement of the enhancement and the key factors that determine it (the materials, the hot spots, and the analyte-surface distance) are discussed. A section is dedicated to the analysis of the relevant factors for the choice of the excitation wavelength in a SERS experiment. Several types of substrates and fabrication methods are illustrated, along with some examples of the coupling of SERS with separation and capturing techniques. Finally, a representative selection of applications in the biomedical field, with direct and indirect protocols, is provided. We intentionally avoided using a highly technical language and, whenever possible, intuitive explanations of the involved phenomena are provided, in order to make this review suitable to scientists with different degrees of specialization in this field.

## Index

**1. Introduction**
**2. Origin of the SERS Enhancement**
   *2.1.* *Electromagnetic Enhancement*
    2.1.1. Local Field Enhancement
    2.1.2. Re-Radiation Enhancement
    2.1.3. Expression for the SERS Enhancement in the ${\left|E\right|}^{4}$ Approximation
   *2.2.* *Chemical Enhancement*
    2.2.1. Computational Approach: Resonant and Non-Resonant Chemical Effect
    2.2.2. Modelling Approach: Resonant Chemical Effect
**3. Main Factors Affecting the SERS Enhancement and Its Experimental Determination**
   *3.1.* *Materials for SERS*
    3.1.1. Metallic Materials
    3.1.2. Non-Metallic Materials for SERS and Specific Mechanisms Involved in the Enhancement
   *3.2.* *The Role of Hot Spots*
    3.2.1. Anatomy of a Hot Spot: Spatial Distribution of the Local Field
   *3.3.* Distance Dependence of the Electromagnetic Enhancement
   *3.4.* *Experimental Determination of the SERS Enhancement*
**4. Factors Affecting the Choice of the Excitation Wavelength in a SERS Experiment**
   *4.1.* *SERS Enhancement*
   *4.2.* *The Analyte Cross-Section*
   *4.3.* *Fluorescence from the Analyte or Contaminants*
   *4.4.* *Spectral Sensitivity of the Raman Instrument*
   *4.5.* *Summary*
**5. Fabrication of SERS Substrates**
   *5.1.* *Desired Features of SERS Substrates for Applications*
   *5.2.* *Direct vs. Indirect Detection*
   *5.3.* *Substrates for Direct Detection*
    5.3.1. Aggregated Nanoparticles in Solution (Unstructured Nanoparticles)
    5.3.2. Nanoparticles Assembled on a Surface (Structured Nanoparticles)
    5.3.3. Ordered Arrays of Nanoparticles (Structured Surfaces)
   *5.4.* *SERS Labels for Indirect Detection*
   *5.5.* *Commercial Substrates*
   *5.6.* *Some Analytical Aspects of SERS Substrates: Separation and Capturing Techniques*
    5.6.1. Separation Techniques
    5.6.2. Capturing Techniques
**6. Applications in the Biomedical Field**
   *6.1.* *Direct Protocol*
    6.1.1. DNA Detection
    6.1.2. Analysis of Cellular Functions and Components in the Cell Microenvironment
    6.1.3. Protein Detection
    6.1.4. Viruses and Bacteria
   *6.2.* *Indirect Protocol*
    6.2.1. DNA Detection
    6.2.2. Analysis of Cellular Functions and Components in the Cell Microenvironment
    6.2.3. Protein Detection
**7. Conclusions and Outlook**
**References**

## 1. Introduction

Surface-enhanced Raman scattering (SERS) was first observed in 1974 by Fleischmann et al. [1], who reported an unexpectedly large Raman signal from pyridine adsorbed on a roughened silver electrode. Before long, Jeanmaire and van Duyne [2] and Albrecht and Creighton [3] confirmed Fleishman’s findings and hypothesized that this phenomenon was originated by strong electrochemical electric fields at the metal surface (Jeanmaire) or by the formation of a molecule–metal complex (Albrecht); lately, Moskovits [4,5] proposed that the large signal was originated by the optical excitation of collective oscillations of the electrons in the metallic nanosized features at the surface. Studies in the following years confirmed that the origin of SERS enhancement is two-fold and is related to the electromagnetic [6,7,8] and to the chemical effect [6,8,9,10,11]: a reasonable maximum value for the total enhancement is around 10 orders of magnitude [12]. SERS; therefore, combines the intrinsic advantages of Raman ((a) recognition capabilities, owing to the vibrational fingerprints of molecules; (b) non-destructive analysis; (c) minimum preparation of the sample required; (d) possibility of carrying out measurements in biological fluids, since the water spectrum is rather weak; (e) simultaneous detection of different analytes (multiplexing); (f) possibility of carrying out on-site analysis with portable instruments) [13] with high sensitivity that, in some cases, can even allow single molecule detection [14,15,16,17].

It is worth mentioning that also the technological developments of the instrumentation provided, in the last thirty years, important contributions to the field of Raman/SERS spectroscopies [18]; for example, one can mention the introduction of charged coupled devices (CCDs) that, owing to their multichannel configuration, strongly improved the quality (i.e., the signal to noise ratio, SNR) of the collected spectra, the invention of holographic notch filters that replaced (for many applications) the bulky and pricey triple spectrographs in the rejection of the Rayleigh scattering, and the introduction of compact and cheap solid state lasers for excitation, available at several emission wavelengths. A technical description of the above mentioned components can be found in several books [19,20]. Handheld or portable Raman instruments became available from the early 2000s. Interestingly, as reported by Carron et al. [21], the miniaturization and the lowering of the costs of Raman instruments strongly benefited from the improvements of two key components, the laser sources, and the CCD detectors, driven by the commercialization of consumer electronic products such as compact disk players and digital cameras. 

The intrinsic features of SERS, along with the instrumental advancements, have triggered the application of this spectroscopic tool in many fields and are transforming it from a technique accessible to a small number of specialized users, like it was in the past, to a more widely available analytical technique.

Nowadays, scientists are working on several aspects of SERS, for example, fundamental aspects related to the electromagnetic [7,12,22,23] or the chemical [9,10,24,25] enhancement mechanisms, single molecule detection [14,15,16,17], structure-property investigations aimed to investigate how the structure of a SERS substrate influences its optical response [26,27], tip enhanced Raman scattering (TERS) [28,29,30,31,32], and ultrafast SERS studies of molecular dynamics at the interface with metallic surfaces [33,34]. Another important field is the development of substrates with optimal characteristics for SERS; fabrication strategies have been reviewed in several papers [35,36,37,38,39] and comprise, for example, wet chemical protocols [40], the assembly of nanoparticles on different types of surfaces [41,42], and the fabrication of ordered arrays of nanoparticles [43,44,45,46]. Analytical aspects of SERS, like the coupling with separation techniques (gas, liquid, thin layer chromatography, etc.) have been recently reviewed [47], as well as the issues involved in the quantitative determination of analytes [48,49,50,51,52,53]. Concerning applications, SERS has been used for the detection of food additives or contaminants [54,55,56,57], explosives and warfare agents [58], biological species [59,60,61,62,63,64,65,66,67,68,69,70,71,72,73,74,75,76], in forensic science [77], and to monitor reactions catalyzed by metallic surfaces [78] or nanoparticles [79]. General overviews on SERS can be found in books [6,8,80,81,82,83,84], Faraday discussions [85,86,87,88,89], special issues [90,91,92,93], and reviews [7,9,36,76,94,95,96,97,98]. Finally, it is worth mentioning that the plasmonic amplification of the optical response has been exploited to enhance also coherent anti-Stokes Raman scattering (CARS) [99,100], stimulated Raman scattering (SRS) [101,102], hyper Raman scattering (HRS) [75,103,104,105], fluorescence [106,107,108,109], and infrared absorption [8,110].

This review is aimed to provide an overview of the key aspects of SERS: we have avoided using a highly technical language and, whenever possible, we have provided intuitive explanations of the phenomena involved, in order to make this paper suitable to scientists with different degrees of specialization in this field. An extensive bibliography is present in each section, so that the reader can go into detail in the specific subjects of interest and hopefully find useful information for his/her research work.

## 2. Origin of the SERS Enhancement

Raman scattering is the inelastic scattering of photons that can occur when they interact with matter. As a consequence of this interaction, photons can lose energy in favor of a molecule that gets promoted from the ground state to its first excited vibrational state (Stokes Raman scattering) or, conversely, gain energy from a molecule that undergoes the opposite process (anti-Stokes Raman scattering); therefore, inelastically scattered photons contain information on the vibrational modes of the materials they interact with [19,111]. The cross-section ratio between anti-Stokes and Stokes bands of the k-th vibrational mode ($\rho_{k}^{aS/S}=\frac{\sigma_{k}^{aS}}{\sigma_{k}^{S}}$) is related to the population ratio between the ground and the first vibrationally excited state [111,112,113]: $\rho_{k}^{aS/S}=\frac{\sigma_{k}^{aS}}{\sigma_{k}^{S}}={\left(\frac{{\tilde{\nu }}_{0}+{\tilde{\nu }}_{k}}{{\tilde{\nu }}_{0}-{\tilde{\nu }}_{k}}\right)}^{3}e^{-\;\frac{hc{\tilde{\nu }}_{k}}{k_{B}T}}$, where h (in [J*s]) is the Plank constant, c (in [cm/s]) is the speed of light in vacuum, $k_{B}$ (in [J/K]) is the Boltzmann constant, T (in [K]) is the temperature, ${\tilde{\nu }}_{0}$ (in [cm^−1^]) the excitation laser wavenumber, and ${\tilde{\nu }}_{k}$ (in [cm^−1^]) is the Raman shift of the k-th vibrational mode. At room temperature, for ${\tilde{\nu }}_{0}\sim 20,000$ cm^−1^ (514.5 nm), $\rho_{k}^{aS/S}$ amounts to about 0.01 for ${\tilde{\nu }}_{k}=$ 1000 cm^−1^ and to 0.1 for ${\tilde{\nu }}_{k}=$ 500 cm^−1^. Notably, the expression for $\rho_{k}^{aS/S}$ is valid when a photon counter detector is used to collect the Raman spectra, that is always the case with today’s instruments; if energy based detectors were used, the term $\left(\frac{{\tilde{\nu }}_{0}+{\tilde{\nu }}_{k}}{{\tilde{\nu }}_{0}-{\tilde{\nu }}_{k}}\right)$ would be elevated to the fourth power, rather than to the third power [112].

Raman spectra normally report Stokes bands, due to their remarkably stronger intensity compared to the anti-Stokes bands. The Raman signal generated by a sample (from now on intended as Stokes-Raman) can be written as:

(1) $$P_{Raman}=KN\sigma_{k}I$$

where $P_{Raman}$ (in [photons/s]) is the Raman power measured by the detector; K accounts for the fraction of photons that, once emitted from the molecules, are collected and converted into electrons by the detector (it includes several instrumental parameters); N is the number of illuminated molecules; $\sigma_{k}$ (in [cm^2^/molecule]) is the Raman cross-section of the *k*-th mode integrated over the bandwidth and over all emission directions; and I (in [photons/(cm^2^·s)]) is the laser intensity impinging on the sample [81].

Raman is an intrinsically very weak phenomenon, approximately six to 10 orders of magnitude less efficient than fluorescence [6]. However, the Raman scattering generated by molecules can be strongly amplified by placing them near the surface of suitably nanostructured substrates; in this case, we talk about surface-enhanced Raman scattering (SERS), rather than simple Raman, to emphasize the amplification effect brought about by the presence of the substrate. The Raman and SERS scattered powers ($P_{Raman}$ and $P_{SERS}$, respectively) are formally related by the following equation:

(2) $$P_{SERS}=G_{SERS}P_{Raman}=G_{SERS}^{Em}G_{SERS}^{Chem}P_{Raman}$$

$G_{SERS}$ is called (total) SERS enhancement factor and accounts for the amplification induced by the substrate. The total SERS enhancement comprises two multiplicative contributions, the electromagnetic ($G_{SERS}^{Em}$) and the chemical one ($G_{SERS}^{Chem}$), whose features are summarized in the following and will be described in detail in the Section 2.1 and Section 2.2.

Electromagnetic Enhancement ($G_{SERS}^{Em}$)

- It originates from the localization of light at the surface of the substrate.
- It is a feature typical of the substrate and it is independent of the type of molecule.
- It is the strongest contribution to the SERS enhancement and it can reach very high values, around 10^10^ (see Table 1).
- In order to be effective, it requires the molecule to be placed not too far from the substrate (about 1 to 10 nm away from the surface). It is considered a long-range effect (compared to the length of a chemical bond).

Chemical Enhancement ($G_{SERS}^{Chem}$)

- The chemical enhancement arises from a modification of the polarizability of a molecule (and hence of the Raman cross-sections of its vibrational modes), as a consequence of its physico-chemical interaction with the substrate.
- It depends on the type of molecule.
- The contribution of the chemical enhancement is normally considered much smaller than the electromagnetic one and, depending on the specific mechanism involved, its magnitude may reach 10^2^–10^4^ (see Table 1).
- It requires contact or a very small separation (a few Angstroms) [114] between the molecule and the substrate. It is considered a short-range effect.

The separation between chemical and electromagnetic enhancement may be not so clear cut as presented before, for example, the relative orientation of the molecule with respect to the local field may cause the Raman bands to be enhanced differently depending on the symmetry of their Raman polarizability tensor. This effect is; therefore, electromagnetic in origin but also depends on the type of molecule and its orientation on the surface. A discussion on orientation effects can be found in the book by Le Ru et al. [6].

Typically, chemical and electromagnetic enhancements are treated as separate phenomena and in this review we shall follow this line. Nevertheless, scientists are attempting to create a unified theory of SERS and the current status is described in the paper by Ding et al. [7].

### 2.1. Electromagnetic Enhancement

Let us suppose that a molecule is placed at the surface of a metallic substrate that supports the excitation of surface plasmons when illuminated with laser light (here we specifically refer to metals as enhancing materials, because they are the most commonly used; however, it will be shown in Section 3.1.2 that also non-metallic materials may exhibit SERS properties). The electromagnetic enhancement possesses two distinct contributions:

- The local field (or near field) enhancement. The excitation of surface plasmons induces a strong spatial localization and hence amplification of the laser light in small spatial regions, called hot spots. Therefore, the electromagnetic field experienced by the molecules residing in hot spots is much stronger than the field they would experience without the metallic substrate.
- The re-radiation enhancement. The presence of the metallic structure nearby the molecule modifies also the efficiency with which the molecule radiates Raman power; this occurs because the power radiated by a dipole (i.e., the molecule oscillating at the Raman frequency) depends on the environment in which it is embedded.

**Figure 1:**
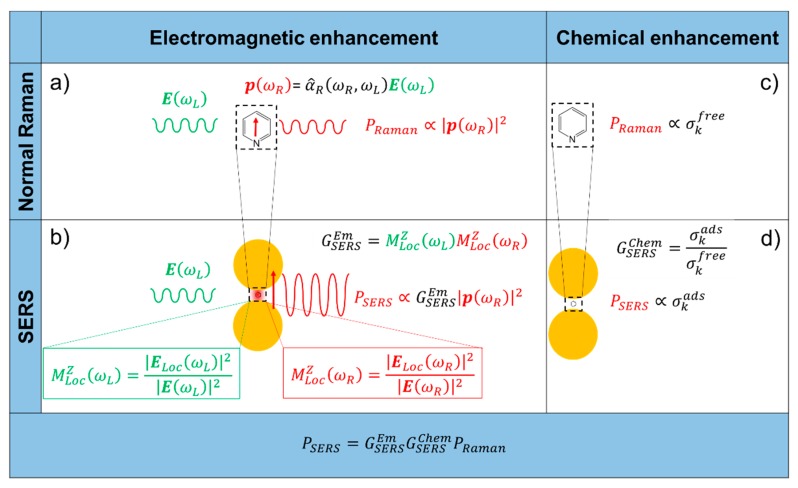
Electromagnetic enhancement. (**a**) Normal Raman. A laser radiation, with electric field $E\left(\omega_{L}\right)$ oscillating at (angular) frequency $\omega_{L}$ impinges on a molecule, characterized by a Raman polarizability tensor ${\hat{\alpha }}_{R}\left(\omega_{R},\omega_{L}\right)$. The laser induces a dipole oscillating at the Raman frequency (vertical red arrow, $p\left(\omega_{R}\right)$); the Raman power radiated by this dipole is proportional to the square modulus of the dipole itself. (**b**) Surface enhanced Raman scattering (SERS) electromagnetic enhancement. When the molecule is placed near a plasmonic substrate, the electric field experienced by the molecule is $E_{Loc}\left(\omega_{L}\right)$, normally much stronger than the input laser $E\left(\omega_{L}\right)$; this local field enhancement is quantified by $M_{Loc}^{Z}\left(\omega_{L}\right)$. Moreover, the presence of the plasmonic substrate also enhances the efficiency with which the dipole emits Raman radiation; this re-radiation enhancement is quantified by $M_{Loc}^{Z}\left(\omega_{R}\right)$. The total electromagnetic enhancement factor, within the ${\left|E\right|}^{4}$ approximation, is defined as: $G_{SERS}^{Em}=M_{Loc}^{Z}\left(\omega_{L}\right)M_{Loc}^{Z}\left(\omega_{R}\right)$. Chemical enhancement. (**c**) Normal Raman. The vibrational modes of a molecule in free space are characterized by the cross-section(s) $\sigma_{k}^{free}$; (**d**) SERS chemical enhancement. The interaction with the plasmonic substrate modifies the structure of the molecule and consequently also the cross-section(s) of its modes ($\sigma_{k}^{ads}$). The chemical enhancement is quantified as $G_{SERS}^{Chem}=\frac{\sigma_{k}^{ads}}{\sigma_{k}^{free}}$.

The electromagnetic and chemical mechanisms are illustrated in Figure 1.

#### 2.1.1. Local Field Enhancement

The easiest way to figure out and quantify the local field enhancement is to resort to the classical view of the Raman scattering, in which the external electric field ($E\left(\omega_{L}\right)$), oscillating at the laser (angular) frequency $\omega_{L}$, induces in the molecule a dipole ($p\left(\omega_{R}\right)$) oscillating at the Raman (angular) frequency $\omega_{R}$ [6,81,111]:

(3) $$p\left(\omega_{R}\right)={\hat{\alpha }}_{R}\left(\omega_{R},\omega_{L}\right)E\left(\omega_{L}\right)$$

${\hat{\alpha }}_{R}\left(\omega_{R},\omega_{L}\right)$ is the Raman polarizability tensor of the molecule. The radiated power ($P_{Raman}$) is proportional to the square modulus of the dipole [6]:

(4) $$P_{Raman}=\frac{\omega_{R}^{4}}{12\pi \varepsilon_{0}c^{3}}{\left|p\left(\omega_{R}\right)\right|}^{2}=\frac{\omega_{R}^{4}}{12\pi \varepsilon_{0}c^{3}}{\left|{\hat{\alpha }}_{R}\left(\omega_{R},\omega_{L}\right)E\left(\omega_{L}\right)\right|}^{2}$$

$\varepsilon_{0}$ is the dielectric constant in vacuum and c is the speed of light in vacuum. In this case, $P_{Raman}$ is derived from the classical physics and is expressed in [W] rather than in [photons/s] as it is in Equation (1), but we have used the same symbol for simplicity (the two quantities are related by the expression $P_{Raman}$ [W] = $\hbar \omega \;P_{Raman}$ [photons/s]); the angular frequency ω is expressed in [radians/s] and is related to the wavenumber, normally used in vibrational spectroscopy, by the relation: $\omega =2\pi c\tilde{\nu }$. The presence of a metallic substrate nearby the molecule can be accounted for by considering that the molecule experiences a local electric field ($E_{Loc}\left(\omega_{L}\right)$) stronger than the input one; Equation (4) clearly suggests that the higher the electric field, the higher the radiated power. The local field enhancement can be defined as:

(5) $$M_{Loc}^{Z}\left(\omega_{L}\right)=\frac{{\left|E_{Loc}\left(\omega_{L}\right)\right|}^{2}}{{\left|E\left(\omega_{L}\right)\right|}^{2}}$$

The superscript Z indicates that the incident laser is linearly polarized along the axis Z, perpendicular to the propagation direction.

#### 2.1.2. Re-Radiation Enhancement

The re-radiation enhancement arises from the fact that the power emitted by a dipole depends on the environment in which it is embedded (for example, a molecule will radiate differently if excited in vacuum or at the interface among different materials). This phenomenon is referred to as modified spontaneous emission and has been demonstrated in several experimental studies [118,119,120,121,122]. Its physical origin is quantum mechanical; however, it can be explained in a classical framework, resorting to the concept of self-reaction field. This subject is treated in detail in the works by Le Ru et al. [6], Novotny et al. [123], and Ding et al. [7]. Considering a dipole in vacuum or embedded in a homogenous medium, the radiated power diffuses into the space following the typical emission pattern of a dipole. However, if interfaces are present around the molecule, the electromagnetic field irradiated by the dipole is scattered at these interfaces and will be partly reflected backwards at the dipole position. This back-scattered radiation (self-reaction field) influences the way in which the dipole radiates power, which becomes then environment-dependent. The ratio between the power radiated by a dipole in an inhomogeneous environment ($P_{Rad}$) with respect to vacuum ($P_{0}$) can be written as [81,123,124]:

(6) $$M_{Rad}=\frac{P_{Rad}}{P_{0}}=1+\frac{6\pi \varepsilon_{0}}{{\left|p_{0}\right|}^{2}}\frac{1}{k^{3}}Im\left\{p_{0}^{*}\cdot E_{SR-0}\right\}$$

$p_{0}$ is the complex amplitude of the dipole, $E_{SR-0}$ represents the self-reaction field, and k is the wavevector of the emitted radiation. In vacuum, $E_{SR-0}=0$ and $P_{Rad}=P_{0}$; conversely, when the molecule is placed in an inhomogeneous environment, the presence of back-scattered radiation ($E_{SR-0}\neq 0$) causes the radiated power, $P_{Rad}$, to be different from the one that would be generated in vacuum, $P_{0}$.

**Figure 2:**
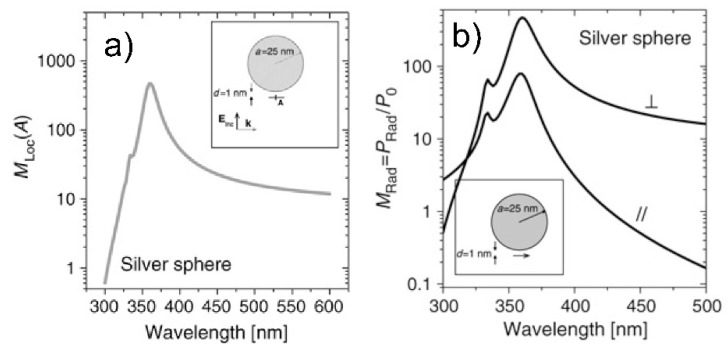
$M_{Loc}^{Z}$ ($M_{Loc}^{}$ in the figure) and $M_{Rad}$ as a function of the wavelength are shown in panel (**a**,**b**), respectively. The molecule is placed 1 nm away from the surface of the silver nanoparticle (25 nm radius), along the direction in which the excitation laser is polarized (Z). Concerning the re-emission enhancement, $M_{Rad}$ is the power emitted by the dipole integrated over the whole solid angle of emission; the molecular dipole can be oriented either parallel (∥) or perpendicular (⊥) to the surface. Reproduced with permission from Le Ru et al. [6]. Copyright (2009), Elsevier B.V.

It is worth discussing, as a case study, the enhancement of the local field and of the radiated power for a molecule placed at 1 nm from the surface of a simple silver sphere (Figure 2) [6]. One can observe that the spectral dependence of $M_{Loc}^{Z}$ ($M_{Loc}$ in the figure) and $M_{Rad}$ is very similar, even though the physical origin of the two enhancements is quite different as explained above. It is not in the scope of this review to tackle this problem in detail, however it is worth suggesting an intuitive consideration that may give an insight into the reason why the spectral shapes of the enhancements are similar. Both the external laser source and the radiation emitted by the dipole can excite surface plasmons in the substrate and; therefore, both can localize light in the spatial region where the molecule resides. This suggests that an analogy exists between the local field generated by the laser, $E_{Loc}\left(\omega_{L}\right)$ in Equation (5), and the back-scattered field, $E_{SR-0}$ in Equation (6). By means of the optical reciprocity theorem and under some conditions, it can be demonstrated that $M_{Rad}$ is equivalent to $M_{Loc}^{Z}\left(\omega_{R}\right)$. This means that the re-radiation enhancement can be recast as a local field enhancement problem with an exciting laser at frequency $\omega_{R}$ rather than $\omega_{L}$ [6,81,125]. This is very important because it allows one to work out a fairly simple expression for the $G_{SERS}^{Em}$ (Section 2.1.3) containing only the local field enhancement (at the laser and at the Raman frequency), that is much easier to simulate than the re-radiation problem.

#### 2.1.3. Expression for the SERS Enhancement in the ${\left|E\right|}^{4}$ Approximation

The electromagnetic SERS enhancement of a single molecule can be written, under the commonly adopted ${\left|E\right|}^{4}$ approximation, as follows [6,81]:

(7) $$G_{SERS}^{Em}\left(E^{4}\right)=M_{Loc}^{Z}\left(\omega_{L}\right)M_{Loc}^{Z}\left(\omega_{R}\right)={\left[\frac{E_{Loc}\left(\omega_{L}\right)}{E\left(\omega_{L}\right)}\right]}^{2}{\left[\frac{E_{Loc}\left(\omega_{R}\right)}{E\left(\omega_{R}\right)}\right]}^{2}$$

The terms $M_{Loc}^{Z}\left(\omega_{L}\right)$ and $M_{Loc}^{Z}\left(\omega_{R}\right)$ are the field enhancements generated by a laser polarized along Z at the laser and at the Raman frequency, respectively. Based on the considerations of the sections above, Equation (7) implicitly assumes that the local field and the re-radiation enhancement have the same spectral dependence, and that they are evaluated at the excitation and at the emitted frequency, respectively. Equation (7) is valid for a back scattering configuration; another hypothesis is that $M_{Loc}^{Z}\left(\omega_{R}\right)\gg M_{Loc}^{Y}\left(\omega_{R}\right)$, but this is normally true if one measures SERS with a laser polarized along the direction that provides the highest signal (Y axis is perpendicular to Z and to the propagation direction); finally, the relative orientation of the molecule and of the local field should not correspond to an unlucky combination that does not generate Raman scattering [6,81]. An example in which the wavelength dependence of $G_{SERS}$ is interpreted on the basis of the equation above is presented in the papers by McFarland et al. [126] and by Michieli et al. [127]. Equation (7) can be further simplified for small Raman shifts ($\omega_{L}≅\omega_{R}$). In this case:

(8) $$G_{SERS}^{Em}\left(E^{4}\right)≅{\left[M_{Loc}^{Z}\left(\omega_{L}\right)\right]}^{2}={\left[\frac{E_{Loc}\left(\omega_{L}\right)}{E\left(\omega_{L}\right)}\right]}^{4}$$

This expression is referred to as the zero Stokes shift limit of the ${\left|E\right|}^{4}$ approximation; it is more accurate at small Raman shifts and if $E_{Loc}\left(\omega_{L}\right)$ is not too sharp near $\omega_{L}$.

The ${\left|E\right|}^{4}$ approximation is widely applied in SERS. However, there are some specific situations in which its use is not appropriate and a more sophisticated approach is required. Typically, these cases involve the interpretation of polarization measurements (often on non-isotropic samples) [22,27,128].

An average enhancement factor can be worked out by integrating the single molecule enhancement in Equation (7) or Equation (8), over the possible orientations of the molecule and over the surface of the substrate.

### 2.2. Chemical Enhancement

Although the electromagnetic enhancement considerably exceeds the chemical enhancement in terms of magnitude; also this latter plays an important role because it determines the spectral pattern of the SERS spectra (i.e., the Raman shifts and the band intensity ratios).

The chemical effect has been studied by many authors, among which are Otto [10,11,25], Schatz et al. [129,130,131,132], Jensen et al. [133,134], Kneipp [135], and Lombardi et al. [9,136,137,138]. In this section, a brief account of the main phenomena involved is provided.

The adsorption of a molecule on a substrate can be classified on the basis of the strength of the interaction. Physisorption (physical adsorption) refers to an adsorption process in which the interaction enthalpy is less negative than −25 kJ/mol; chemisorption (chemical adsorption), instead, refers to an adsorption process in which the enthalpy is more negative than −40 kJ/mol [8,81,139]. In the former case, Van der Waals forces drive the adsorption process and; therefore, the structure of the molecule is only slightly modified. A stronger perturbation is expected in the latter case, in which a chemical bond is formed between the molecule and the surface. In both situations, although at a different extent, the electronic and geometrical structure of the molecule is altered by the interaction with the surface and, hence, the Raman cross-sections of its vibrational modes will be, in general, different with respect to those of the free molecule. The chemical enhancement can be defined as [6,8]:

(9) $$G_{SERS}^{Chem}=\frac{\sigma_{k}^{ads}}{\sigma_{k}^{free}}$$

$\sigma_{k}^{ads}$ and $\sigma_{k}^{free}$ are the Raman cross-sections of the k-th vibrational mode of the adsorbed and of the free molecule, respectively. 

Two different mechanisms can contribute to the chemical enhancement [134]:

- Non-resonant chemical effect. The interaction between the molecule and the metal does not lead to the formation of a new electronic state (the molecular orbitals lay at energies not close enough to the Fermi level of the metal); however, such interaction may induce an appreciable change in the geometrical and electronic structure of the molecule, that reveals as a mild modification of the Raman shifts and of the intensity of the vibrational modes.
- Resonant charge transfer chemical effect. The interaction between the molecule and the metal brings about the creation of a metal–molecule charge transfer (CT) state. If the Raman scattering is excited with a laser source in resonance or pre-resonance with this state, some Raman modes can be strongly enhanced, in particular those ones coupled to the allowed electronic transitions (resonant Raman scattering [111]).

The chemical effect can also originate from a “transient” charge transfer mechanism, based on a temporary electron transfer between the metal and the molecule. Its description is referred to in the literature [10], while in the following the two mechanisms above mentioned will be described in more detail.

The theoretical description of the chemical enhancement is based on two different approaches. The first is a computational approach: The properties of the molecule-metal system are studied by including ideally the whole electronic structure of the moieties. This approach allows one to work out the Raman spectra of the adsorbed species and; therefore, to reproduce precisely the shifts in the Raman bands and the changes in intensity that take place upon adsorption. Typically, density functional theory (DFT) computational methods are used and both the non-resonant and the resonant effects can be described. The second is a modelling approach: The metal–molecule system is described by an oversimplified model, in which only the features that are expected to play a role in the optical response are included. This provides a deep insight into the origin of the enhancement process, allowing one to identify the key parameters that regulate the SERS response and, for example, to predict which type of molecules and which vibrational modes are susceptible to undergo a significant enhancement. This method has been successfully used to describe the resonant enhancement.

#### 2.2.1. Computational Approach: Resonant and Non-Resonant Chemical Effect

DFT calculations are the recommended computational approach for Raman frequencies and intensities, providing pretty good accuracy at a reasonable computational cost. It is used to model the chemical mechanism of enhancement which includes three situations: (i) enhancement due to the ground state chemical interaction between the molecule and the surface (not associated with excitations of the molecule-metal system); (ii) resonance Raman enhancement with the excitation resonant with a molecular transition; (iii) charge transfer resonance Raman enhancement with the excitation resonant with a metal–molecule charge transfer transition. 

The procedure, which must be applied to the isolated molecule and to the metal–molecule system, involves several steps. Once the system has been optimized and its molecular as well as electronic structures are known, the atomic force constants and the Hessian matrix can be computed. This follows in straightforward manner, when the change in energy (E) for moving a nucleus is expressed as a Taylor’s expansion:

(10) $$E\left(R\right)=E\left(R_{0}\right)+\frac{\partial E}{\partial R}\left(R-R_{0}\right)+\frac{1}{2}\frac{\partial^{2}E}{\partial R^{2}}{\left(R-R_{0}\right)}^{2}+\frac{1}{6}\frac{\partial^{3}E}{\partial R^{3}}{\left(R-R_{0}\right)}^{3}+\ldots$$

where R is the nuclear geometry ($R_{0}$ is for the stationary point corresponding to the optimized geometry). If the energy is expanded in more than one perturbation, other than change in nuclear geometry, mixed derivatives can be calculated. In particular, the Raman signal, which, in the harmonic approximation, is the derivative of the polarizability with respect to a normal coordinate, can be expressed as:

(11) $$P_{Raman}\;\propto \;{\left(\frac{\partial \alpha }{\partial Q}\right)}^{2}\propto \;{\left(\frac{\partial^{3}E}{\partial R\partial E^{2}}\right)}^{2}$$

where Q denotes a normal coordinate and E is the electric field. First, the linear optical polarizability tensor must be calculated recomputing the DFT electronic structure in presence of a static external electric field; the polarizability far from resonance is thus obtained. If the polarizability at resonance is desired, a time-dependent DFT calculation must be carried out. The polarizability derivatives are then computed, taking into account atomic nuclear displacements or directly molecular deformations following an eigenvector of a vibration; this latter approach leads to a straight calculation of the Raman polarizability. 

The DFT modelling of Raman spectra has some limitations, related to the methodology and/or to the model system. First, due to a slight overestimation of the exchange interaction, the vibrational frequencies are overestimated by many functionals, a problem which is typically resolved by applying a scaling factor to the frequencies. In addition, when static polarizabilities are employed, the time dependence from the vibrational motions or from the laser electric field are neglected. Consequently, the result is an estimation of the non-resonant Raman properties. In order to take into account resonant effects, time-dependent DFT must be used. Numerous computational studies are limited to static Raman calculations (case (i) of the chemical mechanism).

Even in this simple case, the computational approach requires that calculations on the isolated molecule as well as on the chemisorbed molecule are performed. While the former case is straightforward, in the latter case it might be necessary to explore different arrangements for a molecule–surface complex and compute the corresponding Raman spectrum. In addition, the metal surface must be adequately described. Typically, the active sites of the metal surface are modelled as small clusters with one or few atoms, which can be considered almost isolated. For example, when a surface of silver nanoparticles is activated by coadsorption of chlorides, the active sites become positively charged and easily bind molecules with localized electronic charges, like heteroaromatic compounds. In this case, in which the Raman spectral patterns resemble those shown by Ag(I) coordination compounds, the surface can be modeled as a single silver ion [140]. In contrast, a cluster of four Ag^+^ ions is required to reproduce satisfactorily the SERS spectrum of pyrazolide absorbed on silver nanoparticles [141]. Several interesting cases are reported in Muniz-Miranda et al. [114].

Importantly, once the model size is properly tailored, a comparison with the experimental spectrum provides, indirectly, information about the binding mode. In fact, when using the eigenvectors of the vibrations, a normal mode localized in a region of the molecule far from the binding site will be almost unperturbed, while active vibrations involving the binding molecular region can even disappear from the spectrum once anchored to the surface. Another critical issue related to the finite (and small) size of the surface in the model systems is the neglect of the electromagnetic enhancement due to the excitation of the surface plasmons in the metal, as well as of the interference between the chemical and the electromagnetic effects. 

#### 2.2.2. Modelling Approach: Resonant Chemical Effect

**Figure 3:**
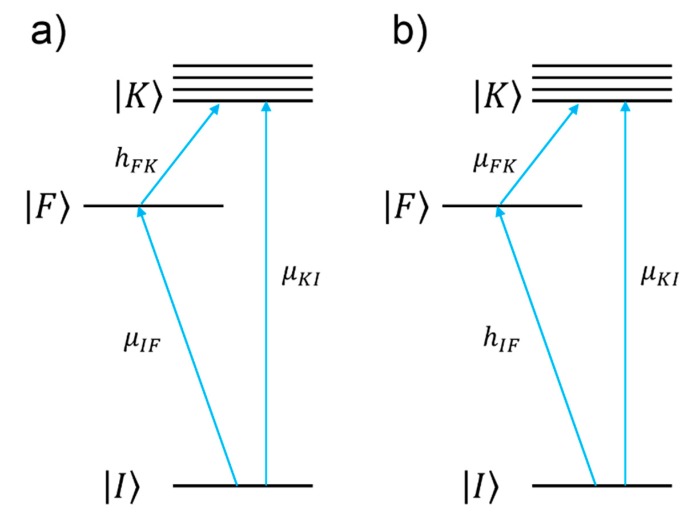
The molecule to metal and the metal to molecule charge transfer are illustrated in panels (**a**,**b**), respectively. |I〉, |K〉, and |F〉 represent the molecular ground state, the molecular excited state(s), and the Fermi state of the metal, respectively. $\mu_{KI}$, $\mu_{IF}$, and $\mu_{FK}$ are the transition dipole moments. $h_{FK}$ and $h_{IF}$ are the Herzberg–Teller coupling parameters. Reprinted (adapted) with permission from Lombardi et al. [9]. Copyright (2008) American Chemical Society.

The charge transfer chemical enhancement can be described, along with the effect of the plasmonic resonance and of the intramolecular resonance, by the theory developed by Lombardi et al. [9]. Considering a molecule attached to a metallic substrate, if the frontier orbitals of the molecule lay close enough to the Fermi level of a metal, a new electronic state with a CT character may arise. Lombardi et al. [9] highlighted that this situation is rather common for organic molecules absorbed on silver or gold; they actually demonstrated the formation of a CT state for pyridine, p-aminothiophenol, piperidine, berberine, and pyrazine on silver [137,138]. CT can occur from the metal to the molecule or vice-versa (Figure 3). For simplicity, in the following we shall illustrate the metal to molecule case, but the extension to the other case is straightforward [137].

In the framework of the Lombardi’s theories [9], for the metal to molecule CT, the SERS signal ($P_{SERS})$ of the k-th vibrational mode ($Q_{k}$) is expressed as:

(12) $$P_{SERS}\left(Q_{k}\right)\propto {\left|\frac{\mu_{KI}\mu_{FK}h_{IF}\langle i|Q_{k}|f\rangle }{\left({\left(\varepsilon^{\prime }\left(\omega \right)+2\varepsilon_{d}\right)}^{2}+\varepsilon^{\prime \prime }{\left(\omega \right)}^{2}\right)\left(\omega_{IK}^{2}-\omega^{2}+\gamma_{IK}^{2}\right)\left(\omega_{FK}^{2}-\omega^{2}+\gamma_{FK}^{2}\right)}\right|}^{2}$$

- The numerator of Equation (12) allows one to single out which vibrational modes are expected to be enhanced (“surface selection rules”).

|I〉 and |K〉 indicate the ground and excited molecular states, respectively, while |F〉 represents the Fermi state of the metal. $\mu_{KI}$ and $\mu_{FK}$ are the corresponding transition dipole moments. $h_{IF}$ is the so-called Herzberg–Teller coupling parameter [137,138], and |i〉 and |f〉 are the initial and final vibrational states. The energy level diagram of the metal–molecule system is illustrated in Figure 3.

- The denominator describes the relative contributions of the plasmonic, intramolecular, and charge transfer resonances to the observed intensity of the mode $Q_{k}$. It is composed by three factors:
  ○ The first factor, $\left({\left(\varepsilon^{\prime }\left(\omega \right)+2\varepsilon_{d}\right)}^{2}+\varepsilon^{\prime \prime }{\left(\omega \right)}^{2}\right)$, represents the plasmonic resonance. $\varepsilon^{\prime }\left(\omega \right)$ and $\varepsilon^{\prime \prime }\left(\omega \right)$ are the real and imaginary part of the dielectric constant of the metal as a function of the frequency, respectively, and $\varepsilon_{d}$ is the real dielectric constant of the medium in which the metallic structure is immersed. The medium is considered to be non-absorbing and, hence, $\varepsilon_{d}$ is real.
  ○ The second factor, $\left(\omega_{IK}^{2}-\omega^{2}+\gamma_{IK}^{2}\right)$, represents the intramolecular resonance: $\omega_{IK}$ is the transition frequency between the ground state (|I〉) and one of excited states localized on the molecule (|K〉), $\gamma_{IK}$ is a damping constant related to the bandwidth of the transition.
  ○ The third factor, $\left(\omega_{FK}^{2}-\omega^{2}+\gamma_{FK}^{2}\right),$ represents the contribution of the charge transfer state: $\omega_{FK}$ is the transition frequency between the Fermi state (|F〉) and one of the excited states localized on the molecule (|K〉), and $\gamma_{IK}^{}$ is a damping constant related to the bandwidth of the transition.

**Figure 4:**
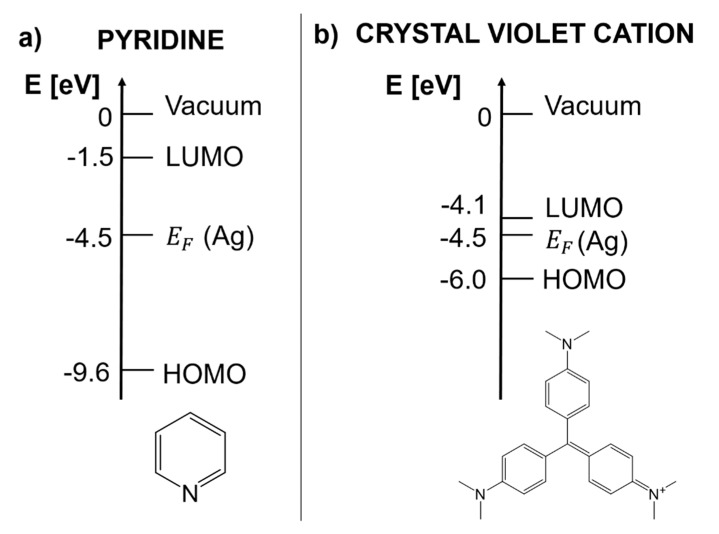
(**a**) Energy diagram for pyridine. The highest occupied molecular orbital (HOMO) energy has been approximated as the ionization energy of pyridine, determined from photoelectron spectroscopy measurements [142]; the energy difference between the lowest unoccupied molecular orbital (LUMO) and the Fermi level has been estimated with inverse photoemission spectroscopy [11]; the Fermi level of silver has been estimated by a photoelectric method [9,137,143]. (**b**) Energy diagram for the crystal violet cation. The HOMO and LUMO energies have been estimated with electrochemical methods [146].

**Figure 5:**
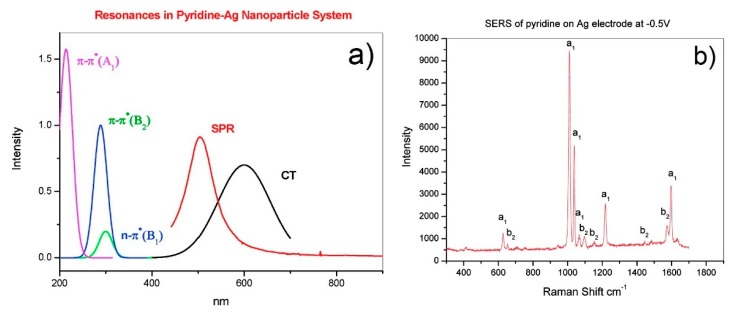
(**a**) Spectral distribution of the plasmonic (red line), charge transfer (CT, black line), and intramolecular (green, blue, and violet lines) resonances for pyridine adsorbed on silver. Reprinted (adapted) with permission from Lombardi et al. [9]. Copyright (2008) American Chemical Society. (**b**) SERS spectrum of pyridine on silver. Reprinted (adapted) with permission from Lombardi et al. [137]. Copyright (2008), American Chemical Society.

In the following, the specific example of pyridine is presented in order to illustrate which information can be extracted from this theory; pyridine is a case of metal to molecule CT [9]. Figure 4a shows the energy of the frontier orbitals of pyridine and of the silver Fermi level. The highest occupied molecular orbital (HOMO) energy has been estimated using the ionization energy of pyridine, determined from photoelectron spectroscopy measurements [142]; the energy difference between the lowest unoccupied molecular orbital (LUMO) and the Fermi level has been estimated by Otto [11] with inverse photoemission spectroscopy; the Fermi level of silver has been estimated by a photoelectric method [9,137,143]. In Figure 5a, the spectral distribution of the three resonances in the denominator of Equation (12) are shown. The peak at 520 nm represents the typical plasmonic resonance (SPR in the figure) of a small gold nanosphere (10–50 nm); the allowed intramolecular transitions of pyridine appear below 300 nm and their intensity, as a function of the symmetry of the transition, scales as B_1_ < B_2_ < A_1_ (intramolecular resonances can be experimentally measured from the extinction spectra or theoretically calculated); the CT resonance was inferred from the measurement of the SERS enhancement as a function of the excitation wavelength, in association with optical measurements of the silver substrate, bare and functionalized with pyridine [144,145]. Let us suppose that a SERS substrate functionalized with pyridine is excited with a laser resonant with the CT transition (in Equation (12), this means that $\omega \sim \omega_{FK}$). Under these conditions, the third factor in the denominator of Equation (12) becomes small and; therefore, the intensity of some of the modes ($P_{SERS}\left(Q_{k}\right))$ increases. In order to figure out which modes are more efficiently enhanced, one must look at the numerator of Equation (12) (surface selection rules). The terms $\mu_{KI}$, $\mu_{FK}$, and $h_{IF}$ establish which symmetry the involved states (ground, intramolecular, and CT) and modes ($Q_{k}$) must possess in order to make their product non-zero. It can be demonstrated that, in the case of pyridine, the vibrational modes that are enhanced are those that have the same symmetry of the allowed intramolecular electronic transitions. Moreover, since their intensity is proportional to ${\left|\mu_{KI}\right|}^{2}$, the modes that are coupled to the most intense intramolecular transitions are also the most enhanced. In fact, as shown in the experimental data in Figure 5b, the Raman bands scale in intensity as b_1_ < b_2_ < a_1_, thus following the intensity order of the electronic transitions (B_1_ < B_2_ < A_1_).

**Figure 6:**
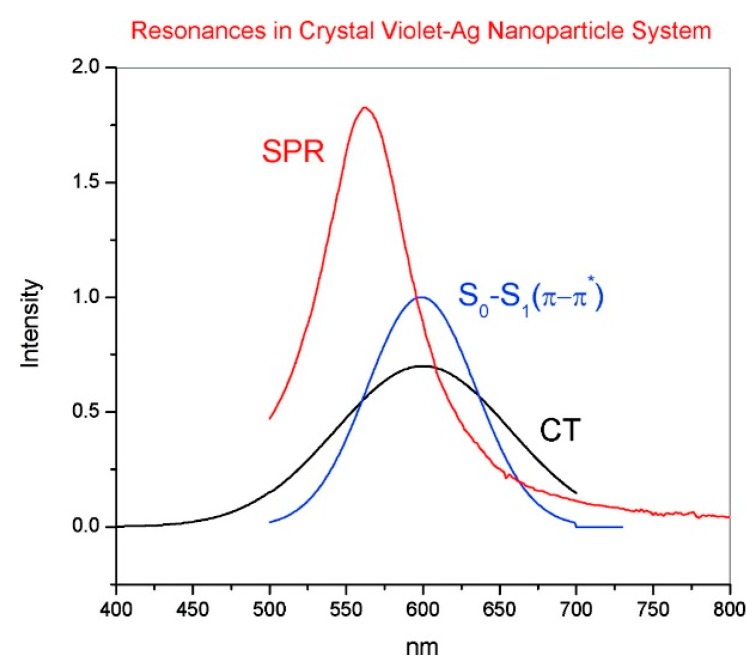
Spectral distribution of the plasmonic (red line), CT (black line), and intramolecular (blue line) resonances for CV^+^ adsorbed on silver. Reprinted (adapted) with permission from Lombardi et al. [9]. Copyright (2008) American Chemical Society.

Another instructive example is the crystal violet cation (CV^+^): It is a dye which has been often used for single molecule studies [14,146,147] and as a Raman reporter molecule [148,149], due to its very large Raman cross-section. Its energy diagram is illustrated in Figure 4b. The energy of the frontier orbitals have been estimated by means of electrochemical methods [146]. The main CV^+^ intramolecular resonance and the CT state that is formed upon interaction with silver are shown in Figure 6. It can be noticed that, once excited with the common He-Ne laser (excitation at 633 nm), both the intramolecular and the CT resonance are simultaneously active, suggesting that this dye at 633 nm is effectively a very strong Raman scatterer. CV^+^ is an example of molecule to metal CT [146].

The approach described in this section allows one to predict the possible occurrence of a CT chemical enhancement, provided that the energies of the frontier orbitals of a molecule with respect to the Fermi level of the metal are known. In this respect, the Fermi level of several metals are reported in literature [9,137,143,150]; the HOMO and LUMO energies of a molecule can be estimated experimentally, for example by cyclic voltammetry [151], or calculated, for example, via a quantum chemistry approach.

## 3. Main Factors Affecting the SERS Enhancement and Its Experimental Determination

### 3.1. Materials for SERS

SERS substrates are traditionally fabricated with materials that support the plasmonic resonance, mainly gold and silver, but also copper and aluminum (Section 3.1.1). Other types of materials are being studied to amplify optical signals, in particular semiconductors and dielectrics, and they will be briefly illustrated in Section 3.1.2.

#### 3.1.1. Metallic Materials

**Figure 7:**
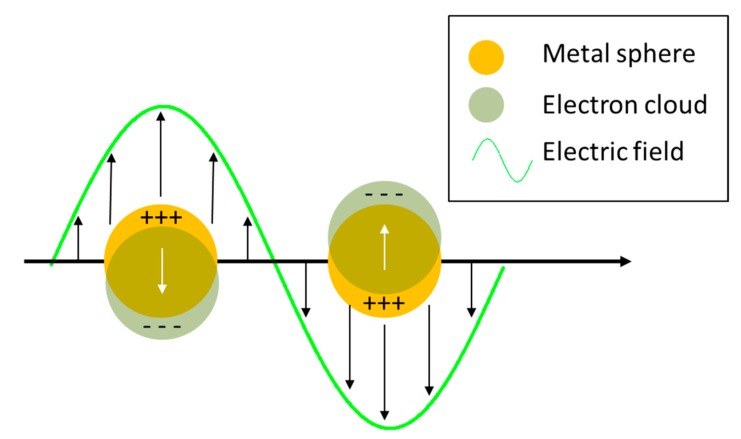
Collective oscillations of electrons in a spherical nanoparticle under the action of the external electric field.

When an electromagnetic radiation impinges on a metal nanoparticle, its conduction electrons are displaced with respect to the positive ions that form the lattice, inducing a polarization of the system; on the other hand, the Coulombic attraction between the displaced negative and positive charges acts as a restoring force. Therefore, the nanoparticle can be exemplified as a simple mass-spring oscillator, in which conduction electrons in the nanoparticle (mass) coherently oscillate, subject to the driving force of the periodic electric field and to the restoring force generated by the Coulombic attraction between the positive and negative charges (spring) [152]. This coherent oscillation is referred to as localized surface plasmon resonance. The world “localized” indicates that the electron oscillations do not propagate because they are spatially localized in three dimensions by the finite size of the nanoparticle, much smaller than the wavelength of light. Figure 7 illustrates the collective oscillations of electrons in a spherical nanoparticle under the action of the external electric field.

In addition to localized surface plasmons, also propagating plasmons exists; they can be excited, under suitable conditions, at the surface of one dimensional (i.e., nanowires) and two-dimensional metallic substrates. This review essentially deals with localized plasmons, since they are the most widely exploited to enhance Raman; the use of propagating plasmons in SERS will be briefly treated in Section 5.3.2 and Section 5.3.3, where some examples of substrates able to support them will be provided. An in-depth description of the physics of localized and propagating surface plasmons is reported in the paper by Amendola et al. [153] and in the books by Maier [154], Le Ru et al. [6], Novotny et al. [123], and Raether [155].

For a small metallic sphere (diameter much smaller than the wavelength of light), the absorption and scattering cross-sections ($\sigma_{Abs}$ and $\sigma_{Sca}$, respectively) can be calculated in the quasi static approximation, while the extinction cross-section ($\sigma_{Ext}$) is simply the sum of the two [154,156]:

(13) $$\sigma_{Abs}\left(\omega \right)=4\pi ka^{3}Im\left[\frac{\varepsilon \left(\omega \right)-\varepsilon_{d}}{\varepsilon \left(\omega \right)+2\varepsilon_{d}}\right]$$

(14) $$\sigma_{Sca}\left(\omega \right)=\frac{8\pi }{3}k^{4}a^{6}{\left|\frac{\varepsilon \left(\omega \right)-\varepsilon_{d}}{\varepsilon \left(\omega \right)+2\varepsilon_{d}}\right|}^{2}$$

(15) $$\sigma_{Ext}\left(\omega \right)=\sigma_{Abs}\left(\omega \right)+\sigma_{Sca}\left(\omega \right)$$

The term k ($k=\frac{2\pi }{\lambda }$) represents the wavevector; a is the radius of the nanoparticle; ε is the dielectric constant of the metal; $\varepsilon_{d}$ is the dielectric constant of the medium surrounding the nanoparticle, considered a non-absorbing medium. The dielectric constant of a material is in general a complex quantity that describes how the material behaves when it interacts with an external electromagnetic field:

(16) $$\varepsilon \left(\omega \right)=\varepsilon^{\prime }\left(\omega \right)+i\varepsilon^{\prime \prime }\left(\omega \right)$$

The real part $\varepsilon^{\prime }\left(\omega \right)$ describes how the system is polarized by the external field and the imaginary part $\varepsilon^{\prime \prime }\left(\omega \right)$ accounts for the losses generated during the polarization process [157]. The point is worth highlighting is that both absorption and scattering (and hence extinction) are maximized when:

(17) $$\varepsilon \left(\omega \right)+2\varepsilon_{d}≃0\;\Rightarrow \;\varepsilon^{\prime }\left(\omega \right)≃-2\varepsilon_{d}$$

Under this condition, called resonance condition, the denominator of Equations (13) and (14) is minimized and equals $i\varepsilon^{\prime \prime }\left(\omega \right)$. Concerning the field enhancement, it is worth showing the expression of the local field inside the nanoparticle ($E_{in}$), as a function of the incident one ($E_{0}$) [6,123]:

(18) $$E_{in}=\frac{3\varepsilon_{d}}{\varepsilon \left(\omega \right)+2\varepsilon_{d}}E_{0}$$

**Figure 8:**
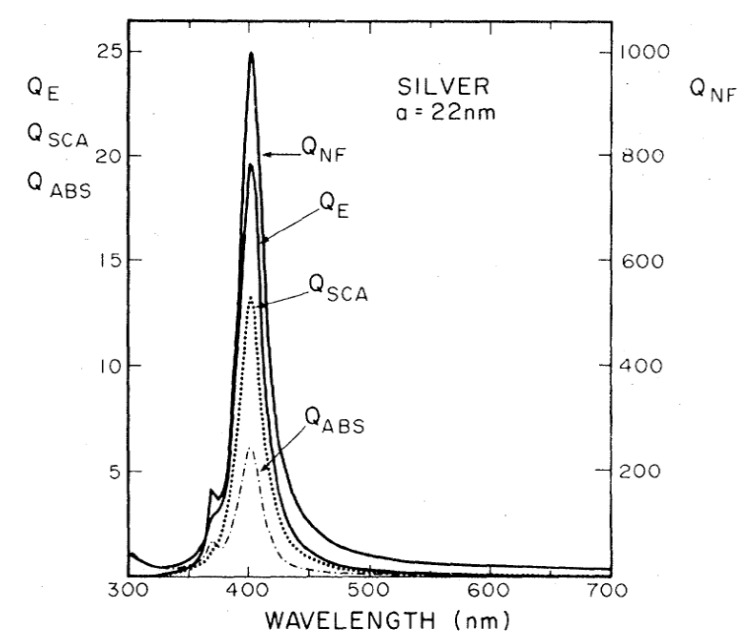
Scattering, absorption, extinction, and local field efficiencies ($Q_{SCA}\left(\omega \right)$, $Q_{ABS}\left(\omega \right)$, $Q_{E}\left(\omega \right)$, $Q_{NF}\left(\omega \right),$ respectively) for a silver nanoparticle with radius a = 22 nm immersed in water. These quantities are proportional to the corresponding cross-sections (i.e., $Q_{SCA}\left(\omega \right)=\frac{\sigma_{Sca}\left(\omega \right)}{\pi a^{2}}$ and similarly for the others). Reproduced with permission from Messinger et al. [158]. Copyright (1981), American Physical Society.

It is worth noticing that the resonance condition for $E_{in}$ is the same as for absorption, scattering, and extinction (Equations (13)–(15)). Figure 8 shows the calculated efficiencies with which a silver sphere with radius a = 22 nm converts the input power into scattering, absorption, extinction, and local field ($Q_{SCA}\left(\omega \right)$, $Q_{ABS}\left(\omega \right)$, $Q_{E}\left(\omega \right)$, $Q_{NF}\left(\omega \right)$, respectively) [158]. These efficiencies are proportional to the corresponding cross-sections (e.g., $Q_{SCA}\left(\omega \right)=\frac{\sigma_{Sca}\left(\omega \right)}{\pi a^{2}}$, and similarly for the other quantities). It is clear that, for this specific case, the resonance condition brings along an enhancement of the scattering, absorption, extinction, and local field processes, in the same spectral region. It will be shown in Section 4.1, that, in general, this is not true for more complex plasmonic systems.

In very simple terms, the link between the classical view of electron oscillations and the enhancement of the local field can be explained as follows. The polarization induced in the nanoparticle by the field is equivalent to a point dipole located at the center of the sphere. The field generated by this oscillating dipole adds up to the external one, leading to an overall field stronger than the incident one [6].

Let us analyze now which conditions have to be fulfilled for producing strong enhancement ($\left|E_{in}\right|\gg \left|E_{0}\right|$):

- Real part of the dielectric constant. Since the surrounding medium is supposed to be non-absorbing ($\varepsilon_{d}$ is real and positive), in order to fulfill the resonance condition ($\varepsilon^{\prime }\left(\omega \right)≃-2\varepsilon_{d}$) the material that forms the nanoparticle must possess $\varepsilon^{\prime }\left(\omega \right)<0$.
- Imaginary part of the dielectric constant. When the resonance condition is satisfied then $E_{in}=\frac{3\varepsilon_{d}}{i\varepsilon^{\prime \prime }\left(\omega \right)}E_{0}$: therefore, the smaller $\varepsilon^{\prime \prime }\left(\omega \right)$, the higher the enhancement.

Typically, some metals possess a negative real part of the dielectric constant and a relatively low imaginary part. This is easily realized by looking at the expression of their dielectric constant, written according to the Drude model that includes only the contribution of the conduction electrons [157,159]:

(19) $$\varepsilon \left(\omega \right)=\left[1-\frac{\omega_{p}^{2}}{\omega^{2}+\Gamma^{2}}\right]+i\left[\frac{\omega_{p}^{2}\;\Gamma }{\omega \left(\omega^{2}+\Gamma^{2}\right)}\right]$$

In this expression, the real and the imaginary parts are explicitly individuated by the square brackets; Γ is the total damping rate that accounts for all types of losses; $\omega_{p}$ is the plasma frequency, that can be written as $\omega_{p}=\sqrt{\frac{ne^{2}}{m^{*}\varepsilon_{0}}}$, where n is the density of the conduction electrons and $m^{*}$ their effective optical mass. The plasma frequencies of some metals, typically used for SERS applications, are reported in Table 2. 

**Figure 9:**
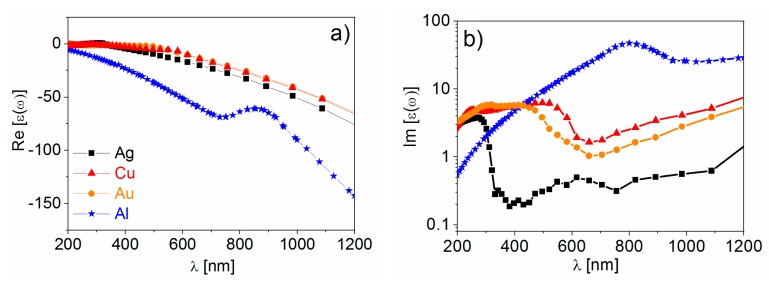
The real and imaginary part of the dielectric constant are reported in panel (**a**,**b**), respectively. The data for gold, silver, and copper are taken from Johnson et al. [162]; the data for aluminum are taken from Palik [163]. Reproduced with permission from Pilot et al. [81]. Copyright (2018), Springer International Publishing AG.

The real part of the dielectric constant is negative ($\left[1-\frac{\omega_{p}^{2}}{\omega^{2}+\Gamma^{2}}\right]<0$) for $\omega <\omega_{p}$, and this condition is met for all metals reported in Table 2 at optical frequencies. The negative sign of the real part indicates, within the spring-mass model of the plasmonic resonance, that the electrons do not oscillate in phase with the external field. This is due to the effective mass of the electrons that do not move fast enough to follow the oscillations of the external radiation [152]. Concerning the losses, the contributions are sorted out in two categories: those belonging to the conduction electrons (i.e., electron-electron and electron-phonon scattering, grain boundaries etc.) that can be accounted for in the Drude model through the term Γ; and those belonging to bound electrons, that are ascribed to transitions from the conduction to the valence band. The onset for the interband transition is reported in Table 2. At frequencies higher than $\omega_{Inter}$, losses increase remarkably, with the interband contribution becoming the most relevant. Figure 9 reports the experimental real (panel a) and the imaginary (panel b) parts of the dielectric constants of the metals mentioned above [81,162,163]. 

Looking at Figure 9, the following points are worth highlighting:

- The real part of the metals in figure is negative throughout the range 200–1200 nm and, hence, the resonance condition can be fulfilled in typical Raman excitation regions.
- The metal that possess the lowest losses in the visible region is silver, which is then the material expected to provide the largest enhancement. Going towards the near-infrared; however, the differences among silver, gold, and copper level out and the three metals are expected to perform similarly. This behavior stems from the fact that the onset of the interband transitions is around 300 nm for silver and around 600 nm for gold and copper, as evidenced by the rather steep increases of $\varepsilon^{\prime \prime }$ at those wavelengths [160]. Concerning aluminum, the onset for interband transition is located at approximately 880 nm, but in the ultra-violet (UV) region the losses are quite low and; therefore, it is considered a good material for UV SERS.

It is worth noticing that noble metals can also be alloyed with transition metals. This process allows one to “engineer” the band structure, since the presence of the transition metals can shift the plasma frequency and the threshold of the interband transitions; therefore, leading to a modification of the optical and enhancing properties of the alloy [157]. Moreover, in this way, magnetic and catalytic functions can be combined with the plasmonic ones [164,165]. SERS activity has been demonstrated, for example, for Au-Fe [164], Pd-Ag [166], and Pt-Ag [166] alloys.

So far, the discussion has regarded a comparison of different metals in terms of performance. However, other factors are also relevant in the fabrication of SERS substrates, for example, the cost, the ease of processing, the chemical stability in the environment in which they are used, the tendency to oxidation or sulfidation, and the biocompatibility. Gold and silver are the most widely employed, because they are more stable than copper and aluminum [95]. For the particular case of medical and biological applications, gold is very often the material of choice, owing to its superior chemical stability and low toxicity [167,168,169,170]. Silver tends to oxidize and to react with sulfur compounds present in the atmosphere [127,171,172,173,174,175,176]. Additionally, copper [177] and aluminum [178] form a native oxide layer in air. The presence of an oxide layer may alter the plasmonic performance in several ways, for example, it acts as a spacer between the metal and the analyte (leading to a lower amplification) and it can modify the affinity of the analyte towards the surface. The effect of oxidation on silver has been studied by several authors [127,174,175,176] and seems to be strongly dependent on the specific case under study; probably the type of analyte, the functionalization and the oxidation procedures all play a role in the SERS response. The effect of oxidation on the localized surface plasmon resonance and/or on the SERS signal of copper substrates have been studied, for example, by Chan et al. [177] on a nanopillar array fabricated by nanosphere lithography and by Muniz-Miranda et al. [179] on nanoparticles fabricated by laser ablation. Copper is an interesting alternative to the most widely employed gold and silver for its low cost; a recent review by Markin et al. [180] discusses this topic. Finally, the SERS activity of aluminum has been reported in the deep UV region (Dörfer et al. [181] and Taguchi et al. [182]), but also in the visible (Lay et al. [183]) and in the near infrared (Tian et al. [184]). An in depth discussion on aluminum plasmonics and on the issue of oxidation can be found in the papers by Gérard et al. [178] and by Knight et al. [185]. A theoretical account of the plasmonic properties of aluminum and other non-noble metals has been reported by McMahon et al. [186]. Many biomolecules, like amino acids and proteins, absorb in the UV and therefore, aluminum substrates would open the possibility of simultaneously exploiting the molecular resonance and the SERS effect to increase the sensitivity of the measurements; the price to pay is that exciting with a short wavelength radiation and working under SERS and molecular resonant conditions increase the chance of damaging the analyte [187].

#### 3.1.2. Non-Metallic Materials for SERS and Specific Mechanisms Involved in the Enhancement

In the last years, dielectric and semiconductor materials have been investigated as an alternative to metals for SERS applications [24,157,188,189,190,191,192], since they may present some advantages with respect to these latter. For example, the absorption and dissipation processes in metals lead to the release of heat that can alter or decompose the sample: Mahmoudi et al. [193] have shown that plasmonic heating can change the composition of the protein corona; therefore, causing unwanted modifications in the sample under investigation. With non-absorbing materials, although the molecule can still be subjected to strong electromagnetic fields, it does not undergo overheating and; therefore, in principle, the laser power can be raised to increase the Raman signal. Moreover, in dielectrics and semiconductors, not only the shape and the size of the nanoparticles, but also the location of band edges and the width of the band gap, can be tuned in order to optimize the enhancement. Finally, these materials offer a richer variety of functional groups that can be linked to the surface (like –COO^−^, –SH, –OH, etc.) [24].

Additionallt, with non-metallic materials, electromagnetic and chemical effects are responsible for the amplification of the Raman signal, but with some differences with respect to metals.

The electromagnetic enhancement based on the excitation of the surface plasmon resonance is not so easy to achieve in dielectrics/semiconductors. It requires, in fact, the presence of a large number of free electrons in the conduction band, that is normally not densely populated in dielectrics. Heavily doping semiconductors has been proposed as a method for increasing the electron density in the conduction band and, hence, for allowing the excitation of plasmonic resonances in the visible or near infrared. However, for silicon, germanium, and III-V semiconductors, the required level of doping is very high and the solid solubility of dopants poses a challenging problem [188]. Transparent conductive oxides (TCOs), like indium tin oxide (ITO), aluminum-doped ZnO (AZO), and gallium-doped ZnO (GZO) have been studied by Naik et al. [188], who showed that TCO nanoparticles exhibit a plasmonic resonance in between 1500 and 2000 nm (a region; however, normally not used for exciting Raman spectra). In the same paper, the plasmonic properties (absorption and field enhancement) of titanium nitride (TiN) and zirconium nitride (ZrN) nanoparticles have been calculated and turned out to be comparable to those of gold. These nitrides could be interesting as SERS materials, since they are non-stoichiometric and, hence, their composition and optical properties can be tuned [194,195]. Moreover, TiN is characterized by a good biocompatibility, hardness and thermal stability (melting point ~2900 °C) [196,197,198]. The use of TiN as a SERS material has been investigated in few papers, for example, Zhu et al. [199] studied the SERS response of Rhodamine6G on several nitride thin films (TiN, AlN and TiAlN); and Juneja et al. [196] calculated the enhancing properties of ZrN and TiN dimers, comparing the results with analogous structures made of gold.

**Figure 10:**
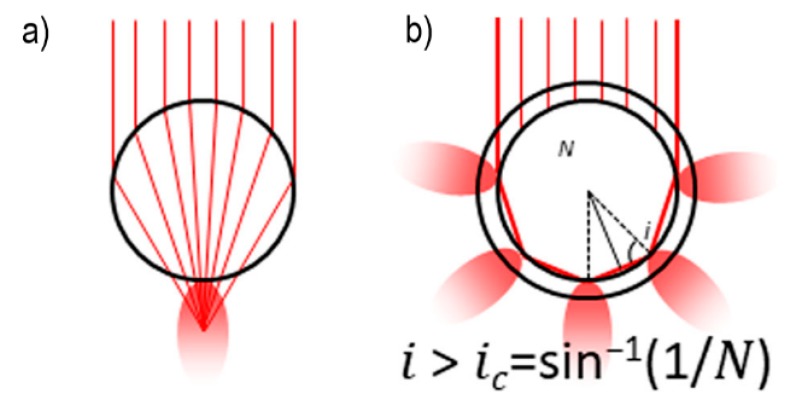
Non-plasmonic electromagnetic enhancement in dielectric nanoparticles. (**a**) A dielectric sphere acts as a microlens, focusing light; (**b**) in a core-shell dielectric resonator, light is partially trapped inside the core. The Raman signal is amplified by the evanescent waves generated at the surface of the core. Reproduced with permission from Bontempi et al. [205]. Copyright (2018), John Wiley and Sons.

Two cases of non-plasmonic electromagnetic enhancement in dielectric nanoparticles are illustrated in Figure 10. In the first case, the dielectric nanoparticle acts as a microlens, concentrating the impinging light in a small volume (Figure 10a). The second case is based on the phenomenon of Mie scattering (Figure 10b) [156,200,201]. Rayleigh scattering occurs when light impinges on objects with a size smaller than approximately $\frac{1}{10}$ of the wavelength, and; therefore, is typical of molecules; conversely, Mie scattering occurs with objects that have a size comparable to the wavelength and includes the Rayleigh one as a special case [24]. They are both elastic processes, but they differ for the diffusion pattern and for the relative scattered irradiance as a function of the wavelength [201]. It can be demonstrated that photons can be trapped inside a dielectric particle, running for hundreds of meters before the internal electric field is significantly reduced; these resonant modes are called Mie resonances, morphology dependent resonances (MDRs) or whispering gallery modes (WGMs) [24]. Evanescent waves are generated at the external surface of the particle and extend in the space for several hundreds of nm. Therefore, molecules lying close to the surface of the particle can undergo an amplification of their optical properties [24]. These two mechanisms are likely to work simultaneously in a Raman experiment, with the former or the latter prevailing, depending on the particle size and on the refractive index contrast with the external medium. The concept of hot spots, that will be illustrated for metals in Section 3.2, has a counterpart also in dielectrics and has been discussed in the paper by Bakker et al. [202]. The SERS activity of SiO_2_/TiO_2_ microbeads, arising from an electromagnetic mechanism, has been exploited by Alessandri et al. [203] to detect methylated lysine hydrochloride and by Bontempi et al. [204] to detect environmental CO_2_. The enhancement factor reported for these all dielectric enhancers is about 10^3^ [205]. Albella et al. engineered and fabricated a dimer formed by two silicon particles that exhibited a significant SERS activity, amplifying the signal from poly(methyl methacrylate) (PMMA) by a factor of 10^3^ with limited heating of the sample; the dimer was excited at 860 nm [206,207].

The chemical enhancement occurs similarly to metals and, in general, comprises a non-resonant and a resonant contribution. The formation of a CT state differs from the case of metals in two points: (a) The charge transfer does not occur to/from the Fermi level but to/from the edges of the conduction or valence band; (b) also the exciton transition in the semiconductor plays a role in defining the selection rules and the relative intensities of the Raman bands. It is worth noticing that a wide variety of semiconductors is available, with different band edges and band gaps, allowing one to tailor the chemical enhancement on the analyte of interest. A CT mechanism has been claimed as contributing to the SERS enhancement for mercaptopyridine on CdS [208] and on ZnSe [209], and for several molecules on TiO_2_ [210] and ZnO [211]. A complete account of the charge transfer mechanism in semiconductors is reported in the paper by Lombardi et al. [189].

Another category of materials that has been recently explored for Raman amplification is that of the 2D materials, in particular graphene. Ling et al. [212] studied the Raman spectra of some π-conjugated molecules (phtalocyanine, rhodamine 6G, crystal violet, protoporphyrin IX) adsorbed as sub-monolayers on a graphene foil and on a Si/SiO_2_ substrate. The authors observed that the signals on graphene were significantly stronger (about two to 17 times) than on Si/SiO_2_. This, along with the Raman-mode dependence of the enhancement and the close position of the HOMO and LUMO of the molecules, with respect to the Fermi level of the graphene, suggested the presence of a CT mechanism. This mechanism has been further confirmed in several subsequent papers [213,214,215]. Graphene enhanced Raman scattering is often referred to as GERS.

### 3.2. The Role of Hot Spots

The field enhancement distribution at the surface of a plasmonic substrate is highly inhomogeneous and mainly localized in very small spatial regions called “hot spots”. From a structural point of view, these hot spots are often identified as very sharp tips or as nanogaps between nanoparticles or between a nanoparticle and a surface [7,216,217,218,219,220], with the nanogaps remarkably more efficient in amplifying the optical signals than the sharp tips.

**Figure 11:**
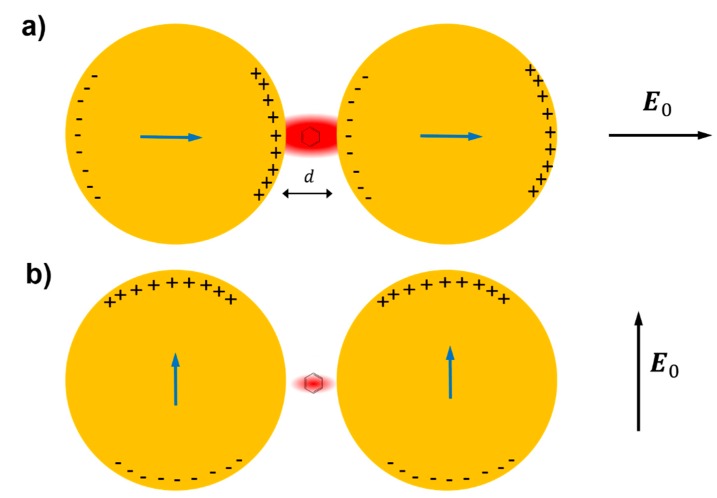
A dimer formed by two nanoparticles, separated by a gap g, is polarized by the action of an external electric field $E_{0}$; a molecule is placed in the middle of the gap. $E_{0}$ can be polarized along the main axis of the dimer (panel (**a**)) or perpendicularly to the axis (panel (**b**)). The blue arrows inside the nanoparticles represent the induced dipoles. This figure is inspired from Moskovits [221].

The reason why very strong fields are generated inside small gaps can be inferred by looking at Figure 11, which illustrates the case of a molecule placed in between two metallic spheres; in panel (a) the electric field is polarized along the main axis of the dimer, while in panel (b) it is polarized perpendicularly to the axis [221]; the electric field polarizes the nanoparticles, generating an excess of positive and negative charges on opposite sides of the nanoparticles themselves. In the on-axis polarization, one can observe that bringing the nanoparticles close to each other reduces the separation between the induced surface charges and; therefore, increases the electric field in between them. Moreover, the reciprocal interaction between the nanoparticles leads to an increase of their polarizations; in fact, each nanoparticle feels the effect of the external field plus the polarizing effect of the charges induced in the nearby nanoparticle. In other words, not only the external field, but also the induced dipole in one nanoparticle, contribute to the polarization of the other nanoparticle. Both these effects work in configuration (a) but clearly they do not in configuration (b), because the distance between the negative and positive charges on different nanoparticles cannot be made arbitrarily small and because the induced dipoles are not oriented in a way that allows their mutual reinforcement upon reduction of the gap [221]. 

**Figure 12:**
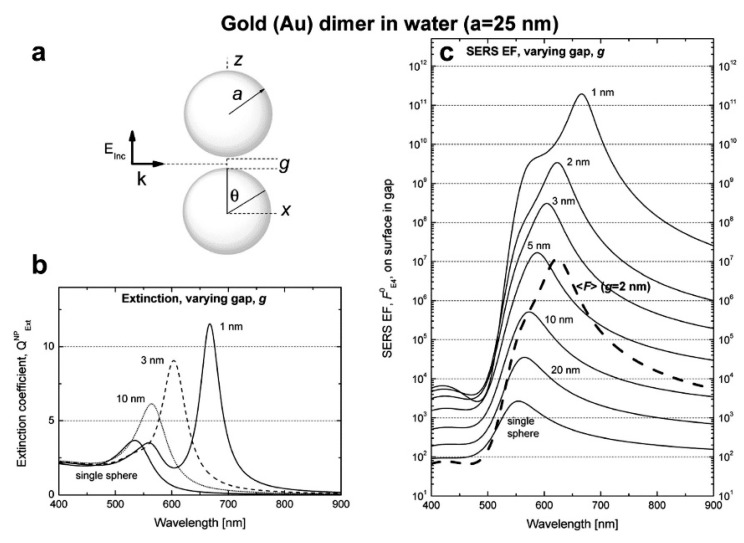
(**a**) The dimer under investigation is formed by two gold nanoparticles with radius a and separated by a gap g; the laser is polarized along the main axis. (**b**) Extinction coefficient for a single sphere and for the dimer (with different gaps) as a function of the wavelength. (**c**) Continuous lines: SERS enhancement (SERS EF in the figure) for a single sphere and for the dimer (with different gaps) as a function of the wavelength; the enhancement is calculated at the point where the surface of one of the two nanoparticles crosses the axis Z. Dashed line: SERS enhancement for the dimer with g = 2 nm, averaged over the whole metallic surface. Reproduced with permission from Le Ru et al. [6]. Copyright (2009) Elsevier B.V.

In addition to the previous qualitative considerations, it is also worth showing a specific case study in which $G_{SERS}$ is numerically calculated for different gap sizes. We shall refer to the work by Le Ru et al. [6], who studied the dimer reported in Figure 12a. The dimer is formed by two gold nanoparticles with radius a = 25 nm immersed in water and separated by a variable gap *g*. The laser is polarized along the main axis and the probe molecule is placed at the surface of one of the two nanoparticles, along the main axis. In Figure 12b,c, the extinction and the enhancement spectra of a single nanoparticle and of the dimer (with different gaps) are shown, respectively. The points worth highlighting, concerning especially the local field, are the following:

$G_{SERS}$ vs. gap size

- $G_{SERS}$ strongly increases by reducing the gap size, in particular it amounts to $\sim 5\cdot 10^{5}$ at g = 10 nm and to $\sim 3\cdot 10^{9}$ at g = 2 nm; the power law dependence is reported to be approximately $G_{SERS}\sim \frac{1}{g^{2}}$ [222,223,224]. A single gold sphere is limited to $\sim 2\cdot 10^{3}$. This behaviour explains why SERS is very often observed on aggregated nanoparticles and rarely on isolated nanoparticles. There are only very few cases in which aggregation inhibits or weakly enhances the Raman scattering; this may occur for example with hollow nanoparticles, because in this case the field enhancement generated between the nanoparticles can be counteracted by a reduction of the field inside the nanoparticles [81,225,226].
- For very small gaps (*g* < 1 nm), quantum mechanical phenomena, like electron tunneling, come into play, limiting the increase in the field enhancement. This subject has been recently studied by Zhu et al. [23] and by Hajisalem et al. [227].

$G_{SERS}$: single point or surface averaged?

- An important distinction regards the use of a surface averaged or a single point $G_{SERS}$: for example, $G_{SERS}$ at g = 2 nm, calculated at the intersection of the Z axis with the surface of one of the nanoparticles, amounts to $\sim 3\cdot 10^{9}$ but, if averaged over the surface of the dimer, it is 300 times lower (Figure 12c). This suggests that the field is strongly localized in a small spatial region; this point will be described more in detail in Section 3.2.1.

#### 3.2.1. Anatomy of a Hot Spot: Spatial Distribution of the Local Field

**Figure 13:**
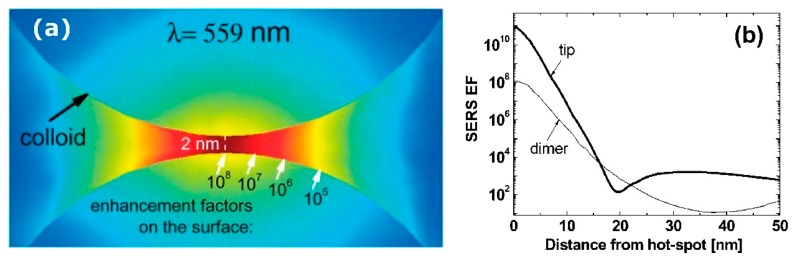
(**a**) $G_{SERS}$ distribution inside the 2 nm gap formed by two gold nanoparticles with a radius of 30 nm. The enhancement is calculated at the wavelength at which it reaches its maximum value. (**b**) Variation of $G_{SERS}$ along the (curved) surface of the nanoparticle (thin black line); the thick black line is not commented in this paper. Reproduced with permission from Etchegoin et al. [16].

It is interesting to look at how the local field is spatially distributed inside a hot spot: Two gold nanospheres with a radius of 30 nm and separated by a gap of 2 nm have been investigated, as a case study, by Etchegoin et al. [16]. Figure 13a shows, with a color map, the field intensity in the gap region, calculated at the wavelength at which the enhancement reaches the maximum. Instead, in Figure 13b it is shown numerically how the enhancement varies from the maximum as a function of the distance, along the curved surface. The three main points can be summarized as follows:

- $G_{SERS}$ varies dramatically as a function of the position, with significant variations with respect to the molecular scale. Considering the packing density of a typical SERS molecule (benzenethiol) on metals, whose maximum reported value is $6.8\cdot 10^{14}$ molecules/cm^2^ [126,228,229,230], it can be estimated that a single molecule occupies a spot with a diameter of 0.4 nm. This means that, at about 5 nm from the hottest point (corresponding to about 10 molecules), $G_{SERS}$ is already 10 times lower.
- Due to the strong spatial variations, the average enhancement is much lower than the maximum one:; in the example in Figure 12 the average is about 300 times lower that the maximum value.
- Typically, 0.64% of the surface (which means 0.64% of the molecules assuming uniform coverage) generates most of the SERS (let us say 80%) [231].

These considerations suggest the importance of creating hot spots and placing molecules inside them in order to achieve high SERS signals. 

Experimental evidence of the role played by the hot spots can be found for example in the papers by Camargo et al. [232] and by Chen et al. [233]. Camargo et al. [232] fabricated SERS substrates formed by isolated silver nanocubes and by nanocube dimers with very narrow gaps, deposited on silicon. Single nanocubes functionalized with 4-methylbenzenthiol (4-MBT) exhibited a SERS signal that, after treatment with oxygen plasma disappeared, due to the removal of the 4-MBT molecules. In contrast, in the case of the dimer, after functionalization and plasma etching, the SERS signal exhibited only a small reduction. This was attributed to the fact that plasma etched only the molecules on the outer surfaces of the dimer, but not those inside the gap. This indicated that most of the SERS signal was generated by molecules inside the gap. Chen et al. [233] fabricated nanoslits with a variable gap, narrower at the bottom and wider on top, and selectively deposited a Raman probe (amorphous carbonaceous nanoparticles) at the bottom, in the middle, and on the top edge of the slit. They observed that the SERS signal remarkably increased in the order top edge << middle << bottom, demonstrating the strong dependence of the SERS signal on the gap size. 

### 3.3. Distance Dependence of the Electromagnetic Enhancement

Electromagnetic considerations provide the following dependence of $G_{SERS}^{Em}$ as a function of the distance (d) from the surface of a spherical nanoparticle of radius a [8]:

(20) $$\frac{G_{SERS}^{Em}\left(d\right)}{G_{SERS}^{Em}\left(0\right)}={\left[\frac{a}{a+d}\right]}^{12}$$

It is worth noticing that the distance dependence of the SERS signal is different from the enhancement dependence, since the former accounts also for the number of illuminated molecules in shells at distance d from the surface, number that scales as ${\left(a+d\right)}^{2}$ [234]:

(21) $$\frac{P_{SERS}\left(d\right)}{P_{SERS}\left(0\right)}={\left[\frac{a}{a+d}\right]}^{10}$$

The above formulas suggest that the SERS enhancement and the signals drop very fast from the surface; the analyte should normally be placed within 10 nm from the surface to efficiently exploit the plasmonic effect.

Several papers have been devoted to the experimental investigation of the distance dependence of SERS. In a typical paper, the SERS enhancement or the signal of a probe molecule is measured as a function of its distance from the substrate (d); the separation distance is tuned by using variable thickness spacers, like polymers, long chain thiols, etc. The fit of the data provides a value for the parameter a, which is then compared to the size of the roughness features of the substrate, measured, for example, by atomic force microscopy (AFM). 

**Figure 14:**
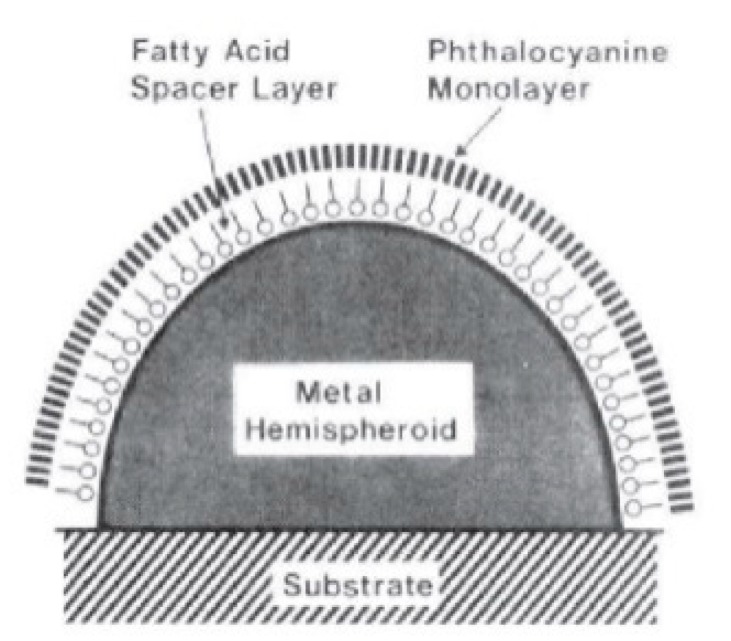
The metallic substrate is represented by the hemispheroid; on top of it, the arachidic acid layer (spacer) and the phthalocyanine (Raman probe). Reproduced with permission from Kovacs et al. [235]. Copyright (1986), American Chemical Society.

For example, Kovacs et al. [235] used a monolayer of arachidic acids of variable length as a spacer to tune the distance between a phthalocyanine and the surface of substrate formed by silver islands (Figure 14).

**Figure 15:**
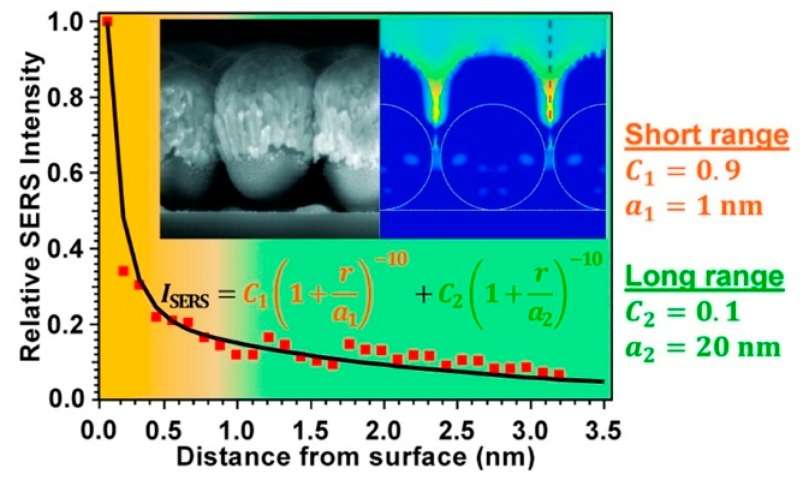
SERS signal as a function of the distance from the surface. A short and a long-range component are identified; they are associated to morphological features of the metallic substrate with a size of approximately 1 nm and 20 nm, respectively. In the insets, a scanning electron microscopy (SEM) picture of the SERS substrate (silver film over nanospheres) and a simulation of the electric field are presented. Reproduced with permission from Masango et al. [236]. Copyright (2016), American Chemical Society.

Masango et al. [236] used Al_2_O_3_ deposited by atomic layer deposition as a spacer (Figure 15); this method allowed a very precise control (with Angstrom resolution) of the distance between the SERS substrate (Ag film over silica nanospheres, Ag-FON) and the Raman probe (trimethyl aluminum).

Table 3 summarizes some experimental studies on this topic, providing the SERS substrates, the type of spacer, the Raman probe used and the distance range explored.

### 3.4. Experimental Determination of the SERS Enhancement

The enhancement is probably the most widely used figure of merit to compare the performance of different SERS substrates. In practical applications, like the detection of chemicals, it is related to the sensitivity and to the speed with which the analysis can be carried out. Its quantification is important also in structure-property studies, in which a relation between the morphology of the substrate and its SERS performance is sought and can possibly be rationalized with the support of calculations [6,26,80,116,127,241]. Moreover, if the enhancement is high enough, fundamental single molecule studies can be carried out [14,16,147,231,242,243]. However, the estimation of the SERS enhancement in literature suffers from wide discrepancies, mainly due to different definitions of the enhancement and to the way in which it is experimentally measured [244]. Concerning the definitions, a complete account of them is provided in the paper by le Ru et al. [244]; an important distinction regards the difference between a single molecule and a surface averaged enhancement. The former depends on the local field only at a specific point of the substrate (most likely a hot spot); conversely, the latter is the mean value over a certain area of the substrate. The difference between the two is substantial. This can be inferred from the case study of a nanoparticle dimer presented in Section 3.2, where it has been shown that the maximum value of the enhancement inside the hot spot is 300 times larger than the enhancement averaged over the whole surface. Moreover, an experimental study by Fang et al. [245] has quantified the spatial localization of the field on a silver coated self-assembled monolayer of polymer beads (silver film over nanosphere, Ag-FON [117]), functionalized with benzenethiol. They showed that the molecules experiencing an enhancement >$10^{9}$ (0.01% of the total molecules) account for 25% of the SERS signal and that molecules with enhancement >$10^{6}$ (6% of the total molecules) account for 85% of the SERS signal, providing experimental evidence that only a very small fraction of molecules generate most of the SERS signal.

The single molecule enhancement factor can be carried out resorting to the bi-analyte SERS method (BiASERS) [246,247] or to temperature-dependent vibrational pumping measurements (TDVP) [248,249]. BiASERS allows one to isolate single molecule events through the analysis of a large number of SERS spectra from a nanoparticle solution (or a solid substrate), in which two analytes have been introduced. Most SERS spectra will show bands from both compounds, but some of them can show signal purely from one analyte; this occurrence is taken as an indication that the signal comes from a very small number of molecules. The normalization of the single (or few) molecule signal to the signal of a reference compound provides an estimation of the single molecule enhancement [244]. TDVP is based on the idea that, if the SERS effect is strong enough, the first vibrational level of a molecule can be significantly populated by SERS itself, leading to a change in the anti-Stokes–Stokes ratio ($\rho_{k}^{aS/S})$ that depends on the cross-section of the vibrational mode under study; this vibrational pumping effect can be discriminated from the sample heating, that influences $\rho_{k}^{aS/S}$ as well, by looking at the temperature dependence of $\rho_{k}^{aS/S}$ starting from very low values (approximately 10 K).

From now on we shall focus on the average enhancement, which is the most relevant for applications. To fix ideas, we refer to the specific case in which benzenethiol is used to functionalize the solid SERS substrate and also as a reference in a normal Raman experiment. This molecule is very often used because its Raman spectrum is very well characterized, it possesses a large cross-section owing to its aromatic ring, it is non-resonant in the visible and near-infrared regions, it is stable, it strongly binds to silver and gold, and its packing density has been estimated in several papers (typically it forms a monolayer on metal surfaces) [126,228,229,230]. A common equation used to estimate the average $G_{SERS}$ is [216,244,250]:

(22) $$G_{SERS}=\frac{P_{SERS}}{P_{Raman}}\frac{N_{Raman}}{N_{SERS}}$$

$P_{SERS}$ and $P_{Raman}$ are the SERS and the normal Raman signals measured; they should be measured under the same experimental conditions (instrument, objective, etc.) and normalized by the laser intensity and the integration time, if they are not the same in the SERS and Raman experiment. $N_{Raman}$ and $N_{SERS}$ are the number of molecules illuminated by the laser in the normal Raman and in the SERS experiment. Benzenethiol is a transparent liquid, hence $N_{Raman}$ can be easily calculated if the scattering volume of the Raman instrument is known; this can be determined with good accuracy by following the procedure described in several papers [244,250,251]. The determination of $N_{SERS}$, instead, is much trickier and this term is the most important source of error (or discrepancies) in the determination of $G_{SERS}$. In the specific case of benzenthiol, or any other molecule that forms a monolayer on the substrate, in order to calculate the number of molecules illuminated by the laser, one should know the morphology of the sample (provided that the packing density is known from literature). A precise estimation may be difficult since the nanometric roughness that strongly contributes to the SERS signal is not easy to trace. In the case of samples fabricated with lithographic methods, the surface can be determined from the geometry of the sample, assuming a negligible contribution from the roughness [252]. Instead, if the sample has been prepared by assembling nanoparticles on a surface, the geometry can be estimated with microscopy techniques; however, when complex geometries are involved, a better solution is to use the underpotential deposition (UPD). UPD is an electrochemical method that consists in the deposition of a monolayer of a foreign element, which gives a specific interaction with the SERS active material. For example, the surface area of gold nanostructures can be determined from cyclic voltammetry measurements by integrating the charge under the cathodic peaks for the reduction of oxygen adsorbed on a gold electrode (*Q_UPD_*). The charge density corresponding to the formation of a complete monolayer of chemisorbed oxygen on gold is $q_{m}$ = 400 μC·cm^−2^; as a consequence, the surface area can be easily determined as $A\;=\;\frac{Q_{UPD}}{q_{m}}$. Similarly, the UPD of Pb^2+^ or Cd^2^^+^ allows the determination of the surface area of silver or copper substrates [253,254]. UPD provides a very accurate estimation of the substrate surface, since the chemisorption of oxygen or the deposition of Pb^2+^ or Cd^2+^ follows, precisely, the morphology of the substrate, also in parts of the sample difficult to access for microscopy methods; moreover, it easily applies to large area substrates (i.e., ~ cm^2^). UPS requires the SERS active features to be deposited on a conductive substrate, like silicon, ITO, metals, etc. 

The cases described so far consider the evaluation of the enhancement of a solid substrate. In the cases in which the substrate is a solution of nanoparticles, the ratio $\frac{N_{Raman}}{N_{SERS}}$ simply corresponds to the ratio of concentrations of the test molecules in the normal Raman and in the SERS experiment (provided that the same volume is illuminated). In the determination of $P_{SERS}$ and $P_{Raman}$, it is necessary to account for the self-extinction effect. The nanoparticles in solution, in fact, absorb and scatter the laser and the emitted Raman photons, for example, Weber et al. [255] used the Raman bands of methanol as an internal standard to account for the self-extinction from the solution.

It is also worth mentioning that, often, dyes like Methylene Blue, Rhodamine or CV^+^ are used for the measurement of $G_{SERS}$ of solid substrates and of colloidal solutions [256]. In this case, the same dye should be used as a reference (for example drop casting a known amount of dye on a non-SERS active surface or making a solution at a known concentration of the dye). Due to the lack of amplification; however, this type of measurement could be not so easy to carry out; it is possible to use other references, provided that the cross-section ratio between the compound used for the SERS and for the reference measurements is known. In this respect, Le Ru et al. [244] have measured the relative cross-section of Rhodamine 6G, CV^+^, 3-methoxy-4-(5′-azobenzo-triazolyl) phenylamine (BTZ), and benzotriazole (BTA) with respect to 2-bromo-2-methylpropane (2B2MP). 2B2MP is a (non-toxic) liquid compound and its Raman spectrum is faster to record compared to the spectrum of dyes in solution or deposited on non-SERS active substrates.

Another possible critical point is the choice of the Raman band for the calculation of the enhancement. The chemical effect can introduce significant differences in the amplification of the Raman bands, depending on their symmetry; this has been shown for pyridine and other molecules in Section 2.2.2. It is also worth mentioning the specific case of benzenethiol: It has been experimentally shown that the enhancement measured with the band at ~1000 cm^−1^ ($\beta_{CCC}$) is an order of magnitude smaller than the enhancement measured at ~1079 cm^−1^ ($\beta_{CCC}+\upsilon_{CS}$) [126,257,258,259]; β and υ represent the in-plane bending and the stretching modes, respectively [260,261]. This has been rationalized by Zayak et al. [257], who recognized that the Raman modes with the highest chemical contribution are the ones that induce the largest shift in the HOMO energy of benzenethiol (e.g., by breaking the conjugation of the HOMO). Additionally, the electromagnetic enhancement depends on the Raman shift, since the local field is wavelength dependent; this dependence is normally of small entity if modes lying at a few hundreds of cm^−1^ from each other are considered. 

The decomposition of the test molecule or its conversion into other species under laser irradiation (due to overheating and/or to the very intense electric field to which they are subject in the hot spots) may represent another issue in the determination of the enhancement or, more in general, for the collection of SERS spectra. It is well known that the decomposition of organic molecules leads to the formation of carbonaceous materials, with bands around ~1350 cm^−1^, ~1580 cm^−1^, and ~1500 cm^−1^ which correspond to the D and G bands of graphite-like compounds, and to amorphous carbon, respectively [262,263]. These bands can interfere with the bands of the analyte, form a broad background, and often appear as a fluctuating contribution to the spectra that is difficult to subtract [264]. The stability strongly depends on the nature of the molecule, for example, benzenethiol shows good photostability under visible and near-infrared excitation, while 1-naphtanethiol exhibits an increased tendency to decompose, going from a near-infrared to a visible excitation [263]. A widely investigated case is that of p-aminothiophenol (PATP), a very common SERS molecule. When absorbed on silver or gold and under laser illumination, it can dimerize forming an azo compound (4,4-dimercaptoazobenzene, DMAB) [265,266,267,268,269,270]. This reaction causes the SERS spectrum to be laser intensity dependent, since at increasing intensities the distinctive Raman bands of DMAB, located at 1140, 1388, and 1438 cm^−1^, appear (the appearance of the characteristic DMBA bands has been interpreted in the past as the chemical (charge transfer) enhancement of the PATP bands with symmetry b_2_ [266]). Moreover, the two main bands of PATP, located at about 1075 and 1594 cm^−1^ may be broadened due to the rise of closely lying Raman bands of DMBA. A similar phenomenon has been evidenced for p-aminobenzoic acid on silver island films, which forms p,p′-azodibenzoate under laser illumination [271]. Generally speaking, the formation of new (unknown) compounds should be avoided, since they might be resonant with the laser excitation and; therefore, they could possess grossly different Raman cross-sections compared to the starting molecule. The decomposition of molecules can be limited with several strategies. One option is working in solution rather than on solid substrates, since the solvent can efficiently reduce overheating. Another option is raster scanning the sample under the laser, to reduce the exposure time at a single point. Alternatively, one could work with setup arrangements that allow working at lower laser intensities, for example the line focus configuration [250,272], the defocusing method [37], or the two-lens back-focal-plane beam-expander combination [273].

## 4. Factors Affecting the Choice of the Excitation Wavelength in a SERS Experiment

**Figure 16:**
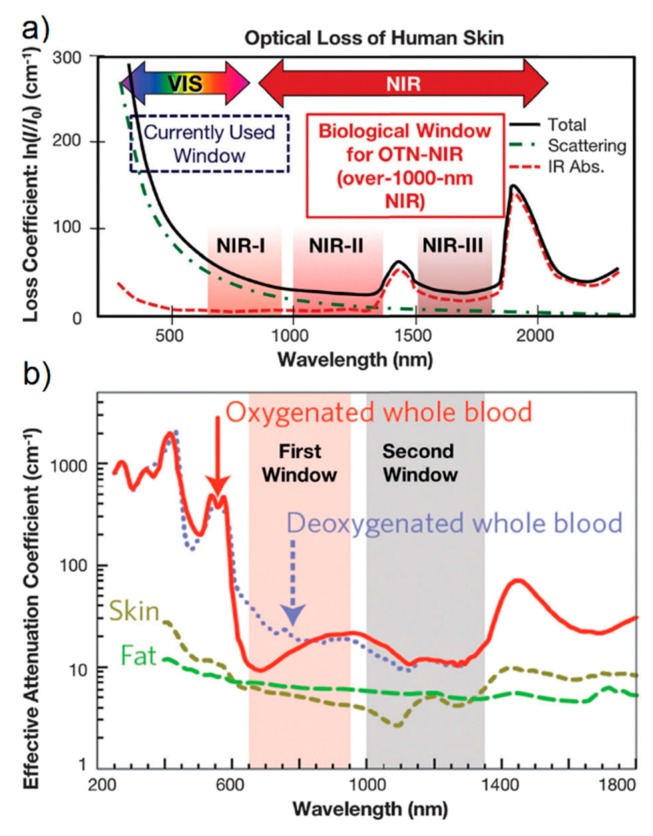
(**a**). Absorption (dash red line), scattering (dash-dot green line), and extinction (solid black line) of human skin as a function of the wavelength. The three transparency windows are indicated as NIR-I, NIR-II, NIR-III. Reproduced with permission from Hemmer et al. [276]. Copyright (2013), the Royal Society of Chemistry. (**b**) Extinction coefficient of oxygenated blood (solid red line), deoxygenated blood (dotted blue line), skin (ochre dash line), and fatty tissues (green dash line). Reproduced with permission from Smith et al. [277]. Copyright (2009), Macmillan Publishers Limited.

A relevant question for any practitioner regards which wavelength is best suited for carrying out a SERS experiment. Two important considerations to take into account are the following [274,275]:

- Especially for biomedical applications, SERS spectra should be collected with an excitation wavelength that can propagate through the tissues. As shown in Figure 16a, there are three different spectral windows in which extinction is minimized: the first one is in between 650 and 950 nm (NIR-I), the second one from 1000 to 1350 nm (NIR-II), and the third one in between 1500 and 1800 nm (NIR-III) [275,276]. The individual contribution of human skin, blood, and fatty tissues to extinction is reported in Figure 16b [277]. The first window, compared to the other two, presents a higher level of tissue auto-fluorescence that adds up to the Raman signal as a broad background, reducing the signal to noise ratio (SNR).
- SERS spectra should be collected in the experimental conditions that optimize SNR. This has as obvious consequences: faster analysis and lower limits of detection. The SERS signal for dispersive Raman instruments, that are the most widely used [19], can be expressed as:
  (23) $$P_{SERS}=G_{SERS}P_{Raman}=G_{SERS}FT_{O}Q_{D}N\sigma_{k}I$$

With respect to Equation (1), the constant K has been expressed using several factors: F is the fraction of photons emitted by the sample that are collected by the microscope objective, $T_{O}$ is the trasmittance/reflectance of all the optical components that drive the Raman signal from the sample to the detector (i.e., objective, lenses, mirrors, beam splitters, spectrograph, etc.), and $Q_{D}$ is the quantum efficiency of the detector; N, $\sigma_{k}$, and I are the number of illuminated molecules, the total Raman cross-section of the k-th mode of the molecule under investigation, and the laser intensity, respectively. In the following, the wavelength dependence of $G_{SERS}$, instrument sensitivity ($FT_{O}Q_{D}$), analyte cross section ($\sigma_{k}$), and the effect of fluorescence backgrounds are discussed. 

### 4.1. SERS Enhancement

Ideally, Raman spectra should be collected at the excitation wavelength at which $G_{SERS}$ is maximum. Its spectral dependence, which is difficult to predict, is typically probed by means of wavelength-scanned SERS. In this experiment, $G_{SERS}$ is measured at a number of excitation wavelengths sufficient to reconstruct its trend; despite being conceptually simple, this experiment requires a very specialized and expensive equipment comprising tunable laser sources and triple spectrographs [19,127,225,252]. As laser sources, mixed gas lasers (Ar^+^/Kr^+^) are often used, because they provide several discrete lines in the visible. In the near-infrared, the most flexible solution is using continuous wave titanium–sapphire lasers, whose emission is tunable in this region. Triple spectrographs, rather than notch filters, are required when the experiment involves a lot of excitation lines; the former allows one to select the rejection wavelength (e.g., elastic scattering must be efficiently suppressed in Raman experiments [19]), while the latter are fabricated to reject only a single wavelength. For well-defined geometries, typically fabricated by lithographic methods like EBL, $G_{SERS}\left(\lambda \right)$ can also be predicted by means of plasmonic simulations [6,80,241].

**Figure 17:**
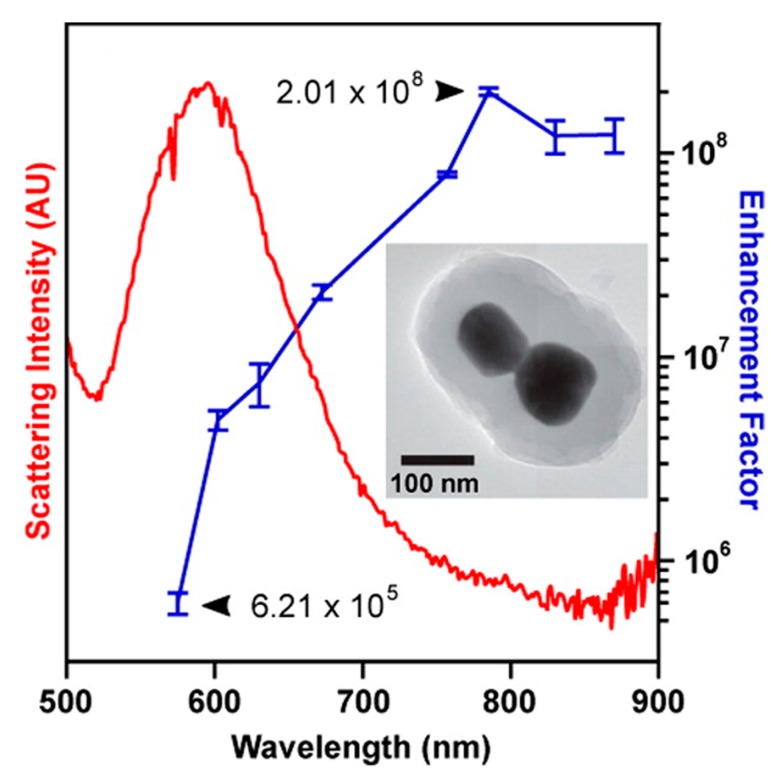
Scattering intensity (red line) and enhancement factor (blue points) measured for the single gold nanoparticle dimer embedded in a silica shell shown in the inset. Reproduced with permission from Kleinman et al. [26]. Copyright (2013), American Chemical Society.

A question that may arise is whether a way to predict the $G_{SERS}$ spectrum exists on the basis of more easily accessible observables, like extinction, scattering, or absorption (where extinction is the sum of scattering and absorption). Extinction and scattering can be easily determined with commercial instruments and, if necessary, their measurement can be implemented in a microscope to improve spatial resolution [256]. Absorbance can be selectively distinguished and measured by means of photoacoustic spectroscopy. This technique reconstructs the absorption of nanoparticles in solution from the heat (acoustic) wave that is generated after absorption of light and its conversion into thermal energy [278]. Unfortunately, it is normally assumed that no simple relation exists between the local field and the far field spectra. A striking example has been provided by Kleinman et al. [26] who studied single dimers of closely spaced nanoparticles coated with trans-1,2-bis(4-pyridyl)-ethylene (BPE) and encapsulated into a silica shell. The local field and the scattering spectrum peak in completely different spectral regions, with the former strongly red-shifted with respect to the latter (Figure 17). This behavior has been confirmed by calculations in the same paper and is consistent with the theoretical results reported by Le Ru et al. [6] for a dimer (Figure 12). Looking at Figure 12b, it is clear that the dominating extinction resonance of a dimer (with g = 1 nm for example) is red-shifted compared to the one of the single nanoparticle, but their intensities are not very different. In Figure 12c, it is shown that, similarly, the maximum enhancement of a dimer is red-shifted compared to one of the single nanoparticle, but in this case the intensity changes by many orders of magnitude. Hence, the formation of a dimer provides limited changes in the intensity of the extinction but huge effects on the enhancements. Absorption/extinction is a bulk property that depends on the volume of the nanoparticle [116], and is not much influenced by the interaction that occurs when nanoparticles are brought close to each other; on the other hand, the local field is a surface property, and the formation of a gap strongly localizes the field into it, bringing about a huge enhancement of the optical response. This topic is explained in detail in the paper by Le Ru et al. [116]. On the basis of these considerations, it can also be understood why, in solutions of nanoparticles, extinction and enhancement are typically uncorrelated. This is due to the presence of aggregates in solution, a phenomenon which is very difficult to avoid completely. If their amount is limited, the extinction spectrum will be dominated by the single nanoparticles; however, since aggregates amplify the Raman signal far more efficiently than isolated nanoparticles, the local field will be dominated by their response, which is significantly red-shifted compared to one of isolated nanoparticles [255,279,280,281,282,283].

**Figure 18:**
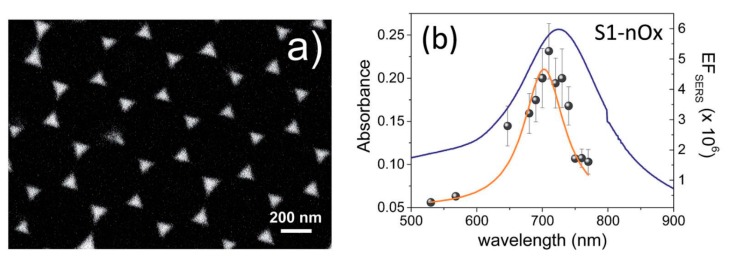
(**a**) SEM image of the SERS substrate fabricated by nanosphere lithography; (**b**) Extinction (blue line) and local field (dots and corresponding fit) distribution. Reproduced with permission from Michieli et al. [127] under Creative Commons 3.0 license (https://creativecommons.org/licenses/by/3.0/).

It is worth mentioning also some substrates in which local and far field spectra are correlated, like isolated nanoparticles or arrays of weakly interacting objects. Messinger et al. [158] showed, by simulations, that in single nanoparticles of small size, extinction, scattering, absorption, and local field peak at a very similar wavelength (Figure 8). McFarland et al. [126] and Michieli et al. [127] experimentally measured the extinction spectrum and the local field distribution of an array of nanotriangles fabricated by nanosphere lithography (Figure 18); the two are closely related and only slightly shifted with respect to each other. This small shift arises from the fact that the SERS enhancement is proportional to the field enhancement at the laser and at the Raman frequency; therefore, the maximum SERS amplification is achieved when their product is optimized (Equation (7)). Supposing that the enhancement follows the extinction spectrum, the previous condition occurs when the extinction maximum is in between the laser wavelength and the (absolute) wavelength corresponding to the Raman band used to work out the enhancement; moreover, it has been demonstrated that the lower the Stokes shift of the Raman band, the smaller the spectral shift between the local and the far field [126].

In Table 4, several contributions on this topic are summarized, including the ones that have been discussed above. 

Finally, another point that should be mentioned is that also the density of hot spots on a substrate influences the magnitude of the SERS signal measured. Let us suppose that $G_{SERS}$ is defined counting only molecules that reside in the hot spots and that two substrates, one with a higher and another one with a lower density of hot spots, are fabricated. In this case, the two substrates will have the same $G_{SERS}$ but, clearly, the one in which hot spots are more densely packed will provide the stronger SERS signal. Moreover, the SERS signal is linear with the laser intensity; in principle the intensity can be raised until the molecules or the substrate do not get damaged by photochemical or thermal processes. Therefore, the SERS signal that can be extracted does not depend only on $G_{SERS}$, but also on the density of hot spots and on the damage threshold of the molecule/substrate system. 

### 4.2. The Analyte Cross-Section 

Considering, first, the case in which the excitation laser is not resonant with any of the allowed electronic transitions in the molecule, it can be demonstrated that the Raman cross-section of a certain Raman mode (k) varies as [19]:

(24) $$\sigma_{k}\propto {\tilde{\nu }}_{0}{\left({\tilde{\nu }}_{0}-{\tilde{\nu }}_{k}\right)}^{3}\sim {\tilde{\nu }}_{0}^{4}$$

where ${\tilde{\nu }}_{0}$ is the excitation laser wavenumber, ${\tilde{\nu }}_{k}$ is the Raman shift of the mode k, and ${\tilde{\nu }}_{0}-{\tilde{\nu }}_{k}$ is the absolute (Stokes) Raman wavenumber. For small Raman shifts, the frequency dependence of the cross-section can be safely approximated as $\sim {\tilde{\nu }}_{0}^{4}$. Equation (24) shows that the same molecule scatters more Raman photons when excited with visible light than when excited with near-infrared light. In particular, the cross-sections at some typical excitation wavelengths, normalized to the cross-section at 514 nm, turn out to be $\frac{\sigma \left(\lambda_{Exc}=633\;nm\right)}{\sigma \left(\lambda_{Exc}=514\;nm\right)}=0.44$, $\frac{\sigma \left(\lambda_{Exc}=785\;nm\right)}{\sigma \left(\lambda_{Exc}=514\;nm\right)}=0.18$, $\frac{\sigma \left(\lambda_{Exc}=1064\;nm\right)}{\sigma \left(\lambda_{Exc}=514\;nm\right)}=0.054$; a factor of 20 in intensity is approximately lost from the visible to the near-infrared.

If the excitation wavelength is resonant with an allowed electronic transition the cross-section can be enhanced by two to three orders of magnitude; this is more likely to occur in the UV-visible region where many organic dyes exhibit an absorption band. As a drawback, short excitation wavelengths and also the resonant effect increase the chance of damaging the sample.

### 4.3. Fluorescence from the Analyte or Contaminants

Raman is a very weak phenomenon, in fact its cross-section is about six to 10 orders of magnitude smaller than the one of fluorescence [6]. Therefore, the fluorescence from the analyte itself or from impurities, even in traces, can overwhelm the Raman signal. In the typical conditions in which a Raman spectrum is collected (shot noise limit) [19], $SNR=\frac{S}{\sqrt{S+B}}$, where S corresponds to the Raman signal itself and B represents the background signal. It is clear that, in absence of background, the best SNR is achieved ($SNR=\sqrt{S}$), while, the higher the background that adds up to the Raman signal, the lower the SNR (SNR→0 for B≫S). In other words, the same Raman peak can be well visible without the background, but on top of an intense background it can be washed out by the high noise level. Hence, a simple subtraction of the background does not work for retrieving a Raman spectrum [19,274]. In SERS, the fluorescence problem is partially mitigated because metals strongly quench fluorescence [108,291,292,293]. Nevertheless, fluorescence can still be an issue, since it can be generated by molecules that are not absorbed on the metallic substrate. For example, if the analyte is dissolved in a solution of nanoparticles, it is likely that part of it will not be absorbed on the nanoparticles. Another example is the drop cast of fluorescent analytes on a substrate formed by islands of metallic structures on glass. In this case, the part of the analyte that is deposited on glass will produce a wide fluorescent background unless, measuring with a microscope, only the metallic islands are selectively illuminated.

Typically, the fluorescence problem is mitigated using excitation lines in the near-infrared (i.e., 785 or 1064 nm), where it is less likely that optical transitions are excited in the analytes, in the impurities or in the materials used as supports to fabricate the SERS substrates. Other recent approaches are the shifted excitation Raman difference spectroscopy (SERDS), the sequentially shifted excitation (SSE), and the subtracted shifted Raman spectroscopy (SSRS) [294,295,296,297,298]. SERDS and SSE are based on the collection of spectra at slightly different excitation wavelengths: Raman bands shift following the excitation wavelength but the (broad) background is left almost unchanged. Mathematical algorithms elaborate the spectra recorded, removing the background. SSRS exploits a similar strategy, but the Raman spectra are shifted by moving the gratings of the spectrograph.

### 4.4. Spectral Sensitivity of the Raman Instrument

Raman photons emitted by the sample must be collected, coupled into a spectrograph, and sent to a detector that converts them into electrical signal. All the components involved in this process, the microscope objective, the optics that drive the Raman signal into the spectrograph (mirrors, lenses, beam splitters, etc.), the spectrograph itself (in particular its gratings), and the CCD possess a significant wavelength dependence. The spectrograph and CCD detector are probably the most critical ones.

**Figure 19:**
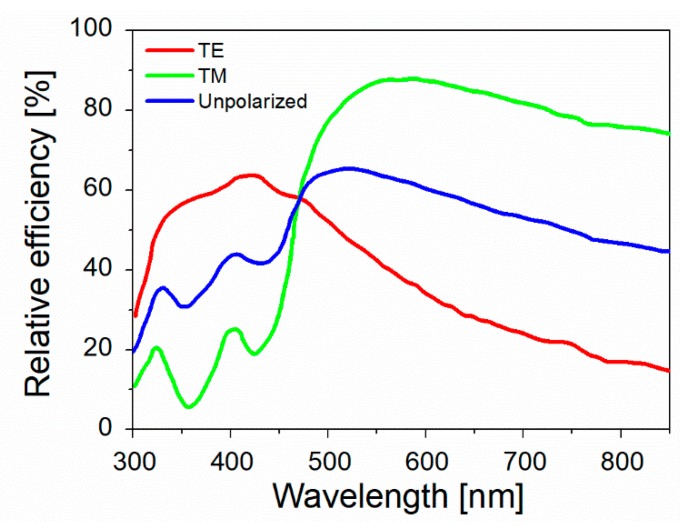
Example of reflectivity for an 1800 groove/mm grating optimized for use between 450 and 850 nm. Transverse electric (TE) indicate that the electric field of the radiation is polarized perpendicular to the plane of incidence; transverse magnetic (TM) indicate that the magnetic field of the radiation is polarized perpendicular to the plane of incidence; unpolarized is the average of TE and TM. Adapted with permission from Adar et al. [299].

The reflectivity of the spectrograph gratings is a complex function of several variables, like the type of metallic coating, the groove density and shape, the polarization of the incident light, and the angle at which it hits the gratings. The reflectivity is often optimized at a certain wavelength (blaze wavelength) and it decreases away from this value [19,299]. Figure 19 shows an example of the relative efficiency with which differently polarized light is reflected by an 1800 groove/mm grating. 

**Figure 20:**
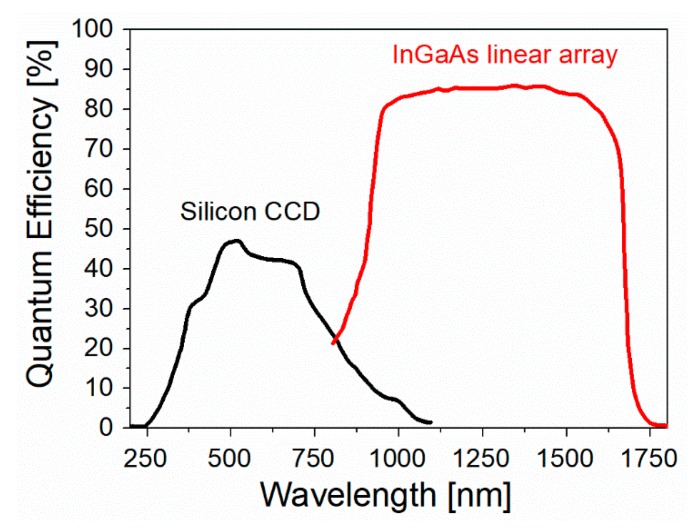
Typical quantum efficiency of a silicon (black line) and of an Indium gallium arsenide (InGaAs, red line) detector: The first corresponds to a Symphony II front illuminated charged coupled device (CCD) and the second one to a Symphony II 1700 InGaAs linear array. Data adapted with permission from Horiba Scientific (https://www.horiba.com).

The quantum efficiency of the detector (the fraction of photons incident on the detector that are converted into photoelectrons) also depends on wavelength. For the visible and the near-infrared region, CCD chips are made of silicon. The quantum efficiency is limited at long wavelengths by the band gap of silicon, which is located at about 1100 nm; above this wavelength, in fact, silicon does not absorb photons and, hence, no photocurrent is generated. For working in spectral ranges above 1100 nm, silicon detectors can be replaced by indium gallium arsenide (InGaAs) detectors. Typical quantum efficiencies of a front illuminated silicon CCD and an InGaAs linear array are reported in Figure 20. 

The spectral response of an instrument, that depends on all the above mentioned components, can be determined by using a source (for example a lamp or a compound) with a known relative emission [19,300,301,302].

### 4.5. Summary

Summarizing, the excitation wavelength influences the intensity of the SERS spectra through several factors, related to the substrate, to the analyte, and to the instrumentation used. In literature, by using different materials and geometrical arrangements, substrates that work from UV to near-infrared have been reported. However, as a rule of thumb, the highest $G_{SERS}$ are found towards the near-infrared. This is because the dielectric constant of typical SERS materials is more favourable (less losses) in this region than in the visible; moreover, the close interaction between nanoparticles, that is the key point in the formation of hot spots, tends to red shift the local field intensity maximum. Concerning the analyte, Raman cross-sections increase at shorter excitation wavelengths, approximately with an ${\tilde{\nu }}_{0}{}^{4}$ dependence. In the visible, it is also more likely that typical analytes have allowed electronic transitions and; therefore, the resonant Raman effect can be exploited. However, a very important drawback is that, when electronic transitions are excited (in the analyte or in impurities), fluorescence can be generated and its presence can strongly reduce the SNR. Concerning the instrument sensitivity, the grating and optics transmittance (or reflection) can be optimized in different spectral regions; however, the quantum efficiency of silicon-based detectors (the most widely employed) drops quite quickly above 800 nm and is limited at about 1100 nm. Therefore, going towards the near-infrared, the instrument sensitivity tends, in general, to decrease. For working at longer wavelengths, the more expansive InGaAs based detectors can be used. In Table 5, the previous considerations are summarized.

With the aim of optimizing the SNR ratio, looking only at $G_{SERS}$ is not enough. In fact, also the instrument sensitivity, the cross-section dependence on the excitation wavelength, and the possible presence of fluorescence backgrounds must be taken into account. In other words, the region in which $G_{SERS}$ is maximum does not necessarily correspond to the region in which the lowest detection limit can be measured. In the last years, there seems to be a tendency to develop instruments able to work at long excitation wavelengths, since the fluorescence is a relevant issue in many applications.

## 5. Fabrication of SERS Substrates

### 5.1. Desired Features of SERS Substrates for Applications

From a practical point of view, SERS substrates should possess several features in addition to those strictly related to the performance. Table 6 summarizes the features of an ideal substrate, as proposed by Natan [85] and Lin et al. [37].

A good substrate will be a compromise among the above-mentioned features. Some applications will preferentially require uniformity/reproducibility (quantitative assays) or enhancement (trace detection of chemicals). It is worth mentioning that, as a general rule, the higher the enhancement, the lower the uniformity/reproducibility of the substrate [6,303]:

(25) $$(\mathrm{Substrate}\;\mathrm{reproducibility})\;\times \;(G_{SERS})\approx \mathrm{constant})$$

**Figure 21:**
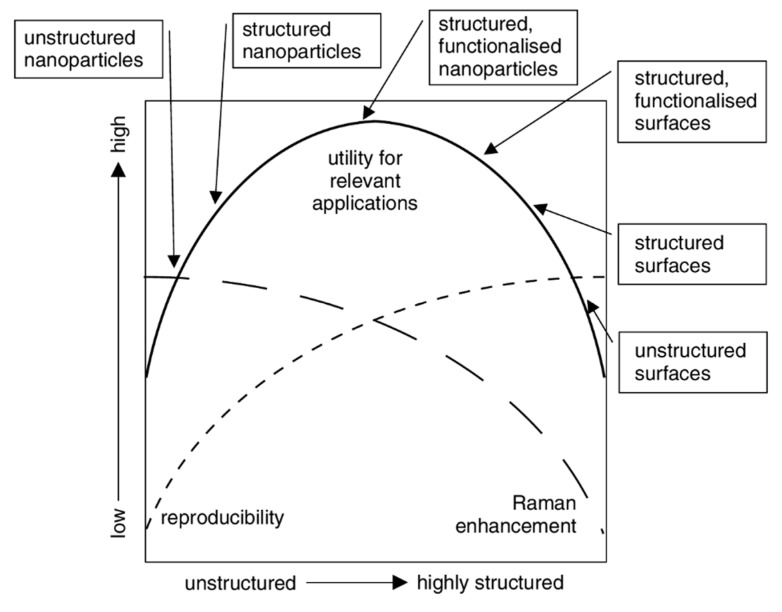
The reproducibility/uniformity and the Raman enhancement for a large number of substrates (Y axis) is correlated to the degree of order (*X* axis). The reproducibility/uniformity (short dashed line) increases with the degree or order of the substrate, while the enhancement (long dashed line) follows the opposite trend. For relevant applications, SERS substrates have to satisfy a tradeoff between the former and the latter. Reproduced with permission from Milton et al. [303]. Copyright (2008), John Wiley and Sons.

The origin of this reciprocal proportion between (Substrate reproducibility) and ($G_{SERS}$) can be figured out on the basis of the following considerations. Large $G_{SERS}$ requires very small gaps, on the order of a few nm. Such gaps can be easily achieved, for example, by aggregating metal nanoparticles, but the price to pay is that the morphology is hard to control in this way; on the other hand, lithographic methods allow one to fabricate structures with a precise and reproducible control of the morphology but the gaps obtainable are significantly larger (normally limited to about 10 nm). Equation (25) has been studied in depth in the review paper by Milton et al. [303]. The authors analyzed a large number of SERS substrates, correlating the degree of order of the substrate (from unstructured to highly structured) with its reproducibility/uniformity (from low to high). The results of this study, summarized in Figure 21, show that reproducibility (short dashed line) increases, whereas the enhancement (long dashed line) diminishes, at higher degree of order of the substrate; vice versa for a lower degree of order. In Section 5.3, some examples of unstructured nanoparticles (aggregated nanoparticles in solution), structured nanoparticles (nanoparticles assembled or grown on solid surfaces), and structures’ surfaces (ordered arrays of nanoparticles) at different degrees of order will be illustrated.

### 5.2. Direct vs. Indirect Detection

**Figure 22:**
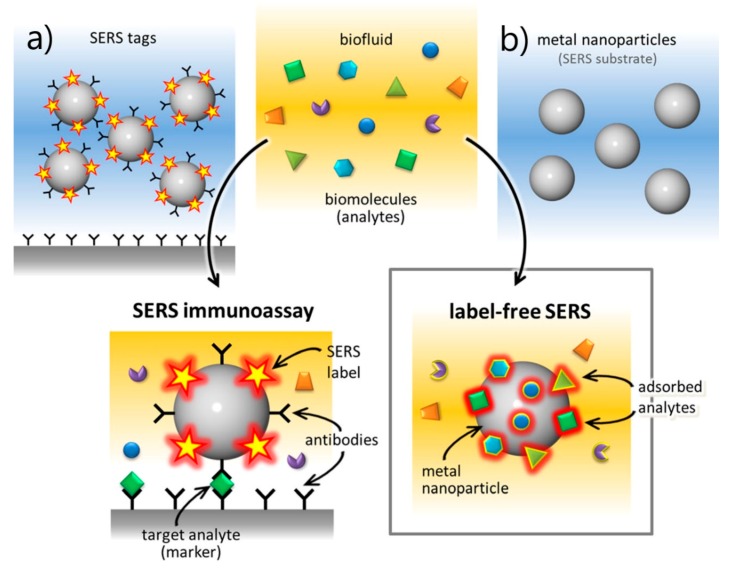
(**a**) Indirect protocol. A SERS tag is functionalized with antibodies and selectively binds to the analyte; its detection is carried out through the spectrum of the Raman reporter contained in the SERS tag. (**b**) Direct protocol. The analyte is adsorbed on the nanoparticle and detected through its own Raman spectrum. Reproduced with permission from Bonifacio et al. [304]. Copyright (2015), Springer-Verlag Berlin Heidelberg.

SERS detection of analytes can be carried out in two conceptually different ways (Figure 22) [38,304,305,306].

Direct (or label free) protocols allow one to identify compounds through their own Raman spectrum and are suitable for species with large cross-sections, typically conjugated organic molecules (i.e., explosives [58,307], contaminants like many pesticides, and food dyes [54,57]). This approach is very straightforward but, on the other hand, it may be difficult to apply in the biological/biomedical field, since biomolecules are characterized by aliphatic bonds and; therefore, possess small cross-sections. Furthermore, they are often immersed in complex matrices that can generate a Raman signal interfering with the one of the biomolecules themselves.

Indirect protocols can overcome the above-mentioned shortcomings (at the price of a more complex detection scheme), resorting to the use of SERS labels (also called tags). SERS labels are complex species containing an efficient Raman reporter and engineered to selectively bind to the molecule of interest. In this case, the analyte is detected through the spectrum of the Raman reporter.

In the following sections, some fabrication methods for SERS substrates will be illustrated, with more emphasis on scalable and cost-effective procedures.

### 5.3. Substrates for Direct Detection

In the following, several types of substrates (aggregated nanoparticles in solution, nanoparticles assembled on a surface and ordered arrays of nanoparticles) will be presented; they are summarized in Table 7.

#### 5.3.1. Aggregated Nanoparticles in Solution (Unstructured Nanoparticles)

Spherical silver and gold colloids are the most widely used nanoparticles for SERS experiments in solution. They are often synthesized by reduction of a precursor salt with sodium citrate in water; the citrate adsorbed on the surface of the nanoparticles acts also as an electrostatic stabilizer [322,323]. The localized surface plasmon resonance typically peaks at approximately 400 and 520 nm for silver and gold, respectively. Spherical nanoparticles can also be fabricated by laser ablation [324,325]. In this case, a metal target is placed at the bottom of a solution and a pulsed laser is nearly focused at its surface; the heating and photoionization processes cause the metal to change state of aggregation, forming liquid drops, vapors, or a plasma plume. The atomized material from the target then condenses, leading to the formation of nanoparticles [324,325]. With laser ablation, nanoparticles can be fabricated from different materials, choosing the appropriate target plate [324], and also without capping agents. The absence of capping agents allows an easier functionalization of the nanoparticles. In addition to spherical nanoparticles, several different shapes have been developed in order to cover a broader spectral range and to improve their SERS enhancing properties [153]. The synthetic methods for several types of nanoparticles (spheres, rods, cubes, pyramids, plates, wires, corals, stars, etc.) and their SERS applications are reported in papers dedicated to this subject [39,40,308,309,310,311,312,313,314,315,316,317,318,319,320,321,322,323].

A very efficient method for increasing the SERS signal relies on the aggregation of the nanoparticles. For example, it can be carried out by adding a salt (i.e., NaCl, NaNO_3_, etc.) to the solution. The consequent increase in the ionic strength of the solution reduces the screening of the stabilizing charges at the surface of the nanoparticles, inducing aggregation [368,369]. The analyte itself may play the same role if it is an ionic dye or if, owing to its functional groups, it displaces the stabilizing ligands at the surface of the nanoparticles (for example pyridine). Methods to improve the repeatability of SERS based on the aggregation of colloidal nanoparticles have been proposed in some papers. For example, Tantra et al. [370] studied different procedures for the SERS determination of Rhodamine 6G with aggregated silver colloids; in particular they studied the effect of the filtration (to make the nanoparticle more monodispersed), of the vortex time during the aggregation step, and of the storage conditions over a period of six months. They found out that a longer mixing time was associated to a better reproducibility of the SERS data, probably due to a reduced formation of random aggregates, while filtration and storage conditions had only a small effect. Meyer et al. [371] showed that a silver colloid, under suitable conditions, can be aggregated by addition of KCl, forming a long-living metastable state that does not lead to the precipitation of large clusters. Colloids in this metastable state were able to generate intense SERS signal from test dyes, with obvious advantages in terms of stability of the signal and repeatability. Molecular linkers can be used to form dimers with a very small gap, corresponding to the size of the linker itself. To this aim, bi-functional molecules, like 4,40-diaminoazobenzene [326] or complementary DNA strands [327], have been used. In this case, the analyte of interest should be a moiety of the linker, or it should be somehow encoded in the nanoparticles. The review by Guerrini et al. [328] provides an account of molecularly mediated methods for assembling plasmonic nanoparticles. Another elegant possibility is to make use of contactless manipulation methods, like laser tweezers. This technique exploits the optical forces to which metal nanoparticles are subjected when illuminated by strongly focused laser beams. In qualitative terms, a nanoparticle is subjected to two forces: (1) A gradient force that is attractive towards the high intensity region of the laser beam if the excitation wavelength is longer than the surface plasmon resonance of the nanoparticle, and repulsive in the opposite case; (2) a radiation pressure force that propels the nanoparticle along the propagation direction of the beam [329,372]. A proper choice of the experimental parameters allows one to control the aggregation process. Laser tweezers have been used by Foti et al. [329] to push and aggregate gold nanorod–biomolecule complexes in 5–10 µm spots at the bottom of a glass microcell.

The use of aggregated nanoparticles in solution is a very practical method for SERS detection, which exploits easily synthesizable (or even commercial) materials and, under suitable conditions, provides very strong enhancements. A drawback of this method is that the aggregation process is difficult to control [328] and often leads to non-reproducible results, making the implementation of quantitative analysis more difficult.

#### 5.3.2. Nanoparticles Assembled on a Surface (Structured Nanoparticles)

*Electrochemical roughening and electrochemical deposition*

Electrochemical techniques are versatile methods for the deposition of metal nanostructures, since the experimental conditions are easy to tune, enabling the creation of a wide range of nanoelectrodes with different sizes, shapes, and distributions. There are two main approaches for the electrochemical preparation of nanostructured samples with high surface area: he electrochemical roughening (ER) and the electrochemical deposition (ED). Both of them can be accomplished in a two or three-electrode cell system, depending on whether the process is carried out under galvanostatic (constant current) [330] or potentiostatic condition (constant potential) [373], the latter being the most common approach. The electrochemical setup consists in a working electrode, where the ER or ED take place, and it is necessarily made of a good electronic conductor of the metal to be roughened in the case of ER or of a foreign conductive substrate such as glassy carbon, ITO, or other metals in the case of ED. A reference electrode is necessary for the precise application or variation of the potential at the working electrode while a counter electrode made of platinum or graphite allows one to close the amperometric circuit. Basically, all the metals in the periodic table can be nanostructured in different shapes (i.e., spherical, cubical, dendrite, and fractal-like) by ER or ED, even though the most commonly used are silver, gold, and copper [165,374]. Briefly, ER consists of the application of very short pulses (20 ms) at very positive potential to prompt the dissolution of small areas of metal from the initially smooth pristine surface, followed by a second pulse at a more negative potential to induce the random redeposit of the metal back onto the electrode. ED needs the careful preparation of a deposition bath, which must contain the salt of the metal to be deposited, an inert supporting electrolyte for sustaining and buffering the ionic conductivity and possibly organic or inorganic ligands that can be purposely added for controlling the shape and the dimension of the metal particles. A special attention must be put in the type of reference electrode to be used, since the most common Hg|Hg_2_Cl_2_|Cl^−^ (SCE) or Ag|AgCl|Cl^−^ electrodes involve the unwanted percolation of Cl^−^ inside the deposition bath, where halide ions can drive and modify the shape of metal nanoparticles, especially in the case of silver and copper [375]. For this reason, the employment of a salt bridge, which avoids any contamination of the working electrode or of a Hg|Hg_2_SO_4_|K_2_SO_4_ reference electrode, is advisable [254].

**Figure 23:**
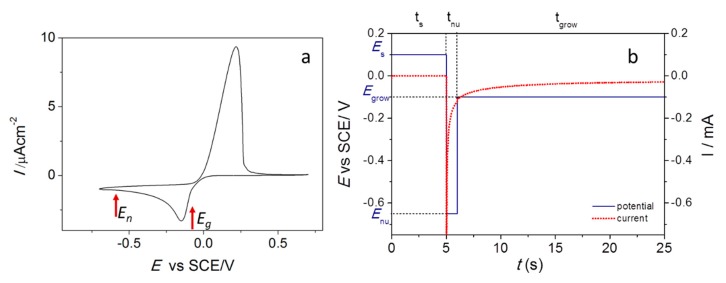
(**a**) Cyclic voltammetry in H_2_O + 5 mM CuSO_4_ + 0.1 M LiClO_4_ at glassy carbon, scan rate 0.2 V·s^−1^; (**b**) double potential pulse for the Cu deposition applied in this study. Adapted with permission from Durante et al. [254]. Copyright (2014), John Wiley and Sons.

The potential controlled deposition of metal nanostructures can be accomplished in several ways, for example, by applying a constant potential, a potential variable in time such as in cyclic voltammetry (CV) [376], or a series of potential steps (double-step potential deposition, DSPD) [377]. DSPD allows a better control over the dimension and dispersion of metal nanoparticles, since it is composed of independent nucleation and growing steps, which can be identified by considering the reduction potential of the metal salt in a suitable electrolyte, determined by cyclic voltammetry (Figure 23). Figure 23a reports the cyclic voltammetry response of CuSO_4_ in 0.1 M LiClO_4_ electrolyte. The CV shows a cathodic peak at *E*_p_ = −0.150 V vs. SCE, responsible for the reduction of Cu^2+^ to metal copper, and an oxidation peak in the reverse scan at *E*_p_ = 0.215 V vs. SCE, where the dissolution of copper stuck on the electrode occurs. The nucleation step (*E*_n_) is set at a sufficiently negative potential between the metal reduction peak and the hydrogen curve discharge and, as a consequence, instant nucleation of the metal takes place. In this potential range, the deposition process is controlled only by diffusion, while secondary processes, such as hydrogen evolution and bubbling, which can induce the detachment of metal nanoparticles, can be avoided. The growth step potential (*E*_g_) is set between the peak onset and the peak potential, so that the process is kinetic-controlled and the metal nanoparticles grow without further nucleation of new sites. Both the *E*_g_ value and the length of the growth step allow one to control the dimension of the metal nanoparticles [254]; furthermore, a third stripping step can be included for resizing overgrown particles or for dissolving small and unstable particles, resulting in an overall less dispersed nanoparticle size distribution. A pre-conditioning step, at positive and/or negative potentials, is also advisable before the two deposition steps for stripping off metal traces and for desorbing anions or impurities from the support surface, respectively [378]. It is worth noting that the co-deposition or the sequential deposition of different metals can be easily accomplished by adding a second metal salt in the deposition bath and by carefully choosing the *E*_n_ and *E*_g_ potentials.

Other parameters that can produce significant modifications in the shape and dimension of the electrogenerated nanoparticles are the concentration of the metal salt precursor [379], the type of working electrode [380], the concentration and the nature of the supporting electrolyte [381], the type of solvent [382,383], and the presence of inorganic or organic ligands. The ligand assisted electrodeposition is of particular interest because ligands produce a change in the reduction potential of the resulting complex, and precisely the reduction potential shifts to more negative values as the stability constant between the metal cation and the ligand increases. By way of example, the reduction potential of AgL (L = ethylenediaminetetraacetic acid (EDTA), ethylenediamine and CN^−^) shifts from the value of 0.33 V vs. Ag/AgCl/Cl^−^ for Ag^+^ to 0.28, −0.03, and −0.77 V, respectively, being the AgCN complex formation constant the highest one [376]. It is worth noting that weak ligands (EDTA, 10–90 nm; ethylenediamine, 10–90 nm) bring about the electrodeposition of bigger nanoparticles compared to strong ligands; very strong ligands, such as cyanides, may cause the complete redissolution of Ag nanoparticles leading to the formation of dissolved complexes like dicyanoargentate [Ag(CN)_2_]^−^ [384]. When the dissolution and re-deposition pulses are cyclically repeated thousands of times, the formation of nanostructured features can be obtained.

ER and ED produce large area substrates, with good enhancement; this method is also suitable for a reproducible large scale fabrication [37,330].

*Nanoparticles adsorbed on solid surfaces*

A very well-known method for assembling a bi-dimensional array of spherical nanoparticles on a surface was proposed by Natan et al. [42,331,332] in the ’90s. Typically, a glass surface is silanized with (3-Aminopropyl)trimethoxysilane (APTMS). The methoxy groups of APTMS are displaced by the –OH groups on the surface, leading to the formation of a covalent –Si–O–Si– bond [385]; the terminal –NH_2_ group of APTMS is left exposed to the liquid phase. This substrate is then immersed in a solution containing gold or silver nanoparticles in water, that self-assemble on the surface forming a monolayer. UV-Vis spectroscopy shows that, during the adsorption process, aggregates are formed, as evidenced by the appearance of a broad band around 650–700 nm. The strong interaction between the pending –NH_2_ groups and the nanoparticles is important in preventing the spontaneous coalescence that would instead occur if nanoparticles were simply drop cast on glass. In this way, an array of closely spaced, but physically separated, objects with optimal SERS properties is formed [42]. The controlled immobilization of nanoparticles on a surface improves the reproducibility of a substrate, with respect to the simple drop cast procedure [36]. The grafting of the nanoparticles is irreversible and the interfering SERS signal from APTMS is very weak. This method is very flexible since several types of nanoparticles (with different size, shape, and composition) can be assembled on the surface; moreover, the supporting substrate can be not only glass, but also, for example, silicon, plasma-treated Teflon, and ITO [331]. Large surfaces, in the order of cm^2^, can be produced, with good enhancement and reproducibility [42,331,333].

*Nanoparticles on flexible substrates*

A relatively recent and interesting possibility is to deposit nanoparticles on paper [41,334,386]. Several methods can be exploited to this aim, for example, drop casting [387], dip-coating [388,389,390,391], ink-jet [334,335,336,337], and screen [338,339] printing, pen on paper [340], and physical vapor deposition [392].

**Figure 24:**
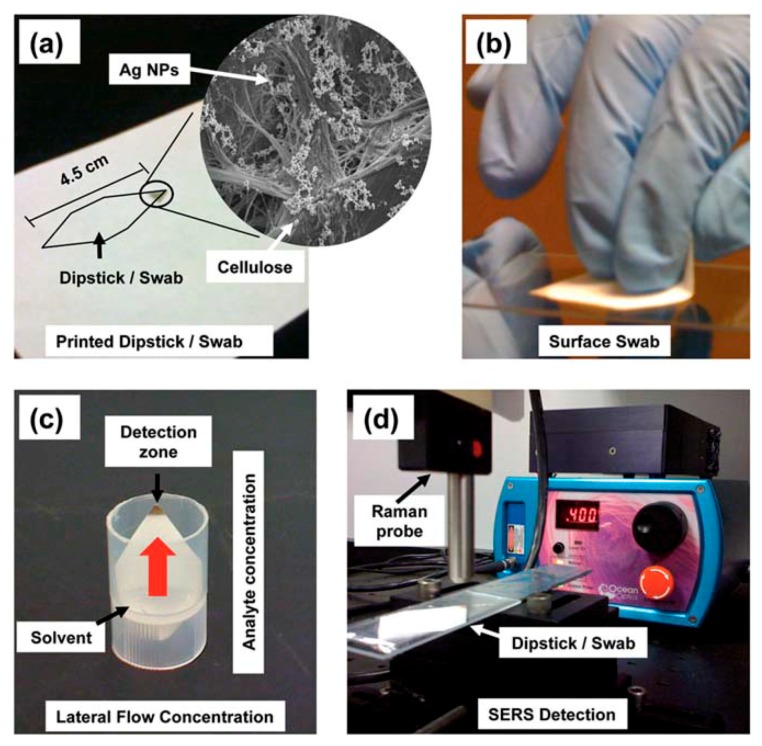
(**a**) Dipstick/swab paper substrate: The SERS active region is printed in the top vertex; the inset is a SEM image showing the silver nanoparticles at the surface of cellulose fibers. (**b**) Substrate used as a swab to collect the analyte. (**c**) The swab/dipstick impregnated with the analyte is immersed in a solvent. The solvent flows through the paper substrate, wicked by capillary forces, and concentrates the analyte in the SERS active region. (**d**) Instrument used for collecting SERS spectra. Reproduced with permission from Yu et al. [393]. Copyright (2013), the Royal Society of Chemistry.

**Figure 25:**
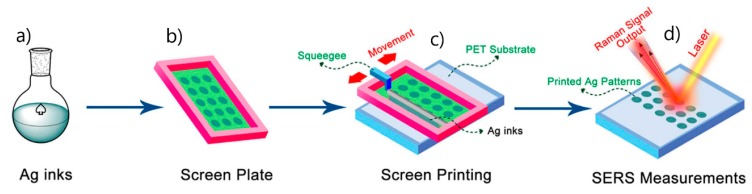
Illustration of the screen-printing process. The nanoparticle ink (**a**) and a screen plate (**b**) are used to print an array of SERS active areas with the help of a squeegee (**c**); SERS measurements are carried out on the printed spots (**d**). Reproduced with permission from Wu et al. [338] under Creative Commons 4.0 license (https://creativecommons.org/licenses/by/4.0/).

The ink-jet method is explained in detail, highlighting the critical issues, in a work by White et al. [335]. The authors used a commercial ink-jet printer to print silver nanoparticle spots on chromatography paper. The key points in this fabrication process were: (a) The choice of the paper substrate. Filter paper and chromatography paper (but not for example printer paper, coffee filter paper, and cotton fiber paper) exhibited a reasonably low background Raman signal. (b) The hydrophobization of the paper. This operation was carried out by printing a sizing agent (hexadecenyl succinic anhydride) over the whole paper surface, followed by heating. This operation prevented the spreading of the ink and analyte drops that would have occurred on the (non-treated) hydrophilic paper surface. (c) The fabrication of the ink. Silver nanoparticles were synthesized with the standard Lee et al. procedure [323] in water and concentrated by centrifugation; the viscosity and the surface tension of the solution were adjusted by re-dispersing the nanoparticles in a water/glycerol mixture. Refillable printing cartridges were filled with this ink. (d) The printing processes. It was carried out several times on the same spots in order to increase the concentration of the nanoparticles. The same authors also introduced the concept of lateral flow concentration [336,393], illustrated in Figure 24. The SERS substrate is a rhomboid-shaped piece of paper with the SERS active region printed in the top vertex (Figure 24a). It can be used as a dipstick (a solution containing the analyte is drop cast on the non-SERS area) or as a swab (Figure 24b); when the dipstick/swab substrate is immersed in a solvent (Figure 24c), the flow of the solvent, wicked by capillary forces, concentrates the analyte in the SERS region (Figure 24c). The apparatus for SERS measurements is illustrated in Figure 24d. Screen printing makes use of a screen plate with a designed pattern; the ink is transferred to the substrate through the apertures in the screen by means of a squeegee that is moved across the screen plate (Figure 25). The main issues involved with this method are analogous to the points (a–c) discussed above. This procedure has been used by Qu et al. [339] and by Wu et al. [338] to print a silver nanoparticle array on paper and on polyethylene terephthalate, respectively.

Polavarapu et al. [340] proposed the pen on paper approach. This method makes use of a fountain pen, that has been filled with a nanoparticle ink with the proper concentration and viscosity, to write a SERS active pattern on paper.

Advantages of this type of substrates are their simplicity, low cost, and the possibility to be fabricated to the need, circumventing the problem of the shelf life stability. The lateral flow concentration is a very practical and cheap way of concentrating the analyte, if compared to more sophisticated methods like microfluidics. The possibility of using paper substrates as swabs is an interesting feature for determining the presence of an analyte, although with this sampling method is not so easy to carry out a quantitative analysis.

**Figure 26:**
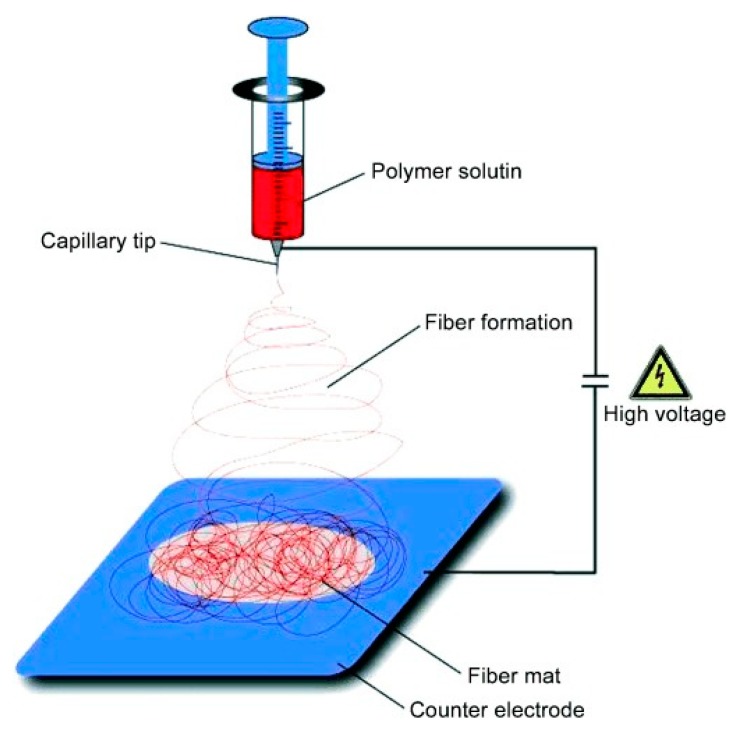
Illustration of the electrospinning process. Reproduced with permission from Greiner et al. [394]. Copyright (2007), John Wiley and Sons.

**Figure 27:**
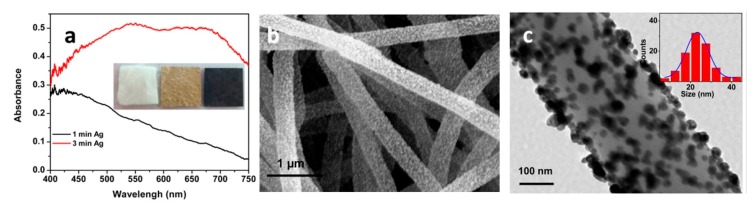
(**a**) Reflectance spectra of the nanofibers decorated with silver nanoparticles after 1- and 3-min immersion in the Tollen’s reactive. In the inset, from left to right: Macroscopic images of the polyacrylonitrile (PAN) fibers, bare, functionalized with amidoxime, and functionalized with silver nanoparticles. (**b**) Representative SEM image of the fibers after the electroless plating step. (**c**) Representative transmission electron microscopy (TEM) image of the fibers after the electroless plating step. In the inset, size distribution of the nanoparticles. Reproduced with permission from Zhang et al. [341]. Copyright (2012), American Chemical Society.

Polymeric fibers are another possibility for depositing or embedding metallic nanoparticles. Long polymeric fibers, with typical diameters from tens to hundreds of nm, can be produced by electrospinning [394]. This method is illustrated in Figure 26. A polymer solution is pumped through a thin nozzle; the high electric field difference that is applied between the nozzle (that also works as an electrode) and a counter electrode, causes the “extraction” of the polymer solution that dries out before reaching the counter electrode, forming the fibers. Zhang et al. [341] produced polyacrylonitrile (PAN) fibers; they were subsequently reacted with hydroxylamine in order to expose amidoxime groups (–C(NH_2_)=N–OH) on the surface. Palladium seeds were then grown at the nanofiber surface by reduction of PdCl_2_ with SnCl_4_ and, afterwards, silver nanoparticles were deposited by electroless plating with the Tollen’s reagent. The growth of the silver nanoparticles was optimized on the SERS signal of a test analyte, by varying the stirring conditions and the immersion time in the electroless deposition step. Figure 27 illustrates this type of substrates. He et al. [343] synthesized silver nanoparticles by microwave irradiation and aggregated them introducing polyvinyl alcohol (PVA); fibers electrospun from this solution were formed by nanoparticle aggregates dispersed in the PVA matrix. Yang et al. [342] fabricated nanofibers starting from a solution containing silver nitrate, agar, and PAN; by exposing the samples to UV light, silver ions were photoreduced, leading to the formation of silver nanoparticles at the surface of the nanofibers.

Overall, electrospun nanofiber mats are large area and economic substrates, exhibiting good enhancements. Similarly to paper substrates, they are flexible and could be used as swabs; the fabrication method is suitable for mass production.

*Laser direct writing*

**Figure 28:**
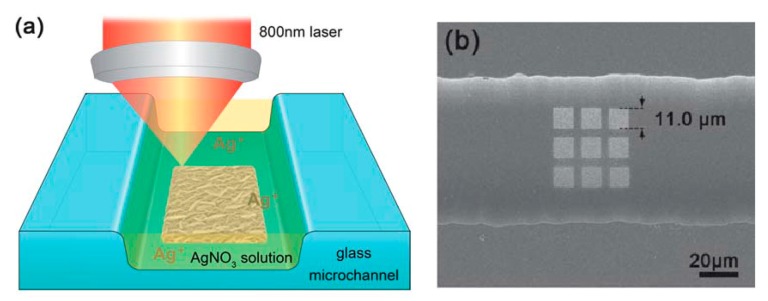
(**a**) Illustration of the laser writing method used to fabricate SERS substrates in microfluidic circuits; (**b**) SEM image of the SERS substrates integrated in the microfluidic channel. Reproduced with permission from Xu et al. [345]. Copyright (2011), the Royal Society of Chemistry.

Metal nanoparticles on a surface can be synthesized by photoreducing a silver or gold precursor. Lee et al. [344] deposited a solution containing HAuCl_4_, polyvinylpyrrolidone (PVP) and ethylene glycol on a glass surface functionalized with APTMS. The photo reduction was carried out by exposing the sample to a strongly focused femtosecond laser and scanning it in order to produce the desired pattern (Nanoscribe commercial machine, https://www.nanoscribe.de/en/). This allowed the fabrication of microstructures, internally formed by aggregated quasi-spherical gold nanoparticles whose size was controllable by varying the PVP concentration. Ethylene glycol promoted the reduction of Au^3+^ through the polyol reduction reaction; the functionalization of glass with APTMS turned out to be important to firmly anchor the metallic structure to the glass. The authors also showed that such structures, written inside a microfluidic circuit, could be used for the detection of gaseous analytes. Xu et al. [345] adopted an analogous procedure to write a SERS active silver pattern in a microfluidic circuit, starting from a solution containing AgNO_3_, trisodium citrate, and ammonia. Figure 28a illustrates the laser direct writing method and Figure 28b shows an example of SERS substrate patterned into a microfluidic circuit.

The main advantage of this method is clearly the possibility of creating SERS active microstructures of the desired shape and, where needed, making laser direct writing very suitable for combination with microfluidics.

*Nanowires for remote SERS sensing*

**Figure 29:**
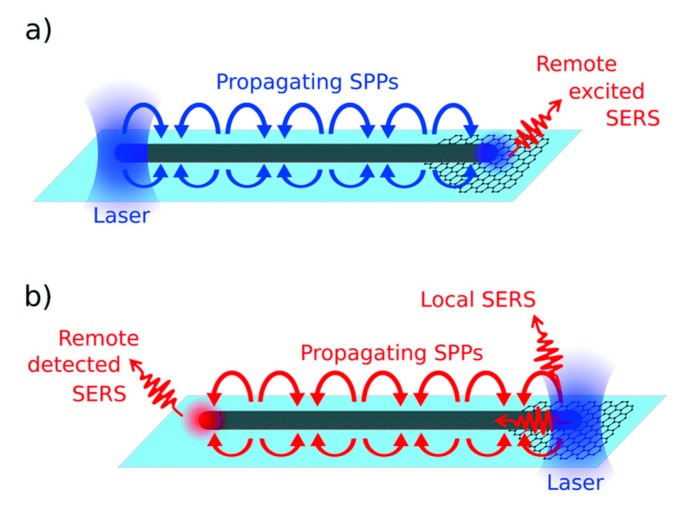
(**a**) Remote SERS excitation; (**b**) remote SERS detection. A commercial silver nanowire works as a waveguide and a graphene sheet is used for generating the SERS signal. Reproduced with permission from Coca-López et al. [396]. Copyright (2018) the Royal Society of Chemistry.

Metallic nanowires can work as plasmonic waveguides: The surface plasmon excited at one end can propagate along the wire and excite the molecules placed up to tens of micrometers away from the excitation point. Potential advantages of this method are a reduction of the background and the possibility of using the nanowires as “needles” to probe the inner part of living cells [395]. Coca-López et al. [396] prepared a sample by depositing graphene sheets on a glass slide and drop casting a solution of commercial silver nanowires on top; for the optical experiments only those wires that were touching graphene with only one tip were selected. The authors demonstrated both the remote SERS excitation (the laser excites the surface plasmon on the tip not in contact with graphene and its SERS signal is detected from the other tip, Figure 29a) and remote detection (laser excites the plasmon on the tip in touch with graphene and its SERS signal is detected from the other tip, Figure 29b). Conceptually similar experiments were carried out by Fang et al. [397] on silver nanowires (synthesized by direct current electrodeposition in porous anodic alumina) coupled to spherical nanoparticles and by Hutchison et al. [398] on nanowires synthesized using a wet chemistry protocol.

#### 5.3.3. Ordered Arrays of Nanoparticles (Structured Surfaces)

*Anodic alumina template*

**Figure 30:**
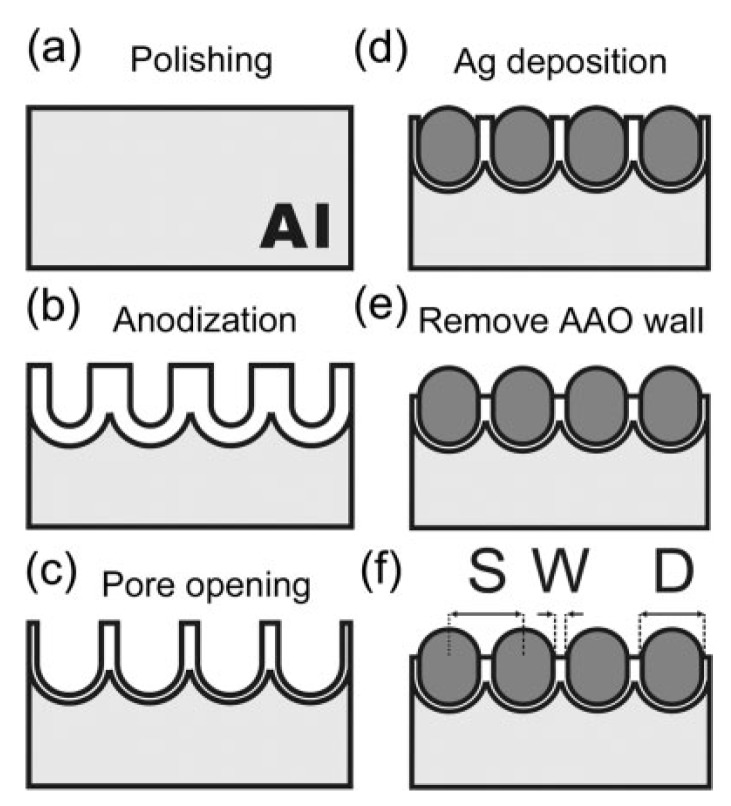
Steps involved in the fabrication of a hexagonal array of metallic nanopillars with the anodic alumina template method. (**a**) An aluminum foil is polished; (**b**) An array of vertically aligned nanopores is produced by anodization; (**c**) Pores can be widened by etching with a phosphoric acid solution in order to tune the wall thickness and hence the gap size in the final structure; (**d**) Silver is electrodeposited in the pores forming nanopillars of controlled hight; (**e**) Alumina is partially etched to expose the silver nanopillars; (**f**) The final array is characterized by interparticle distance S, interparticle gap W and nanopillar diameter D. Reproduced with permission from Wang et al. [402]. Copyright (2006), John Wiley and Sons.

The template assisted electrochemical deposition (TAED) allows the deposition of metals with controlled geometry and disposition by using inorganic ordered templates, such as silica [399] and alumina [400], or polymeric template polycarbonate membranes or polystyrene microspheres [36,401]. Polymeric membranes or microspheres are commercially available as well as ordered alumina membranes which can, nevertheless, be easily prepared by anodization of an aluminum foil or of a thin aluminum film deposited by sputtering over a conducting material. The anodization causes the formation of channel arrays with a high aspect ratio and regular pore arrangements via self-organization [400,402]. In the case of silica, the template is grown by the application of a suitable cathodic potential to an electrode immersed in a surfactant-containing hydrolyzed solution, to generate the hydroxyl ions that are necessary to catalyze polycondensation of the precursors and self-assembly of hexagonally-packed one-dimensional channels that grow perpendicularly to the electrode surface [399]. Notwithstanding the type of template, metal deposition is carried out in galvanostatic conditions by both constant or alternating current deposition, after which the template is removed leaving free standing metal nanostructures [347]. The fabrication process of an array of nanorods is summarized in Figure 30. The gaps between the pillars is a key parameter for SERS, since, in the realistic case in which the laser illuminates the sample from top, the light is polarized perpendicularly to the pillars and; therefore, the hot spots are generated in between them. This gap can be tuned in the range 5 to 25 nm [402]. Several authors used this fabrication method to prepare SERS substrates with good enhancements, for example, Das et al. [346], Giallongo et al. [347], Toccafondi et al. [348], and Marinaro et al. [349], with good enhancements.

**Figure 31:**
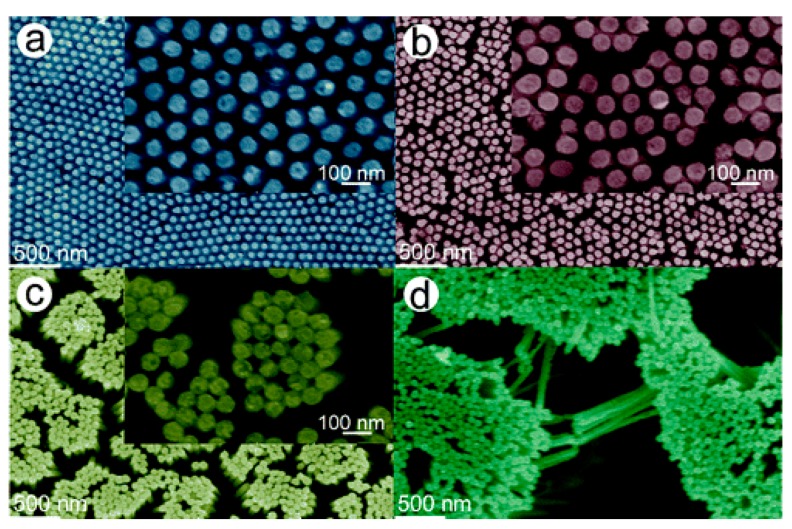
SEM images of the sample after (partial) dissolution of the alumina template with NaOH at different etching times: 0 s (**a**), 210 s (**b**), 270 s (**c**), and 450 s (**d**). The controlled etching of the template makes the nanopillars collapse on each other, generating tip–tip hot spots. Reproduced with permission from Lee et al. [350]. Copyright (2006), the American Chemical Society.

**Figure 32:**
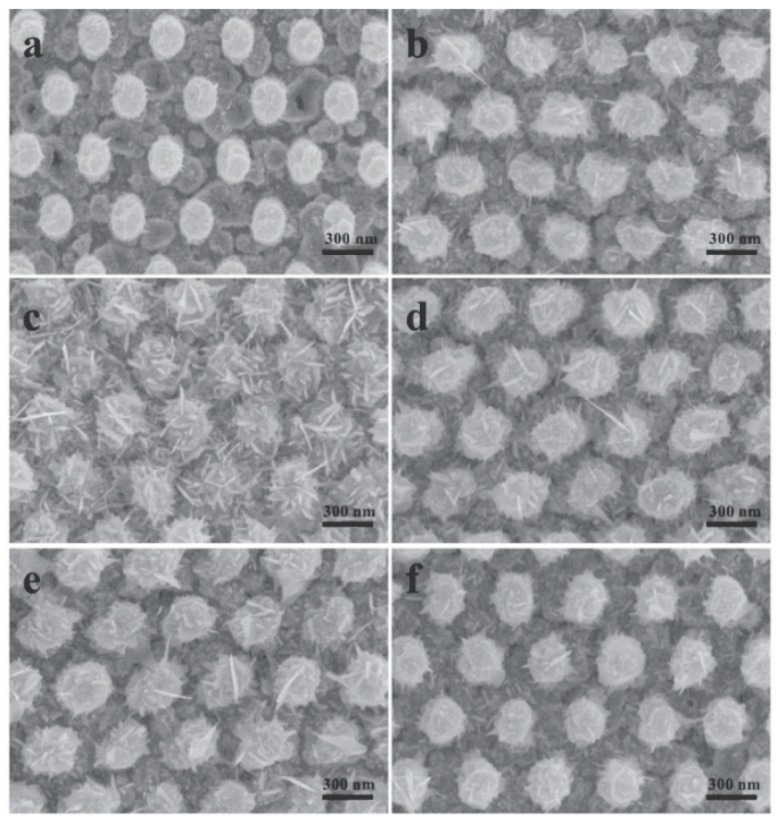
SEM images of the gold nanochestnuts grown at the top of the nanopillars by galvanic displacement at different reaction times: 10 min (**a**), 15 min (**b**), 20 min (**c**), 30 min (**d**), 45 min (**e**), and 65 min (**f**). Reproduced with permission from Geng et al. [351]. Copyright (2018), IOP Publishing Ltd.

It is worth mentioning that, in addition to optimize the aspect ratio of the pillars and the gaps among them, other strategies have been proposed to improve the SERS signal. Lee et al. [350] demonstrated that a controlled etching of the alumina template leads to a tilting of the metallic pillars that, in turn, causes the formation of hot spots when two or more tips come into contact. The authors showed that, if the sample was first functionalized with the molecule and then etched (the analyte is present only on the tips), the SERS signal increased with the etching time due to the formation of tip–tip hot spots. In Figure 31, SEM images at increasing etching times are shown. Geng et al. [351] fabricated an array of nickel nanopillars with a thin layer of silver on top; the latter served as a sacrificial layer for the reduction of a gold precursor (HAuCl_4_) that lead to the formation of a spiky (chestnut-like) structure on top of the nanopillars. The SERS signal was optimized as a function of the growing time of the chestnut-like structures. Representative SEM images are reported in Figure 32.

*Electron beam lithography*

**Figure 33:**
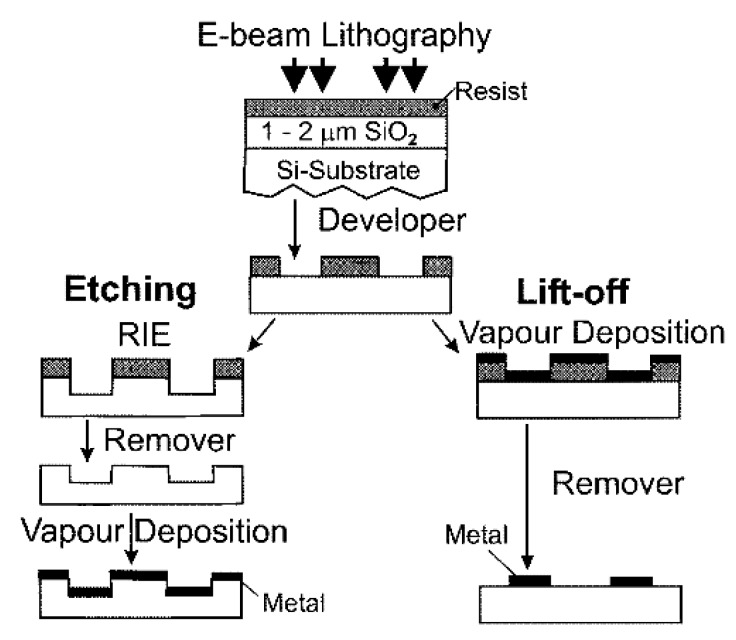
Steps involved in the electron beam lithography (EBL) fabrication. Reproduced with permission from Kahl et al. [404]. Copyright (1998), Elsevier Science S.A.

The process of electron beam lithography (EBL) [403,404,405,406] is illustrated in Figure 33. In the first step, the desired pattern is drawn by a focused electron beam on an electron-sensitive polymer, called resist. The exposure to the electron beam modifies the solubility of the resist; in particular, positive resists become soluble after electron exposure and negative resists become insoluble after electron exposure. In the second step, the sample is developed by immersing it in a solvent that removes the soluble portion of the resist, generating the desired pattern. Etching or lift off methods can now be used to create a metallic pattern (left- and right-hand side in Figure 33). Reactive ion etching (RIE) can be used to write the polymer pattern into the substrate (the substrate is etched, but the polymer is not), then the polymer is removed and the metal evaporated; in this case the whole surface is covered with metal. Alternatively, the metal can be deposited straight after the development stage, followed by removal of the polymer (lift-off); in this case, metal islands are formed.

**Figure 34:**
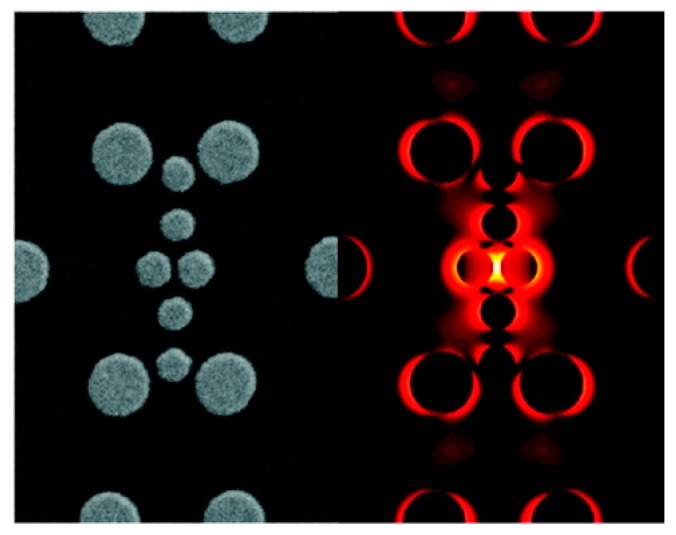
Genetically optimized array of nanoparticles: SEM image (left) and field localization (right). Reproduced with permission from Forestiere et al. [44]. Copyright (2012), the American Chemical Society.

**Figure 35:**
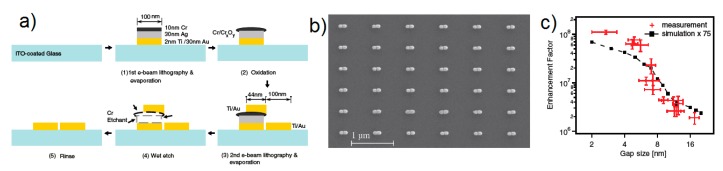
(**a**) Fabrication process for sub 10 nm gaps; (**b**) SEM image of the array of dimers fabricated; (**c**) experimental and theoretical SERS enhancement as a function of the gap. Reproduced with permission from Zhu et al. [43]. Copyright (2011), John Wiley and Sons.

Forestiere et al. [44] exploited a theoretical (genetic) algorithm to optimize the SERS response of an ensemble of nanoparticles on a bi-dimensional surface as a function of the their relative position and of their radius. They showed that the genetically engineered substrates exhibited an order of magnitude larger enhancement compared to a reference dimer. Figure 34 shows a SEM image of the optimized array and the local field distribution. Chu et al. [354] engineered and fabricated a substrate whose enhancement was optimized both for the laser and for the Raman frequencies; therefore maximizing the product that defines the electromagnetic enhancement $G_{SERS}^{Em}\left(\left|E^{4}\right|\right)={\left[\frac{E_{Loc}\left(\omega_{L}\right)}{E\left(\omega_{L}\right)}\right]}^{2}{\left[\frac{E_{Loc}\left(\omega_{R}\right)}{E\left(\omega_{R}\right)}\right]}^{2}$ (Section 2.1.3). Yan et al. [45] fabricated an array of PMMA nanowells that worked as a template for the electrostatic assembly of gold nanoparticles at the bottom of the nanowells themselves; after etching the PMMA, an ordered array of clustered nanoparticles was produced. This approach combined the advantage of lithographic techniques (the long-range order) with the advantage of the aggregated nanoparticles (the high signal due to the formation of small gaps). Zhu et al. [43] used a clever strategy for fabricating sub 10 nm gaps. In the first round of EBL fabrication and metal evaporation, an array of isolated multilayer gold/silver/chromium parallelepipeds was prepared. Afterwards, chromium was oxidized to produce a controlled later swelling of the metal. A second round of EBL fabrication and metal evaporation was used to form nanoparticle dimers, whose gap was determined by the lateral expansion of the chromium. This process is illustrated in Figure 35a and a representative SEM image of the sample is reported in Figure 35b. SERS measurements and simulations (Figure 35c) demonstrated a two-order of magnitude increase in the enhancement upon reduction of the gap, from 16 to 2 nm.

**Figure 36:**
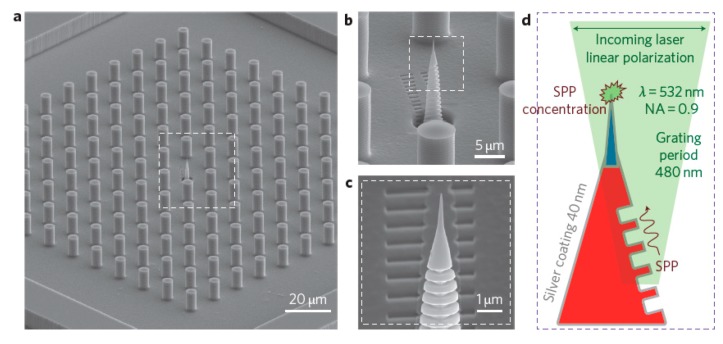
(**a**) SEM image of the super-hydrophobic structure with the nanotip in the center; when a drop of solution is deposited on this device, the high contact angle between the super-hydrophobic structure and the drop causes the analyte to concentrate on the nanotip during the evaporation process; (**b**,**c**) detailed SEM images of the nanotip; (**d**) a laser illuminates the nanotip generating a surface plasmon that propagates upwards and concentrates at the top of the nanotip. The large electromagnetic field produced allows the SERS detection of the analyte. Reproduced with permission from De Angelis et al. [352], (2011) Macmillan Publishers Limited.

De Angelis et al. [352] fabricated a device formed by a super-hydrophobic surface with a specially designed nanotip at the center (Figure 36). When a drop of solution containing an analyte at very low concentration was deposited on the surface; it dried, concentrating the analyte on the nanotip. The illumination of this device with a laser generated a propagating plasmon that concentrated the electric field exactly where the analyte was deposited. This is; therefore, an example of SERS substrate based on propagating plasmons.

Other geometries fabricated by EBL and used for SERS experiments are nanoslits [407], elongated nanoparticles [27], and metallic tapered waveguides for the adiabatic compression of propagating surface plasmons [353].

EBL allows to finely control the geometry of the substrate with a high degree of reproducibility; therefore, it is ideally suited for fabricating substrates whose properties have been theoretically engineered in order to optimize the enhancement and to carry out structure-property studies. The best resolution is normally around 10–20 nm [403], which is significantly better than standard optical lithography (limited by light diffraction). For comparison, bottom up assembly of nanoparticles (Section 5.3.2) normally provides smaller gaps, but at the price of a lower reproducibility. EBL is not suitable for fabricating large area substrates and is also a very expensive method [403].

*Interference lithography*

Interference lithography [405,406,408] is a relatively simple fabrication process that allows one to fabricate large area periodic patterns with a period of approximately λ/2. Typically, a coherent laser beam is split in two parts that are recombined into a photoresist. The interference between the two waves produces a pattern of minima and maxima of light intensity and, hence, a periodic modulation of the solubility of the photoresponsive polymer. The sample can then be developed analogously to that described above for EBL.

**Figure 37:**
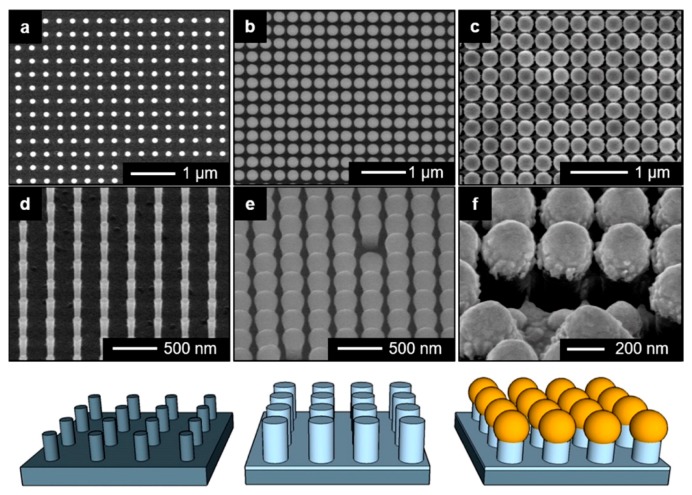
SEM images of the SERS substrates at different fabrication stages. (**a**) Silicon nanopillars fabricated after lithography and etching; (**b**) nanopillars after silica deposition; (**c**) nanopillars after gold deposition. Images in panels (**d**–**f**) are taken at a 45° angle and are enlarged with respect to images in panels (**a**–**c**), respectively. Reproduced with permission from Kanipe et al. [355]. Copyright (2016), the American Chemical Society.

Kanipe et al. [355] fabricated a bi-dimensional square array of silicon nanopillars, with diameter of 120 nm and pitch of 330 nm, by interference lithography with a HeCd laser (325 nm). The nanopillars were covered with a layer of silica (in order to reduce the gap between the nanostructures) and a layer of gold to make them plasmonically active. Figure 37 illustrates the fabrication process. This substrate was optimized by varying the silica and gold thickness and 20 nm turned out to be the most efficient gap size. It is worth mentioning also the approach of Siegfried et al. [356], who fabricated a sub-10 nm gap array by exploiting synchrotron extreme ultraviolet radiation (13.5 nm wavelength) [409]. Both fabrication methods described produced SERS substrates with remarkable enhancing and uniformity properties.

Photolithography, compared to EBL, is a cheaper method, useful for fabricating large samples; on the other hand, only periodic patterns can be written and the gap between the structures is limited to approximately half of the wavelength of the radiation used. Specific strategies, as mentioned above, can be adopted in order to generate gap sizes small enough for SERS applications.

*Soft lithography*

**Figure 38:**
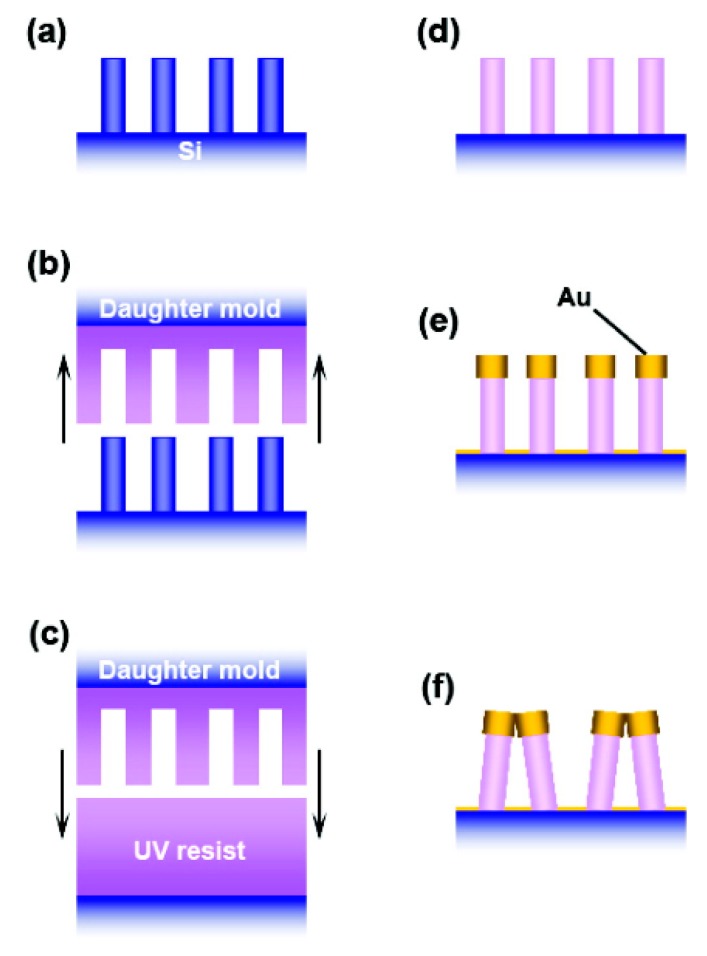
Fabrication of the nanopillar array with tips covered by a gold layer. (**a**) A silicon nanopillar array (master) is fabricated by EBL; (**b**) the master pattern is transferred to the polymer by ultraviolet (UV) curable nanoimprint lithography (NIL); (**c**,**d**) the final polymer nanopillar array is fabricated with a second round of NIL; (**e**) deposition of a gold layer; (**f**) exposition to the solvent and drying makes the nanopillars collapse on each other forming hot spots. Reproduced with permission form Ou et al. [358]. Copyright (2011), the American Chemical Society.

**Figure 39:**
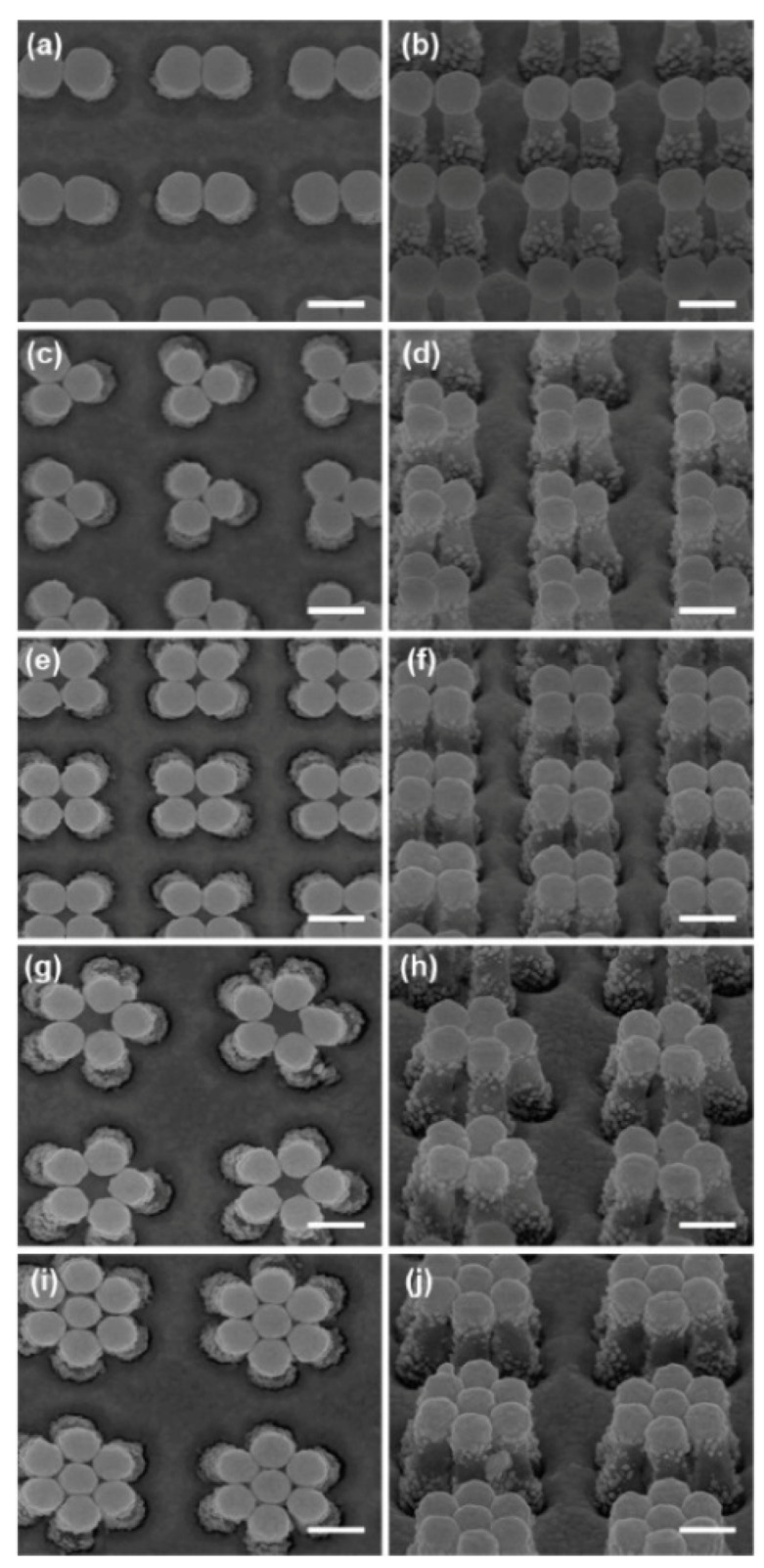
SEM image showing the top view (**a**) and the side view with a 45° angle from the normal (**b**) for digons. Analogous images are reported for trigon (**c**,**d**), tetragon (**e**,**f**), pentagon (**g**,**h**), and hexagon (**i**,**j**) structures. Scale bars in the SEM images are 200 nm. Reproduced with permission from Ou et al. [358]. Copyright (2011), the American Chemical Society.

Soft lithography comprises a number of techniques for micro and nano fabrication through the use of a patterned elastomer as a mask, mold, or stamp [403,405,406,410,411,412,413]. Compared to EBL or photolithography, this method is much cheaper and accessible also to chemists, material scientists, etc. Ou et al. [357,358] used nanoimprint lithography (NIL) to fabricate arrays of polymer nanopillars, whose tips were coated with a gold layer. The polymer nanopillars were about 520 nm tall and the gold layer was 80 nm thick with a diameter of about 140 nm. When these arrays were exposed to a solvent and dried, the capillary forces made the nanopillars collapse on each other, generating hot spots. Figure 38 illustrates the fabrication process. This strategy is analogous to the one used by Lee et al. [350] and described before. Depending on the type of array used, the authors were able to fabricate digon, trigon, tetragon, pentagon, and hexagon structures, as illustrated in Figure 39; the pentagon structure provided the highest SERS signal.

Zhang et al. [359] fabricated a gold coated array of nanocones. The authors used an array of cone-shaped nanoholes (prepared by aluminum anodic oxidation) as a master to pattern a polymer by UV-NIL; subsequently, gold was deposited over the whole surface. The resulting substrate exhibited a good flexibility; moreover, the fact that the whole surface is covered with gold can be an advantage since no Raman signal from the polymer should be generated. Other papers on this subject deal, for example, with the fabrication of nanocylinder arrays [46] and nanowell arrays [360].

Soft lithographic methods allow the fabrication of large area substrates (up to ~1 cm^2^) [410], with a good SERS performance, in a cheaper and more easily accessible way with respect to traditional lithographic methods, like photolithography and EBL.

### 5.4. SERS Labels for Indirect Detection

**Figure 40:**
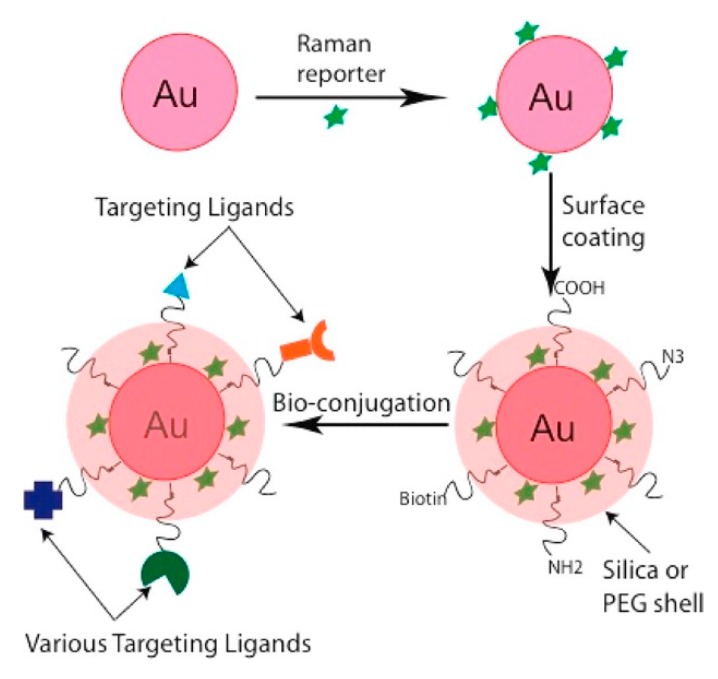
General design of a SERS tag formed by a plasmonic core, a Raman reporter molecule, and a biocompatible layer bearing targeting ligands. Reproduced with permission from Lane et al. [414]. Copyright (2015), the American Chemical Society.

SERS labels essentially comprise a plasmonic core, a Raman reporter, and a protective shell functionalized with targeting moieties [38,94,306,414,415]. Figure 40 illustrates the general design of a SERS tag.

The plasmonic core is preferentially made of gold, owing to its better biocompatibility [167,168] compared to silver [169,170]. Silver is considered more efficient than gold for SERS enhancement; however, this holds for the visible region. Instead, in the first transparency window (see Section 4), where normally SERS spectra are excited in biomedical applications, the plasmonic performance of the two metals is very similar (see Section 3.1.1). The core should be engineered in order to optimize the enhancement in the spectral region of interest; this aim can be pursued, for example, by using nanoparticles with different shapes (i.e., nanospheres [416], nanostars [317], etc.), by aggregating nanoparticles to form dimers or trimers [417,418], or by fabricating more complex structures that possess a high density of hot spots (i.e., core-satellite systems [419]). The introduction in the core of a magnetic functionality allows one to separate the analyte from the (possibly) complex matrix in which it is immersed and to concentrate it [419,420].

The Raman reporter should be photochemically stable and should possess a large cross-section; typically rhodamines, the crystal violet cation, and malachite green are used [414]. With these dyes it is also possible to exploit the resonance Raman effect, if the excitation wavelength falls within or near their optical absorption band; this improves the signal but, on the other hand, increases the chance to overheat and burn the reporter molecules. Other options are small organic molecules, like benzenethiol, that still possess good cross-sections (although no resonant Raman effect can be exploited) and in addition strongly bind to the surface of the nanoparticles [306]. The Raman reporter should also have bands in a clean spectral region, not superimposed with the matrix or other analyte spectra.

A protective shell serves several purposes, namely preventing the nanoparticles from aggregating in the environment in which they are used, avoiding the detachment of the reporter from the surface, and hindering non-specific adsorption of cellular components. Typically, polyethylene glycole (PEG) [421] or silica [422] are used as protecting layers; both can be functionalized with antibodies, aptamers, or peptides for specific analyte targeting [306].

### 5.5. Commercial Substrates

Several companies commercialize nanoparticles of different shapes and solid SERS substrates. Some of them are listed in the following.

- Sigma-Aldrich-Merck KGaA (Darmstadt, Germany) [361] sells silver and gold nanoparticles in solution with different shapes (spherical, rods, plates) and sizes, for example, gold spherical nanoparticles are available from 5 to 400 nm and nanorods are available with an absorption peak ranging from 550 to 1064 nm.
- Nanopartz (Loveland, Colorado, USA) [362] offers a wide selection of nanoparticles in solution (like spherical gold nanoparticles, nanorods, nanowires, and nanocubes), with different sizes, aspect ratios, and different types of capping.
- Nanocs (New York, USA) [363] commercializes mainly gold nanoparticles in solution with different sizes and different types of coating (i.e., bare, biotine, streptavine, dextrane, etc.); some types of silver nanoparticles are available as well.
- Silmeco (Copenhagen, Denmark) [364] offers solid SERS substrates formed by an array of silicon nanopillars, on top of which a silver or gold layer is deposited. The evaporation of the solvent causes the pillars to collapse on each other, forming hot spots (analogously to the substrates described by Ou et al. [357,358] and by Lee et al. [350]). Typically, but substrates can be customized, the SERS active area is 16 mm^2^ (4 × 4 mm). Several publications describe in detail these types of substrates [423,424].
- Horiba Scientific (Minami-ku Kyoto, Japan) [365] provides gold SERS substrates, formed by nanorods processed by dynamic oblique vacuum evaporation. The size of the active area is 4 × 3 mm or 5 × 7 mm.
- Ocean optics (Largo, Florida, USA) [366] offers gold and silver substrates, whose active area is a circle with a 5.5 mm diameter.
- AtoID (Vilnius, Lithuania) [367] commercializes gold and silver substrates fabricated by modifying the surface of soda lime glasses with ultra-short laser pulses, followed by deposition of a metal layer. The active area is 3 × 5 mm.

### 5.6. Some Analytical Aspects of SERS Substrates: Separation and Capturing Techniques

When SERS measurements involve real samples and not simply tests with a single analyte, several aspects make the analysis more complicated. The analyte has to be collected from the sample and it can be present in low concentration; moreover, once extracted, it is in general dissolved in a complex matrix, containing other compounds that can competitively bind to the surface of the SERS substrate or possess a Raman signal interfering with the bands of the analyte of interest. Several techniques have been coupled to SERS in order to overcome the previously mentioned issues and, in general, they can be divided in separation and capturing methods. Interesting reviews on this subject are, for instance, the papers by Zhang et al. [47], Mosier-Boss [35], Porter et al. [425], and Szlag et al. [426].

#### 5.6.1. Separation Techniques

SERS detection has been combined with gas chromatography (GC), high pressure liquid chromatography (HPLC), and thin layer chromatography (TLC).

Heaps et al. [427] eluted, in two different experiments, caffeine and p-nitrothiophenol through a gas chromatographic column, at the end of which a SERS substrate, formed by a thin layer of silver on ZnSe, was placed; it was kept at low temperature in order to condensate the analytes from the gas phase. SERS measurements were carried out after transferring the substrate from the chromatograph to the Raman instrument.

Trachta et al. [428,429] analyzed the presence of drugs in biological fluids, namely urine and blood. The first step was a solvent extraction followed by phase separation via centrifugation. This solution was eluted through an HPLC column and the fractions emerging at different times were collected and deposited on SERS substrates. The eluent was chosen so that it did not interfere with the SERS substrates (acetonitrile was excluded due to its interaction with silver). SERS substrates were formed by a gelatin-based silver halide, that was photoreduced by laser exposure to fabricate the SERS active structures. Several drugs have been identified in this way, like codeine, doxepine, and methadone. Wang et al. [430] coupled the output of an HPLC instrument to a capillary tube, on the internal surface of which metal nanoparticles had been adsorbed. A different capillary tube was used for the analysis of each fraction eluted. This system was used for detecting 4,4-bipyridine, 1,4-benzenedithiol, and the thiram pesticide.

Farquharson et al. [431] synthesized a silver-doped sol-gel that, in a liquid chromatography experiment, was used to separate the analytes and simultaneously played the role of the SERS substrate.

**Figure 41:**
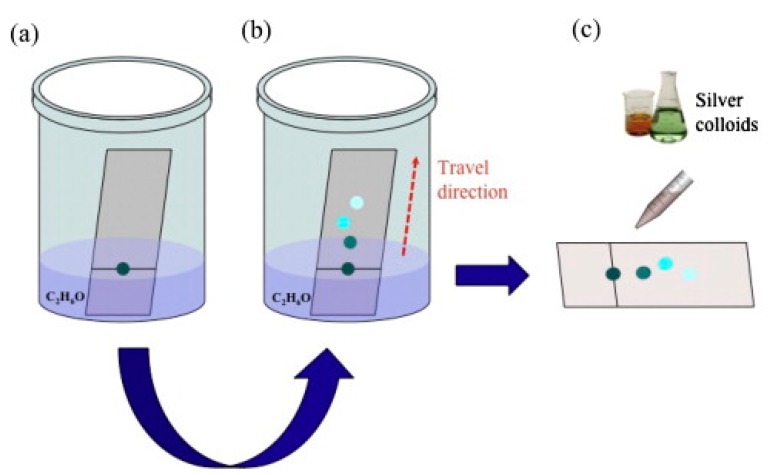
(**a**) A drop of plasma blood containing apomorphine is put on a thin layer chromatography (TLC) slide; (**b**) elution with ethanol; (**c**) a silver colloid solution is dropped on the spots after separation has occurred. Reproduced with permission from Lucotti et al. [432]. Crown copyright (2012), published by Elsevier B.V.

Lucotti et al. [432] analyzed the presence of apomorphine (a drug used in the treatment of the Parkinson’s disease) in human blood plasma. As illustrated in Figure 41, a small quantity of plasma was dropped on the TLC substrate and eluted with ethanol. A drop of colloidal silver nanoparticles was put on the spots formed after separation and the SERS spectra collected. A similar strategy was used by Vicario et al. [433] for the detection of an anticancer drug and by Lv et al. [434] for the detection of ephedrine in slimming food supplements.

#### 5.6.2. Capturing Techniques

Increasing the affinity between the analyte and substrate may serve, in general, two purposes: concentrating the analyte on the SERS active region and separating the analyte from the matrix. In the following we shall report a few examples of the methods that can be used.

**Figure 42:**
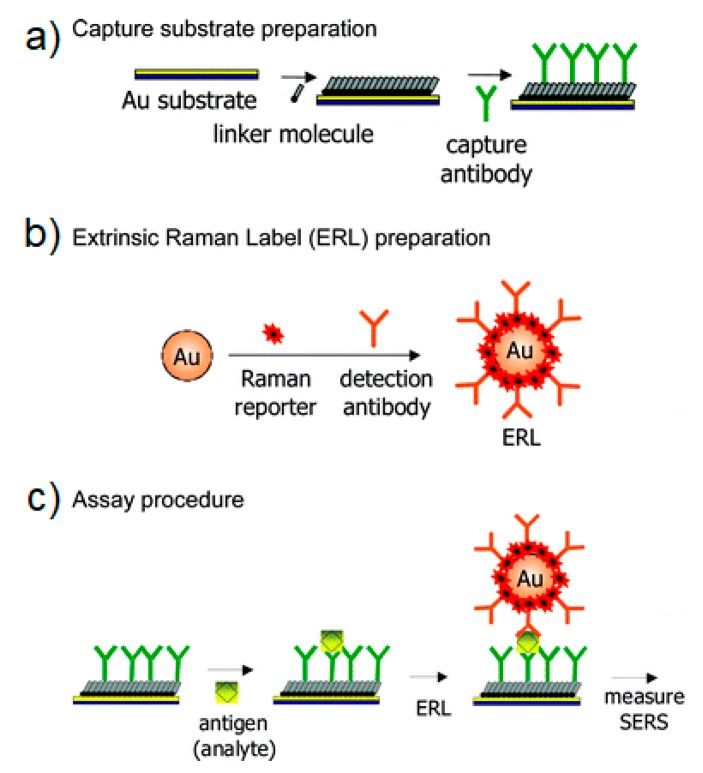
Steps involved in a SERS experiments based on an immunoassay. Reproduced with permission from Porter et al. [425]. (**a**) The substrate is functionalized with a capture antibody; (**b**) The SERS tag is synthesized by assembling a plasmonic core, a Raman reporter and a detection antibody; (**c**) In the assay procedure, the antigen (analyte) is sandwiched in between the SERS tag and the substrate. Copyright (2008), the Royal Society of Chemistry.

Immunoassays are biochemical tests that rely on the ability of antibodies to selectively bind specific analytes (antigens). The most relevant steps involved in a SERS experiment, based on an immunoassay, are illustrated in Figure 42 [425]: (a) A SERS substrate is functionalized with the specific antibody able to bind the analyte (antigen) of interest; (b) in an indirect detection scheme, a SERS tag (extrinsic Raman label in the figure) containing a plasmonic core, a Raman reported molecule, and functionalized with the antibodies is prepared. In the direct detection scheme (label free) the tag does not contain the Raman reporter and the analyte is identified through its own Raman spectrum; (c) in the assay procedure, the analyte is sandwiched in between the substrate and the tag and SERS analysis can be carried out. This method has been used for example for the detection of a cancer biomarker (prostate specific antigen) [435], feline calicivirus [436], Hepatitis B virus [437], cytokine [438], and allergen proteins [439].

**Figure 43:**
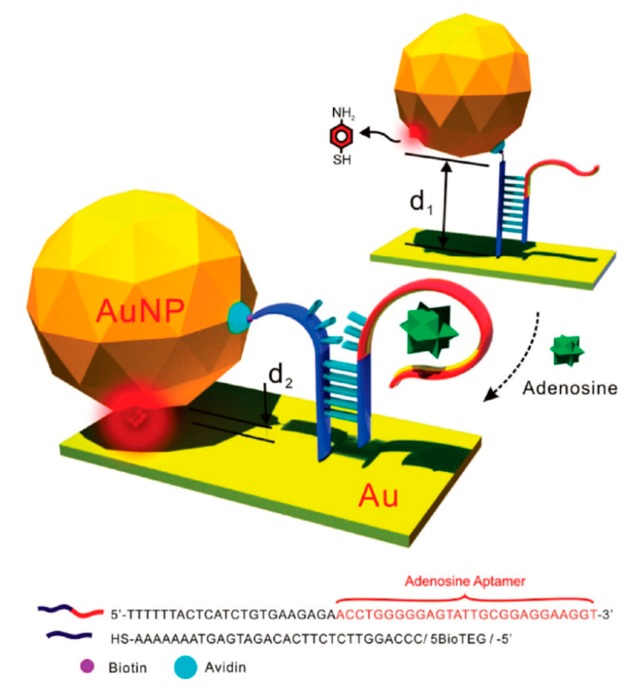
Example of aptamer-based SERS detection of adenosine. Reproduced with permission from Kim et al. [441]. Copyright (2010), American Chemical Society.

Aptamers are single stranded pieces of DNA (ssDNA) or RNA, normally 10 times smaller than antibodies, that bind to a specific target molecule. SERS aptasensors have been proposed, for example, by Li et. [440]: The authors functionalized a gold film with complementary DNA (cDNA), that was hybridized with an aptamer-labelled SERS tag. In presence of adenosine triphosphate (ATP), the duplex DNA structure was dissociated, due to the interaction between ATP and the aptamer, releasing the tag and causing a reduction of its SERS signal. In another interesting example, Kim et al. [441] engineered a system in which a partially-hybridized double-stranded DNA (ds-DNA) aptamer is linked to a gold surface, standing approximately erect (Figure 43). One strand (the shortest) was attached to a gold nanoparticle functionalized with a SERS active molecule (4-aminobenzenethiol, 4-ABT) and the other (longer) strand, contained a non-hybridized adenosine aptamer. The presence in solution of adenosine, induced a dehybridridization of the double stranded structure, causing the gold nanoparticle to become closer to the surface and; therefore, amplifying the SERS signal from 4-ABT.

Self-assembled monolayers (SAM) of aliphatic thiols or amine are often used to cover metallic substrates; they provide a protection for the substrate and also improve its affinity towards some types of analytes. Shafer-Peltier et al. [442] studied the SERS detection of glucose, a compound very difficult to measure in SERS, owing to its small cross-section and its very low affinity to metals. The authors used 1-decanethiol to form a SAM on a silver substrate: the SAM was approximately 1.9 nm thick and exhibited good affinity to glucose; therefore, concentrating it close to the surface (several other molecules were tested but straight alkane thiols turned out to be the most efficient ones in capturing glucose). Olson et al. [443] used 1-octadecanethiol on gold to promote the adsorption of aromatic hydrocarbons like naphthalene and phenanthrene with a quick kinetics (less than 5 min). Huang et al. [444] exploited the ability of β-cyclodextrin to encapsulate apolar molecules of the right size, owing to their hydrophobic cavity, to improve the sensitivity of a SERS substrate towards polychlorinated biphenyls (PCBs) in water.

**Figure 44:**
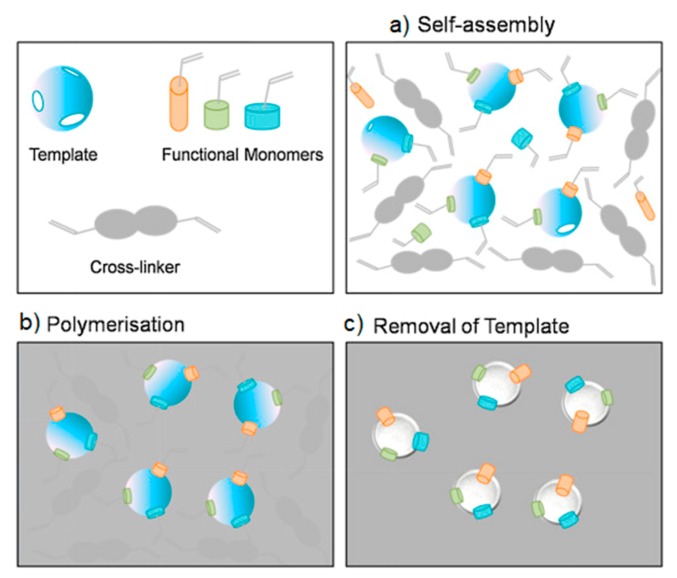
Illustration of the synthesis of molecularly imprinted polymers. Reproduced with permission from Wackerlig et al. [445]. Copyright (2014), 2014 Elsevier B.V.

Molecular imprinted polymers (MIPs) are artificial materials that can be engineered to make them affine to selected chemical or biological species. The main steps involved in the preparation of MIPs is illustrated in Figure 44 [445]: (a) A template molecule, a cross-linker, and functional monomers are mixed together in solution; (b) polymerization occurs; (c) after removal of the template, a cavity is formed in which recognition sites are exposed. Typically, these sites form non-covalent bonds with the guest analyte, based on ionic, hydrogen bond, or hydrophobic interactions. MIPs are sometimes referred to as artificial antibodies [47]. BelBruno et al. [446] recently reviewed the field of MIPs and their application in sensing biomarkers, food contaminants, drugs of abuse, pathogens, etc. Concerning the specific case of the SERS detection, Holthoff et al. [447] synthesized a MIP doped with 2,4,6-trinitrotoluene (TNT) and spun cast it on a commercial SERS substrate. TNT was then removed with a suitable mixture of solvents, leaving cavities free for analyte capture. Incubation of the substrate in solutions of TNT at different concentrations were used to test the properties of the SERS–MIP combined method. Xue et al. [448] covered gold nanoparticles with a layer of MIP using bisphenol A (BPA) as a template and extracting it by heating the solution. Instead, Hu et al. [449] adopted a different strategy, by adding AgNO_3_ to the monomer–cross linker–template (melamine) mixture. After polymerization was accomplished, the reduction of silver nitrate to silver nanoparticles was carried out with sodium borohydride; the template was finally removed by Soxhlet extraction leaving an integrated silver nanoparticle–MIP system. This latter was used to detect melamine from water solutions and milk.

Antibodies are very specific capture agents but, on the other hand, they are also very costly. Instead, aptamers are a cheaper alternative and also can be applied to many target categories. SAM and MIPs are the most economical alternative. The former are suitable for detecting small molecules, while the latter can be fabricated to detect both small and large molecules. Both methods can be tailored for different analytes, in particular MIPs [426]. When using capturing agents, it is important to evaluate their (eventual) contribution to the Raman spectrum that should be as small as possible; moreover, they should keep the analyte sufficiently close to the surface of the substrate in order to efficiently exploit the enhancement mechanisms.

## 6. Applications in the Biomedical Field

In the following, a selection of SERS applications in the biomedical field is presented. They are summarized in Table 8 and Table 9 for direct and indirect protocols, respectively.

### 6.1. Direct Protocol

Label-free SERS experiments have been performed on many different biological species, ranging from amino acids, peptides, purine, and pyrimidine bases and proteins, to DNA, RNA, chlorophylls and other pigments, molecules containing chromophores (like heme-containing proteins), stimulating drugs, and antitumor drug interaction with DNA and bacteria [59,96,305,492,493,494,495,496,497,498,499,500,501,502,503,504], reaching a high sensitivity, even at single molecule level [243,505,506,507]. Colloidal gold and silver nanoparticles have been used as SERS sensors also in living cells, to enhance the Raman signal of intracellular components and to gain information on both the composition and the dynamics of the cells [508]. An overview, albeit not exhaustive, of many different applications of label-free SERS will be reported herein. Silver electrodes and colloidal silver nanoparticles are the most common SERS-active substrates used in solution. They have been used to investigate the dynamics of the structural DNA fluctuations [509], to distinguish neurotransmitters like dopamine and norepinephrine, with similar structures, in the near-IR range at nanomolar concentrations (using short accumulation times, as short as 25 ms) [510], to reach single molecule detection, as observed with adenine and adenosine monophosphate, and to detect single mismatch in a double stranded DNA (ds-DNA) fragment [511].

#### 6.1.1. DNA Detection

**Figure 45:**
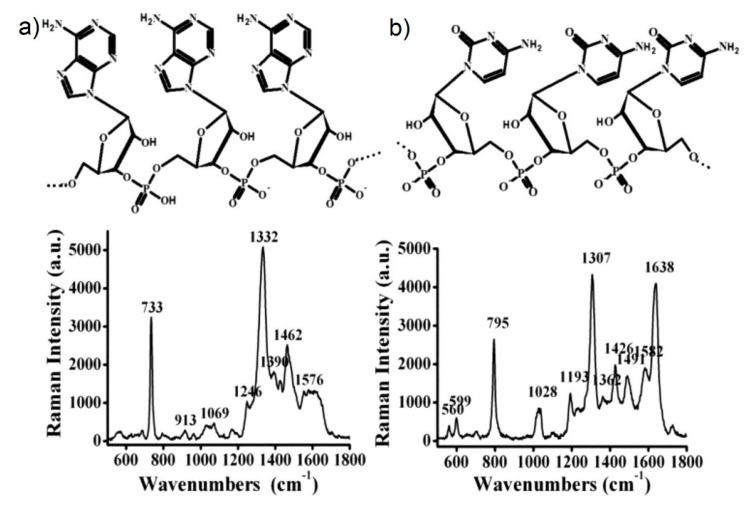
Structures and SERS spectra of single-strand polyadenosine (pA, 10 mers, at 1 nmol) (**a**) and single strand polycytidine (pC, 10 mers, at 2 nmol) (**b**) (conditions of acquisition: λex = 514.5 nm, time 60 s, and laser power = 10 mW at the sample). The purine base, pA, exhibits two major peaks at 733 cm^−1^ (ring breathing) and 1332 cm^−1^, assigned to the ring stretching mode that can be used as marker bands. The pyrimidin base exhibits the ring breathing mode and the ring stretching mode, at 795 and 1307 cm^−1^, respectively, and 1636 cm^−1^ band assigned to the C=O vibration. Reproduced with permission from Prado et al. [451]. Copyright (2014), the American Chemical Society.

The detection of potential biomarkers of disease pathogenesis, including many cancers, can be performed also by identifying the sequence of micro RNA (miRNA), since it functions as a regulator of gene expression. Silver nanorod arrays have been successfully used as SERS plasmonic substrates for sensitive and rapid detection of miRNA members and family members and their pattern classification [450]. Alternatively, silver nanocolloid aggregates have been used in microfluidic devices to enhance the Raman signal of label-free single-strained RNA bases; the signal of adenine and cytosine are the markers of purine and pyrimidine bases in single strands. The SERS spectra show that the nucleobases can be selectively detected even in few nanoliters droplets (Figure 45) [451].

**Figure 46:**
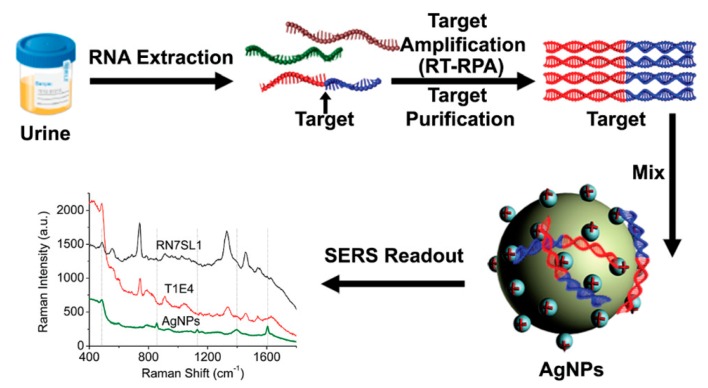
RNA biomarkers detection through label-free SERS. The detection can be represented considering four different steps. Step 1: Extraction of RNA from urinary samples. Step 2: Amplification of target RNA biomarkers into dsDNA sequences, by isothermal transcription-recombinase polymerase amplification (RT-RPA) and purification of samples. Step 3: Incubation of amplicons with positively-charged Ag nanoparticles. Step 4: SERS measurements of colloidal suspensions. Reproduced with permission from Wang et al. [458]. Copyright (2017), the Royal Society of Chemistry.

The SERS analysis of RNAs at the ultrasensitive level has been performed also using positively charged spermine-coated silver nanoparticles. Using these substrates, the SERS signal allows one to identify and classify similar RNA structures; by detecting their conformation and composition, it has been possible to recognize fully complimentary duplexes, short hairpin and small RNAs, and to diversify miRNA sequences, by individuating chemical differences, like single-base variances, nucleobase modifications, and backbone composition [452]. A different approach is based on the amplification of multiple RNA biomarkers, based on multiplex reverse transcription-recombinase polymerase process, and their detection through SERS on silver nanoparticles; by this way it is possible to distinguish long amplicons (∼200 bp). The RNA extracted from urine samples has been analyzed, to detect prostate cancer with a non-invasive strategy, obtaining good results in term of specificity (93.0%), sensitivity (95.3%), and accuracy (94.2%) (Figure 46) [458].

#### 6.1.2. Analysis of Cellular Functions and Components in the Cell Microenvironment

**Figure 47:**
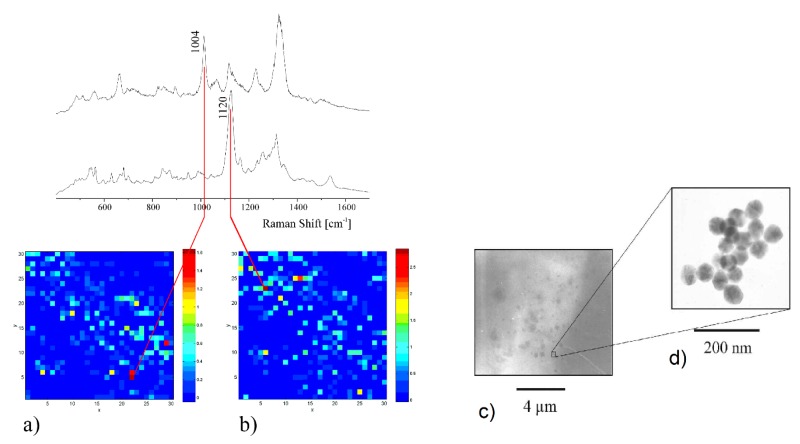
Example of SERS imaging. Distribution of the maximum Raman signal of phenylalanine, Phe– at 1004 cm^−1^ (**a**) and DNA, O–P–O DNA backbone at 1120 cm^−1^ (**b**) over a 30 × 30 mm^2^ cell. The maximum of the two Raman signal occurs at different places considering that DNA is mainly located in the cell nucleus, while phenylalanine should be mainly present in the cytoplasm. Electron micrograph of Au nanoparticles inside a cell (**c**). Magnification showing 60-nm gold colloidal sphere aggregates (**d**). Reproduced with permission from Kneipp et al. [453]. Copyright (2002), Society for Applied Spectroscopy.

To investigate the chemical species inside cells, it is necessary to incorporate the plasmonic nanoparticles into the biological samples. To this end, gold colloidal nanoparticles have been demonstrated to be the most suitable, thanks to their chemical inactivity and biocompatibility, and to their high Raman enhancement factor in the biological window excitation range, favored by the formation of colloidal aggregates [506,508,512,513]. Gold colloidal nanoparticles allow a sensitive and selective tool for detection of chemical species inside cells and for monitoring their distributions. Moreover, by properly designing the size, the shape, and the surface functionalization of the gold nanoparticles, it is possible to define which cellular barrier will be crossed and where the nanoparticles will be localized within cells of living systems [454]. Gold nanoparticles with 60 nm diameter were incorporated into cells by fluid phase up-taking, during the growing process, or by ultrasound sonication, and afterwards the presence of salts induced their aggregation, thus increasing the Raman signal [453]. The SERS signal provided information on the cell structure, allowed the observation of structural modification, and enabled even the Raman mapping of a cell. The distribution of phenylalanine and DNA over a 30 × 30 mm^2^ cell monolayer, intestinal epithelial cells HT29 has been individuated (Figure 47a,b) using incubated 60 nm gold nanoparticles (Figure 47c,d) [453].

**Figure 48:**
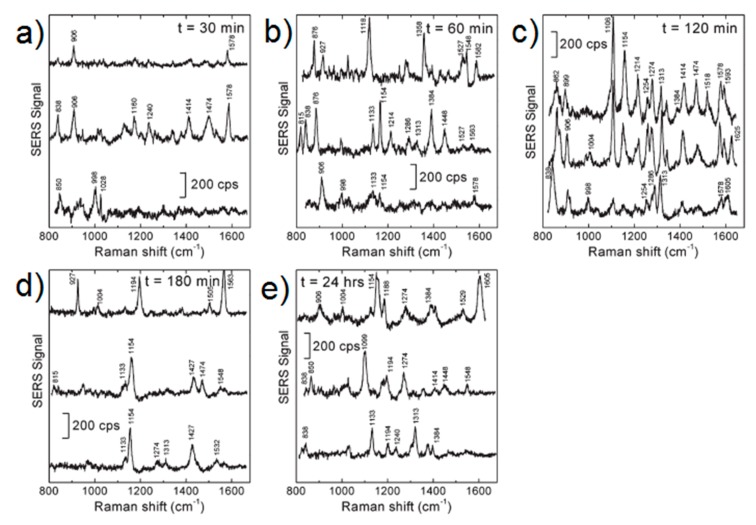
SERS spectra acquired from living soft epithelial cell line IRPT (immortalized rat renal proximal tubule) in phosphate buffered-saline, by raster scanning over individual cells, after different times of incubation (30 min (**a**), 60 min (**b**), 120 min (**c**), 180 min (**d**), 24 h (**e**)) with gold nanoparticles. Reproduced with permission from Kneipp et al. [454]. Copyright (2006), the American Chemical Society.

**Figure 49:**
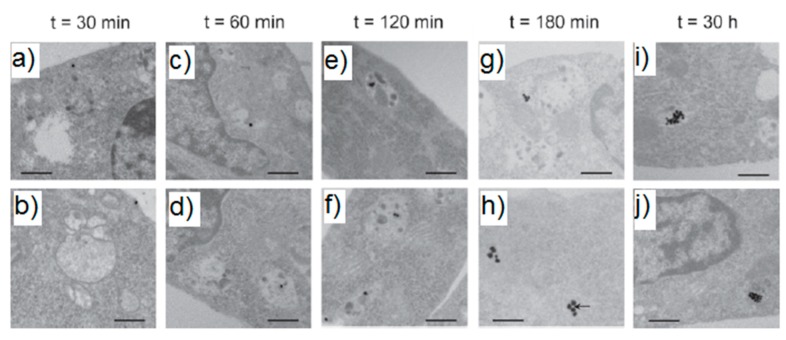
Transmission electron micrographs of immortalized rat renal proximal tubule (IRTP) cells at different incubation times (the same time points of the SERS micro-spectroscopic data, reported in Figure 48). The black, electron-dense spots, visible in the cells, are the gold nanoparticles. The nanoaggregates size varies with incubation time. After 30 min (**a**,**b**) and 60 min (**c**,**d**) aggregates are not evident. After 120 min (**e**,**f**), nanoclusters of 2–3 particles are visible; after 180 min (**g**,**h**), 4–6 particles and larger lysosomal nanoaggregates during overnight incubation (**i**,**j**) of the cells are formed. After 180 min the interparticle distance (see black arrow in panel (**h**)) is greater, likely because of the enclosure of the particles in multivesicular structures. Scale bars (**a**–**g**,**i**,**j**): 500 nm; (**h**): 250 nm. Reproduced with permission from Kneipp et al. [454]. Copyright (2006), the American Chemical Society.

The endosomal system of cultured eukaryotic cells (living epithelial and macrophage cells) has been investigated using gold nanoparticles with 30–50 nm diameter, a dimension which allowed one to reach the most efficient endocytosis. Using a low excitation intensity and fast collection times it is possible to obtain Raman signal amplified from the position where nanoparticles are temporarily localized (Figure 48 and Figure 49 show the Raman spectra and the corresponding TEM images of the cells, obtained at different incubation times, respectively), giving information on the molecular composition in the “nanometer-vicinity” of the flowing plasmonic nanoparticles. By observing the differences in the SERS spectra obtained over time, it is possible to characterize the changing cellular environments and to probe the cellular compartments. Furthermore, information concerning chemical properties, such as the local pH, could also be obtained, with higher lateral resolution with respect to other techniques [454].

**Figure 50:**
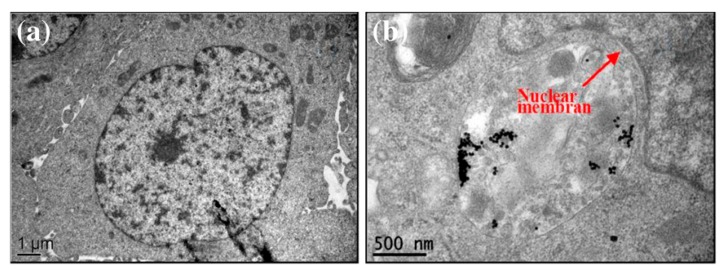
(**a**) TEM image of human breast cancer cell, of approximately 10 µm diameter, showing cell structures, like nucleus and nuclear membrane; (**b**) TEM image of cell incubated with gold nanoparticles, which reside in cytoplasm and are enveloped into some vesicles (“lick up vesicles”); gold nanoparticles are clearly aggregated. Reproduced with permission from Zhu et al. [456] under Creative Commons 2.0 license (https://creativecommons.org/licenses/by/2.0/).

**Figure 51:**
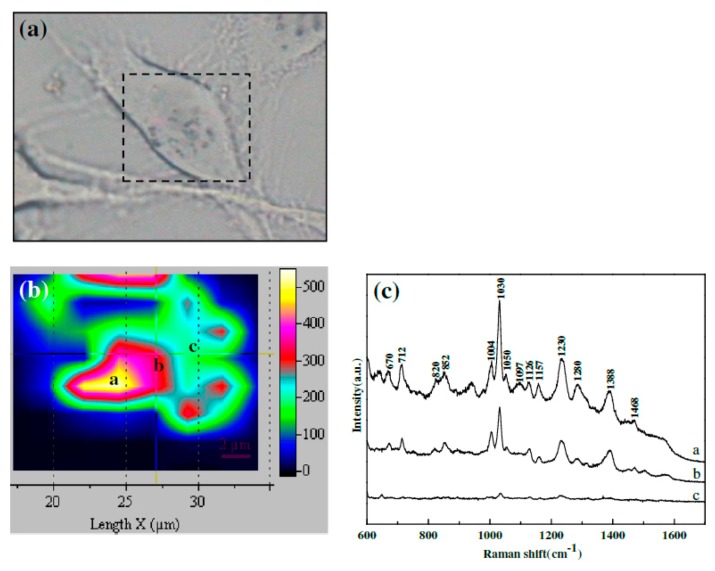
Photomicrograph of a fixed breast cancer cell (**a**) and SERS mapping image (**b**), obtained by recording the Raman signal at 1030 cm^−1^ in the rectangle region, with 10 × 10 μm^2^ dimension (outlined in micrograph (**a**)). The Raman signal at 1030 cm^−1^ corresponds to the C–H in-plane bending mode of phenylalanine. SERS spectra recorded in position (**a**–**c**) of the labelled area (**c**). Reproduced with permission from Zhu et al. [456] under Creative Commons 2.0 license (https://creativecommons.org/licenses/by/2.0/).

Raman has been used also for the detection of different types of cancer. Silver nanoparticles, with a diameter of 34 nm, have been employed as SERS-substrates, directly mixed with blood plasma, for non-invasive gastric cancer detection. Amplified Raman spectra recorded using left-hand circularly polarized laser light excitation, have clearly differentiated the plasma of gastric cancer patients from the healthy one, with a sensitivity of 100% and specificity of 97% [455]. The analysis of cancer cells in live cells, obtained from biopsies, has been performed through SERS using both silver [457] and gold colloidal nanoparticles aggregates [456]. Using silver nanoparticles, several Raman peaks have been strongly enhanced, allowing, thus, the identification of chemical constituents, like phenylalanine, tyrosine, and tryptophan, and adenine and guanine (in DNA) [457]. The TEM images of the human breast cancer cells, of approximately 10 μm diameter, incubated with gold nanoparticles, with 35 nm diameter (corresponding to an absorption centered at 520 nm), clearly show the presence of gold aggregates, localized in cytoplasm and enveloped into vesicles (Figure 50). By recording the Raman signal at 1030 cm^−1^ (corresponding to the C–H in-plane bending mode of the substituted benzene in phenylalanine interacting with Au nanoparticles) over the 10 × 10 μm^2^ area of the cell, a SERS mapping of the cells has been obtained (Figure 51) [456].

#### 6.1.3. Protein Detection

**Figure 52:**
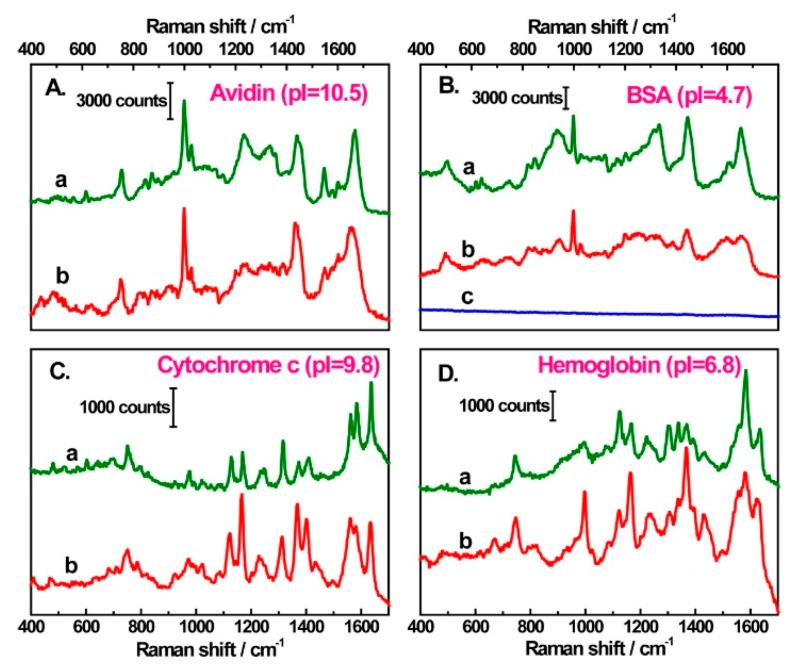
Comparison of the normal Raman (green line, a) and amplified SERS spectra (red line, b) of Avidin (**A**), BSA (**B**), Cytochromo c (**C**), and Hemoglobin (**D**). SERS spectra are obtained with the iodide-modified Ag nanoparticles method, using sample concentrations of 300, 300, 3, 30 μg/mL, respectively, aggregated by MgSO4. The blue line c, in BSA spectra (**B**), evidence the aggregation effect: No Raman signal is detected before aggregation. Raman spectra of avidin, BSA, and Hemoglobin solid are obtained with 20 mW laser power and 30 s acquisition time. Reproduced with permission from Xu et al. [465]. Copyright (2014), the American Chemical Society.

Different methods have been employed to characterize proteins through SERS [514]. Time-resolved SERS has been used to investigate the dynamics of the redox processes of cytochromes, adsorbed on Ag electrodes, thus providing kinetic and structural information about electron transfer reactions of adsorbed monolayers [460]. Raman spectra have been recorded from myoglobin attached to 100-nm-sized immobilized Ag particles [461] at the single molecule level. Alternatively, SERS has been combined with Western blot, by using colloidal silver nanoparticles, to identify label-free multi-proteins. Colloidal silver nanoparticles, adsorbed on nitrocellulose membrane, where proteins had been previously adsorbed, allowed the easy registration of SERS spectra of myoglobin and BSA, reaching a detection limit as low as 4 ng [462]. The protein-nanoparticles interaction and the native structure of proteins have been also characterized using resonant SERS with silver nanoparticles, in a flowing system, where it was possible to preserve the native structure of the heme-proteins and to gain in-depth experimental information into the Raman enhancement mechanism [463]. The detection of label-free oligonucleotides, of 12–14 base pairs at a concentration of 10^−7^ M, has been performed using silver nanoparticles, as SERS plasmonic substrate, in presence of spermine as charge neutralizer and aggregation agent [464]. More recently, a new method has been proposed to enable the investigation of proteins by retaining them in the native state. It consists of using iodine-modified silver colloids, that reduces the denaturation, while maintaining a good reproducibility and sensitivity of the SERS signal. The SERS spectra of hen egg white lysozyme, avidin, cytochrome c, hemoglobin, and BSA, recorded using these nanoparticles, are similar to the Raman ones recorded in solution or in solid samples (Figure 52) [465].

#### 6.1.4. Viruses and Bacteria

**Figure 53:**
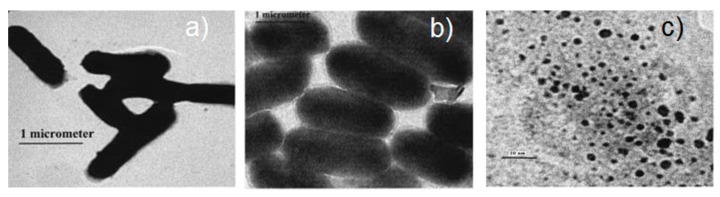
TEM of *Escherichia coli* with Ag colloid deposited on the bacterial wall (**a**), with Ag internal colloids (**b**), and with internal colloids released also into solution from damaged cells (**c**). Reproduced with permission from Efrima et al. [466]. Copyright (1998), the American Chemical Society.

Ag nanocolloids have been used for the first time on bacterial cells by Efrima et al. [466]; they investigated *Escherichia coli* bacteria using silver nanoparticles produced both inside the bacteria and on the outer wall (Figure 53). In particular, very intense signals have been obtained on the outer wall, thanks to the formation of silver aggregates, inducing the amplification of the Raman signal belonging to proteins, peptides, amino acids, molecules, and functional groups present in the immediate proximity of the colloidal nanoparticles.

**Figure 54:**
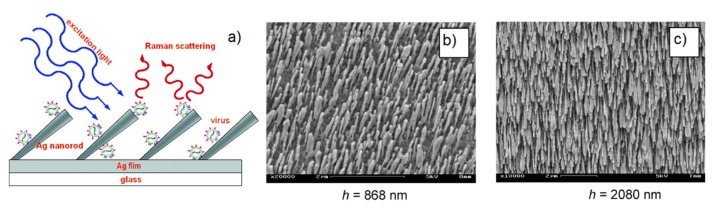
Scheme of silver nanorod array substrates fabricated using electron beam/sputtering evaporation (E-beam) system, in oblique angle deposition (86°), on a 500 nm Ag thin film base layer (**a**), and SEM images of two samples with different nanorod length h = 868 nm (with a diameter of 99 nm) (**b**), and h = 2080 nm (**c**). Reproduced with permission from Shanmukh et al. [467]. Copyright (2006), the American Chemical Society.

Silver nanorod arrays have been used as plasmonic substrates to investigate respiratory human viruses in real-time, at trace levels. SERS substrates have been developed and fabricated using the oblique angle deposition method. SERS spectra were recorded from extremely small volumes of samples and allowed differentiating between respiratory viruses, viruses’ strains, and genetically modified viruses (Figure 54) [467].

A SERS active substrate of 60–80 nm diameter Ag nanocrystals assembled on Ag nanospheres has been tested to investigate pathogenic bacteria; the Raman signal was collected from cells as few as 10 colony forming units/mL (CFU/mL) of three key pathogens (*Escherichia coli* O157, *Salmonella typhimurium*, and *Staphylococcus aureus*). SERS spectra clearly allowed to distinguish them and to understand if bacteria were alive or dead [468]. Colloidal silver nanoparticles have also been used in microfluidic devices to investigate bacteria cells of nine different *E. coli* strains; the different species have been identified obtaining high specificity and reproducibility of spectral information and minimized recording times [63]. Recently, silver-gold bimetallic SERS substrates have been realized and tested to demonstrate the possibility of distinguishing *Listeria monocytogenes* bacteria at strain level, by distinguishing if they belong to different or to a single geno-sero group, to individuate the degree of hazard of the bacterium [469].

### 6.2. Indirect Protocol

In this section, we will discuss biomedical applications employing SERS tags as substrates for the indirect detection of DNA, miRNA, cell biomarkers, proteins, and molecules present in the cell microenvironment.

#### 6.2.1. DNA Detection

Detection of DNA sequences provides important information for a variety of applications, including disease diagnosis, identification of mutations in genes, and detection of pathogens and viral strains. Current DNA detection methods include PCR and fluorescence, which have limited multiplexing capability and are prone to contamination issues. SERS-based DNA biosensors offer high sensitivity with low sample concentrations, thereby eliminating the need for amplification and potential contamination issues [515].

**Figure 55:**
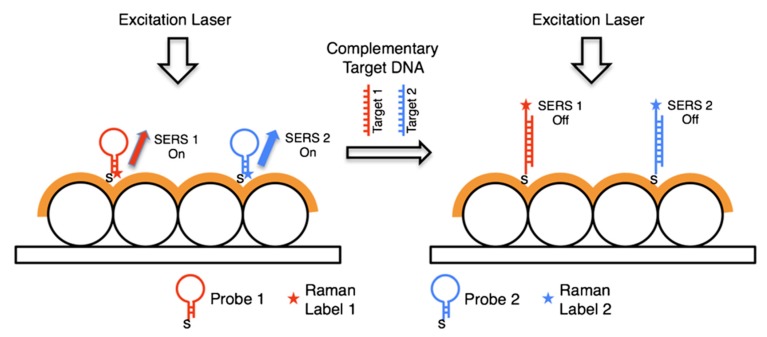
Schematic representation of the assay developed by the Vo-Dinh group employing the molecular sentinel (MS) approach. Reproduced with permission from Ngo et al. [66]. Copyright (2014), Springer-Verlag Berlin Heidelberg.

Vo Dinh’s group developed a “molecular sentinel” (MS) technique to sensitively detect multiple viral DNA sequences [470]. These sentinels consist of hairpin DNA structures with a Raman dye at one end and a thiol moiety at the other *terminus*, which allows binding of the oligonucleotide to the gold nanoparticle. In the absence of the target sequence, the hairpin (or molecular sentinel) forms a stem and loop structure which results in a high SERS signal, due to proximity of the fluorescent dye to the metallic surface. Upon hybridization of the target with the molecular sentinel, and subsequent unfolding of the hairpin, the Raman dye moves away from the metal surface leading to a reduction of the SERS signal. This “on to off” SERS approach was further developed to a molecular sentinel-on-chip (MS-on-Chip) assay, where several MS probes were immobilized on a nanowave chip and used to look at the RSAD2 gene, which is a common inflammation biomarker [471]. The MS-on-Chip technique was also applied for multiplexed detection of two host genetic biomarkers for respiratory viral infection, the interferon alpha-inducible protein 27 (IFI27) gene and the interferon-induced protein 44-like (IFI44L) gene. Here, two MS hairpins, one for each gene, were used with two different Raman labels (Cy5 and ROX). They were checked for target affinity, both individually and in a mixture, to evaluate multiplexing capability of the device. Upon detection of the complementary DNA, a decrease in the SERS signal for both Cy5 and ROX was observed (Figure 55) [66]. Since the change in SERS signal from “on to off” is not intuitive, the authors introduced a parameter called relative diagnostic index (RDI) [471], which could be used to analyze the change in SERS signal upon binding to its target. Wang et al. [472] used a similar method to detect miRNA via the combination of SERS and fluorescence. They developed a microfluidic chip immobilized with silver nanoparticles and attached a molecular beacon targeting miRNA. The beacon consisted of a thiol group, which facilitated attachment to Ag nanoparticles and a fluorophore (6-FAM), which also is a Raman reporter. In the absence of the target miRNA, the molecular beacon formed a hairpin loop, which quenched the fluorescence signal and increased the SERS signal. When the target miRNA was introduced, it hybridized with the beacon, thereby increasing the distance between 6-FAM and Ag nanoparticle surface, causing a decrease in SERS signal and an increase in fluorescence. Using this technique, they presented a method that could be used toward the investigation of miRNA-related diseases.

To avoid the possibility of false positive results with the SERS “on to off” approach, Vo Dinh and coworkers [67] developed an inverse MS-on-Chip technique, with an “off to on” SERS signal change upon target binding. In the absence of target DNA oligonucleotides, the MS probe with the reporter dye forms a partial duplex structure with a placeholder DNA strand that keeps the reporter molecule away from the metal surface. When the target sequences are introduced, the placeholder DNA forms a hybrid with the target, while the MS probe forms a stem and loop structure and brings the reporter close to the surface of the metal, which increases the SERS signal. This “off to on” SERS technique was found to be really beneficial for a single step detection of DNA, without any washing steps and reduced false positive results.

Guven et al. developed a direct and sandwich-based assay to sensitively detect miRNAs, targeting specifically miR-21, a biomarker that is overexpressed in several cancers. miRNAs are small non-coding RNAs containing about 19 to 25 nucleotides that regulate thousands of protein encoding genes [473] (the authors called the two methods “direct” and “sandwich based” but both make use of a Raman reporter; therefore, the “direct” assay described by Guven et al. should not be confused with the direct protocols meant in this review that refer to label-free methods). In the direct detection method, the authors developed a substrate consisting of gold nanorods immobilized on a gold substrate and hybridized with the target miR-21 probes. In the sandwich method, the target miR-21 was captured by a target probe functionalized on a gold slide. It was then hybridized with a second miR-21 probe that was immobilized on gold nanorods containing the reporter 5,5′-dithiobis(2-nitrobenzoic acid) (DTNB). Both assays were found to offer quick and sensitive detection of miR-21 with detection limits for the direct and sandwich assay, at 0.36 and 0.85 nM, respectively. The Halas group recently developed a novel substrate [184] with aluminum nanocrystal aggregates capable of substantial near-infrared SERS enhancements. The surface oxide of Al nanocrystals has preferential affinity for the single-stranded DNA phosphate backbone, leading to an analyte-nanoparticle interaction that preserves the spectral features observed in Raman. They proposed these novel low-cost SERS substrates as the first to quantitatively detect ssDNA, with no modification to either the ssDNA and the substrate surface. These biosensors show promise for as-is detection and quantification for a wide variety of biological molecules. Silicon nanowire arrays decorated with Au nanoparticles (AuNPs@-SiNWAr) have been reported to have high enhancement factors when used as biosensors [516]. Wei et al. [474] recently used DNA strands functionalized on AuNPs@-SiNWArs to detect DNA up to 10 fM concentration. They demonstrated the use of these sensitive biosensors to identify single-base mismatches and multiplexed detection of DNA.

Ilkhani et al. [475] recently developed a sensor to look at the effects of chemotherapeutic drugs on DNA modification/damage in cancer cells. For this purpose, they developed a gold disk electrode that was coated with reduced graphene oxide. They then functionalized it with gold-coated magnetic nanoparticles functionalized with a DNA probe to target the breast cancer gene BRCA1. These nanobiosensors were then subjected to a model chemotherapeutic drug, doxorubicin (DOX). The authors investigated the effects of dosage on DNA modification and were able to understand the mechanism of binding between drug and DNA. These novel biosensors open up opportunities to assess interactions of new drugs with DNA in a cost-effective manner. Li et al. [477] developed a novel substrate for the multiplexed detection of cancer biomarkers, carcinoembryonic antigen (CEA) and α-fetoprotein (AFP). They developed a sandwich assay with a substrate consisting of ordered gold nanohoneycomb arrays that was used for capturing CEA and AFP in solution. Gold nanostars functionalized with Raman reporters and target antibodies were then added to the substrate for simultaneous detection of the two biomarkers. The substrates were able to achieve detection limits of 0.41 and 0.35 ng/mL for CEA and AFP, respectively. These probes were later employed successfully to detect the biomarkers in clinical serum samples with minimal cross-reactivity, thereby establishing them as effective tools for clinical diagnostic applications. In addition to detection of disease markers, DNA biosensors have also been used for pathogen detection. Kang et al. [476] used the Au nanoparticle-on-wire system, employing probe, target, and reporter DNAs to detect four pathogenic bacterial DNA strands from clinical samples in patients. They were able to isolate pathogenic DNA (*Enterococcus faecium, Staphylococcus aureus, Stenotrophomonas maltophilia, Vibrio vulnificus*) from various clinical specimens and achieved a low detection limit of 10 pM. Waluk and co-workers developed a unique SERS substrate for detection of pathogenic bacteria, namely, *Escherichia coli, Salmonella enterica, and Staphylococcus epidermidis* from blood samples [478]. They used an electrochemically-roughened nanoscopic silver substrate, upon which a silver-gold layer was deposited via potentiostatic electrodeposition. They coated the surface of substrates with antibiotics that selectively captured bacteria from clinical blood samples. These examples demonstrate that SERS biosensors can be used for the diagnosis of clinical pathogens.

#### 6.2.2. Analysis of Cellular Functions and Components in the Cell Microenvironment

Changes in the cell microenvironment, such as intracellular pH, production of reactive oxygen species (ROS), and redox potential, as well as formation of cellular gases, such as carbon monoxide (CO) and nitric oxide (NO), play a crucial role on cellular functions. Monitoring these physiological characteristics as well as cell signaling pathways could facilitate a greater understanding of changes occurring during diseases. Of late, several SERS probes are being developed to detect these changes sensitively.

Intracellular pH of cellular components is one of the most important factors necessary in understanding cell physiological functions. SERS-based pH sensors typically consist of Raman active molecules that are covalently attached to either gold or silver nanoparticles, which then cause changes in the SERS signal as a function of the pH of the surrounding media [480]. However, most nanoparticle-based pH biosensors fail either due to aggregation of particles when detecting samples with high ionic strength or in acidic medium, or because of adsorption of proteins, such as bovine serum albumin (BSA) and macromolecules, on the probe at physiological conditions, which affect measurements and sensitivity. To overcome this challenge, Wang et al. [479] recently developed a pH nanosensor, where they encapsulated silver nanoparticles functionalized with the pH sensitive 4-mercaptobenzoic acid (4-MBA) (Ag-MBA@SiO_2_) with a 30 nm thick layer of silica that prevented BSA molecules from interacting with the nanoparticles. Encapsulation also improved the colloidal stability and improved the reliability of the sensor. They demonstrated that these Ag-MBA@SiO_2_ probes could successfully measure intracellular changes in pH after endocytosis by macrophages. In addition to this, new Raman reporters in pH sensors have also been explored. Kong et al. [480] showed that by employing a novel Raman reporter, arene chromium tricarbonyl linked aminothiophenol (Cr(CO)_3_–ATP), functionalized on a gold-coated planar substrate, they could obtain a SERS spectrum in the mid IR region, between 1800 and 2200 cm^−1^, which avoided interference from the absorption of bio-molecules. This pH biosensor was extremely stable without nanoparticle aggregation and demonstrated strong sensitivity with clinical urine samples, thereby holding promise for pH sensing of body fluids for early disease diagnosis.

Redox potential changes in cells play an important role in understanding cellular functions, such as cell cycle, signaling pathways, and apoptosis [517,518]. Dysregulation of cell redox potential (measure of oxidative or reductive state in a living cell) is known to play a role in the pathology of diseases including cancer [519]. SERS substrates have been developed to quantitatively measure the intracellular redox potential of cells. El Said et al. [481] recently developed a single cell-based chip to look at the intracellular and extracellular redox potential of neural cells (PC12). They combined SERS with linear sweep voltammetry (LSV) to monitor biochemical changes in the cells. They immobilized single PC12 cells on a modified ITO substrate with a hexagonal array of gold nanodots deposited in a micrometer sized gap between two gold microelectrodes. They looked at the effects of applied redox potentials in a dopamine solution via SERS and LSV on cellular biochemical compositions and found difference in responses for cell lysates and living cells. In addition, they were able to look at the electrochemical activity of both bulk and single PC12 cells, thereby making this an effective substrate to analyze cellular processes. Substrates were also developed by the group [520] to monitor redox potential and biochemical changes in HepG2 cells when exposed to three different anticancer drugs.

**Figure 56:**
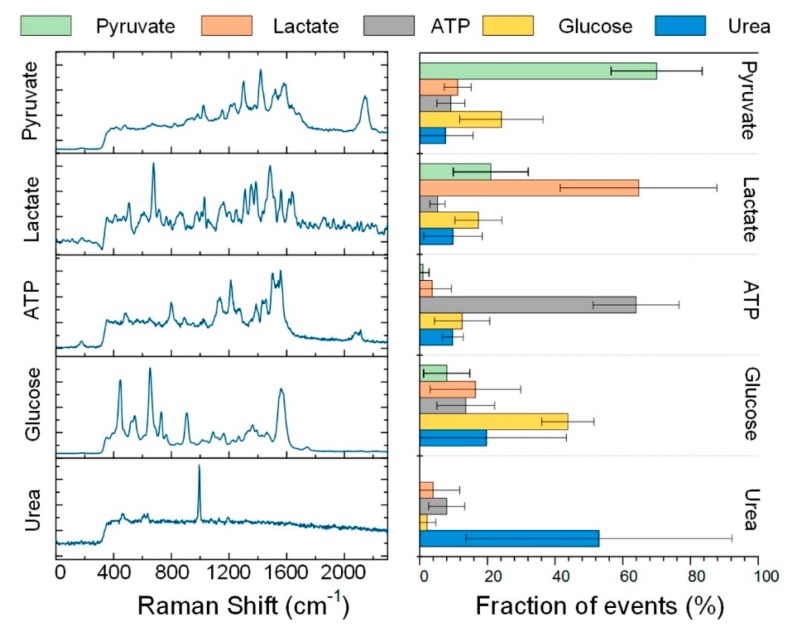
SERS spectra of pyruvate, L-lactate, adenosine triphosphate (ATP), D-glucose, and urea generated by principal component analysis (PCA) of a standard solution (left). These spectra were used to build SERS spectra database. Evaluation of the selectivity of the chemometric algorithm (right). SERS measurements were performed on five SERS nanosensors with a fresh standard solution of the target molecule. The error bars indicate standard deviation. Reproduced with permission from Lussier et al. [459]; further permissions related to the material excerpted should be directed to the ACS. Copyright (2016), the American Chemical Society.

Lussier et al. [459] developed a nanosensor to monitor metabolite secretion near living cells. They developed a nanosensor made of borosilicate nanopipettes decorated with gold nanoparticles with which they were able to simultaneously detect several metabolites, including pyruvate, lactate, ATP, and urea secreted near Madin–Darby canine kidney (MDCKII) epithelial cells (Figure 56). For the detection of the metabolites with low interference in a complex biological medium, they used a data-processing method that enabled sorting and counting with respect to a SERS spectra database. The nanosensor also enabled distance dependent sensing of the extracellular medium, thereby promising to be a useful tool for monitoring cell secretion events with both temporal and spatial resolution.

#### 6.2.3. Protein Detection

Proteins play a crucial role in many biological processes including catalyzing metabolic reactions and cell signaling, as well as immune response. The ability to detect and quantify specific protein markers is beneficial for diagnostics and understanding of disease progression. Examples of protein biomarkers indicative of health status include human chorionic gonadotropin (hCG) for pregnancy and prostate specific antigen (PSA) for prostate cancer, among others [515]. Immunoassays are the most commonly used technique to detect proteins that is based on the specificity of antigen–antibody interaction or protein–aptamer recognition. Several SERS based immunoassays have been developed in the recent years to detect proteins. Wang et al. [482] developed a simple SERS sandwich-like immunoassay to detect a pancreatic cancer biomarker, MUC4. They designed a nanochip consisting of gold nanoparticles functionalized with MUC4 antibody, which could extract and concentrate antigens from solution. SERS tags with a Raman reporter were then added on top, giving rise to intense SERS signals. This technique proved to be a simple diagnostic test for detecting MUC4 from clinical serum samples of patients. To further improve the detection sensitivity of MUC4, the surface of the nanochip was later modified [483] by using smooth mica. It was also found that by using a polymer or graphene monolayer, the stability and sensitivity of the assay could be improved.

Using the sandwich-like immunoassays, Song et al. developed several substrates to detect different biomarkers. They were able to detect carcino-embryonic antigen (CEA), which is a well-known lung cancer marker present in the blood of patients [521]. They were able to detect it between the ranges of 0.01 fg/mL and 1 ng/mL and achieved a limit of detection (LOD) of 0.01 fg/mL. They also detected human IgG concentrations between the ranges of 100 fg/mL and 100 ng/mL with an LOD of 2.5 fg/mL. In this assay, they used glass slides as substrates with silver nanorod arrays fabricated by oblique angle deposition. They then labeled the silver nanorods with a Raman reporter, 4-MBA and antibody [484]. Kong et al. [485] developed a similar approach using silica-encapsulated hollow gold nanospheres and a gold-patterned microarray substrate. They used this biosensor for the detection of VEGF (vascular endothelial growth factor), which is a protein marker for angiogenesis. Here, they used sensitive DNA aptamers that formed a G-quadruplex structure instead of VEGF antibodies to capture the target molecule. They demonstrated detection capabilities that were three to four orders of magnitude greater than that observed with conventional Enzyme-linked Immunosorbent Assay ELISA. They showed that implementing aptamers in the design of novel immunoassays holds great promise in sensitive biomarker detection.

Kavosi et al. [486] developed an immunosensor to detect PSA, a well-known prostate cancer biomarker. They used a multiwalled carbon nanotubes/ionic liquid–chitosan nanocomposite (MWCNTs/IL/Chit) as the substrate platform, which was decorated with gold nanoparticles containing polyamidoamine dendrimer (AuNPs-PAMAM). They achieved a detection limit of 0.5 ng/mL for PSA, with a concentration range up to 25 ng/mL. However, studies have shown that the levels of PSA change in prostate cancer patients during treatment with the progestin drug megestrol acetate, and range around 100 pg/mL [522]. In order to improve the sensitivity of detection of PSA, Yang et al. recently developed an aptamer-based sensor [487] using a core-satellite assembly of nanoparticles, with a magnetic nanoparticle core and gold nanospheres around it. The gold nanoparticles were functionalized with aptamers specific for detecting PSA. Using this aptasensor, they were able to detect PSA over a wide concentration range and achieved a limit of detection at 5 pg/mL. Yoon et al. [488] were further able to improve the limit of detection of PSA to 1 pg/mL using a sandwich-like immunoassay with gold nanospheres immobilized on a gold substrate. Capture of PSA was achieved via antigen-antibody interactions and sensitive SERS signals were obtained. This sensor also showed promising results in human serum samples, thereby providing opportunities for its usability in clinical diagnostics.

Multiplexed detection of proteins is essential in the development of SERS biosensors. Owens et al. [489] developed a sandwich-like assay consisting of a silicon substrate with Ag nanopillars that was decorated with antibodies specific for p53 and EGFR (Epithelial growth factor receptor), which are important cancer biomarkers. They used two Raman reporters, 4-aminothiophenol and 6-mercaptopurine, to investigate the effects of varying concentrations of the two target proteins on the SERS spectra. They observed that different protein and Raman reporter combinations displayed changes in the mechanical-stress responses exploited to carry out multiplexed detection. Xu et al. [490] were able to detect three disease biomarkers simultaneously using a SERS substrate. They employed self-assembled silver nanoparticle pyramids on a SERS active substrate and SERS tags to detect the biomarkers PSA, thrombin, and Mucin-1 simultaneously. They used DNA aptamers for target recognition and were able to achieve detection at attomolar concentration of proteins. With this biosensor, the LODs for the three biomarkers, PSA, thrombin, and Mucin-1 were 0.96, 85, and 9.2 aM, respectively.

Besides these conventional biosensors, applications combining SERS with other techniques to detect proteins have also been explored. Zhang et al. [491] developed an immunoassay with anti-gp41 antibody, a common HIV/AIDS diagnostic biomarker, by combining SERS and microfluidics with UV-vis spectroscopy read-out. They used a microfluidic chip encoded with 2D localized surface plasmon resonance (LSPR) structures of gold nanoparticles and modified by the gp41 antigen. Multiplexed detection of gp41 antibody at different concentrations was then carried out using this substrate. They were able to carry out this label-free assay within 30 min, from sample introduction to results, which is faster and more convenient than traditional ELISA with similar sensitivity of detection.

## 7. Conclusions and Outlook

This review has provided an overview on several key aspects of SERS. In the following, we attempt to provide some possible future developments that, in our opinion, are important for further elucidating the fundamental mechanisms underlying SERS and for its widespread application as a vibrational analytical tool.

The electromagnetic enhancement generated by metallic substrates is nowadays quite well understood on the basis of classical physics and allows one to predict and optimize the amplification as a function of the geometry of the sample [7]. When molecules are placed in very small gaps (less than 1 nm); however, quantum mechanical phenomena come into play and, only recently, they have started to be explored. For example, it has been demonstrated that the electromagnetic enhancement is limited by electron tunneling in gaps smaller than 1 nm [23,227,523]; moreover, theoretical treatments, based on the quantum mechanical nature of the vibrations and of the electromagnetic field in an optical cavity, revealed an unusual dependence of the anti-Stokes intensity on the excitation frequency [524]. The chemical enhancement has been historically subjected to different interpretations and more difficult to pin down compared to the electromagnetic one [9,10,134]. This has been probably due to the many intertwined factors that play a role, like the structure of the molecule, its adsorption geometry at the substrate surface, the detailed (nanometric) nature of the surface itself, and the excitation wavelength [9,10,11,25,133,135]. The study of the chemical enhancement has been assuming increasing relevance due to the variety of materials that are under investigation for the fabrication of SERS substrates (in addition to the traditional noble metals), like semiconductors and dielectrics. These materials offer a high degree of flexibility, since the interaction with the molecule can be optimized by varying the energy levels of the band edges and the width of band gap [24]. Graphene-enhanced Raman spectroscopy (GERS) seems also to be a promising field and further investigation of the graphene–molecule interaction is required [213,214,215]. Electromagnetic and chemical enhancement are typically described as different phenomena. The search for a unified theory of SERS is an open topic and a discussion of the current state of the art can be found in the paper by Ding et al. [7].

A widespread application of SERS as an effective analytical tool requires advances in the fabrication of SERS substrates and on the Raman instrumentation.

SERS substrates are a key element in a SERS experiment for several reasons. Fabricating a substrate with high enhancement and good uniformity/reproducibility is still a challenge and the use of scalable methods, suitable for mass production, is an important step towards the commercialization of substrates, which are currently limited [37,303]. Moreover, standardized substrates are crucial for the development of quantitative methods in SERS [48,49,50,51,52,53]. Lithographic/template methods tend to privilege the uniformity/reproducibility, while bottom-up assembly methods tend to produce smaller gaps and hence larger enhancements; specific strategies to overcome the drawbacks of the two methods, included their combination, need to be designed and implemented. The development of materials alternative to the traditional gold and silver is another important route not extensively explored yet. For example, metals with enhancing properties in the UV (i.e., aluminum, rhodium) could be useful for working with biomolecules, which typically exhibit small cross-sections [178,182,185,186,370]. A different approach is based on the use of dielectric/semiconductor materials that provide a moderate enhancement but, on the other hand, the weak absorption of light allows one to increase the signal by raising the laser power without damaging the sample [206,207]. The use of dielectrics/semiconductors is a field with a lot of room for experimental and theoretical work. For many applications, fluorescence is a major problem and the most efficient method to contrast it consists in working with excitations in the near infrared. Therefore, the development of substrates able to enhance Raman in that region is a relevant topic [21,525,526,527]. SERS as such possesses recognition capabilities; however, when the analyte is mixed in very complex matrices, it could be difficult to extract its signal due to the interference of other compounds. Therefore, the coupling with separation methods (i.e., microfluidics and chromatographic methods [427,428,429,430,431,432,433,434]) and the functionalization with capturing agents (antibodies [115,435,436,437,438,439], aptamers [115,440,441], self-assembled monolayers [442,443,444], and molecularly imprinted polymers [445,446,447,448,449]) are also crucial for a widespread use of SERS [35,47,425,426]. SERS substrates are typically disposable. The development of reversible SERS substrates, which can capture and release analytes in a controlled way, would be important when SERS is coupled to separation methods.

Instrumentation is also improving, making available more sensitive, compact, and cost-effective equipment. Portable or handheld instruments have become commercially available in the last decade and provide, as a main advantage, the possibility of carrying out on-site analysis [21,57,528]. It is worth mentioning that the so-called hyphenated instruments, that combine Raman with other analytical techniques, are also available (for example, Fourier transform infrared–Raman instruments [529]; other examples can be found in the website of Raman instrument manufacturers), and allow one to perform a more complete characterization of the sample. Tailoring the features of these instruments (weight, sensitivity, excitation wavelength, price, etc.) to the requirements of the specific application will undoubtedly broaden the range of applications of SERS.

## Figures and Tables

**Table 1:** Approximate maximum values for the electromagnetic enhancement ($G_{SERS}^{Em}$) and for the chemical enhancement ($G_{SERS}^{Chem}$). Reproduced with permission from Pilot et al. [81]. Copyright (2018), Springer International Publishing AG.

$G_{SERS}$	Approx. Max. Value	Note	Ref.
$G_{SERS}^{Em}$	$10^{8}$	Averaged over the substrate	[16,115]
$G_{SERS}^{Em}$	$10^{10}$	In a hot spot	[116]
$G_{SERS}^{Chem}$	$10^{2}$	Atomic scale roughness	[5,10,115,117]
$G_{SERS}^{Chem}$	$10^{4}$	Charge transfer resonance	[9]

**Table 2:** Plasma frequency ($\omega_{p}$) and onset of the interband transitions ($\omega_{Inter}$) for some common metals used in SERS. The former values are taken from West et al. [157]; the latter are taken from Cottancin et al. [160] (gold, silver and copper) and from Ehrenreich et al. [161] (aluminum).

Metal	$\omega_{p}$	$\omega_{Inter}$
**Gold**	8.9 eV (139 nm)	2 eV (620 nm)
**Silver**	9.2 eV (135 nm)	4 eV (310 nm)
**Aluminum**	12.7 eV (98 nm)	1.4 eV (886 nm)
**Copper**	8.7 eV (142 nm)	2 eV (620 nm)

**Table 3:** Summary of experimental studies investigating the distance dependence of the SERS enhancement/signal. Abbreviations. Ag-FON: Ag film over nanosphere; DNA: Deoxyribonucleic acid; PMMA: poly(methyl methacrylate).

Substrate	Spacer	Range [nm]	Probe	Ref.
Rough Ag film	Linear aliphatic thiols	0.8–2.5	–CH_3_ groups of aliphatic thiols	Compagnini et al. [237]
Rough Ag foil	Linear aliphatic thiols	1.6–2.8	*t-*butylbenzene	Kennedy et al. [238]
Ag islands	Langmuir-Blodgett films of arachidic acid	0.85–14	Phtalocyanine	Kovacs et al. [235]
Ag-FON	Al_2_O_3_	0–3	Trimethyl aluminum	Masango et al. [236]
Ag nanorods	DNA oligomers	1–6	Adenine	Marotta et al. [239]
Rough Ag film	PMMA	0–12	*p-*nitrobenzoic acid	Murray et al. [240]

**Table 4:** Selection of literature papers that provide a comparison between the local field and the far field dispersion in plasmonic systems. Papers indicated as “Experimental” can contain also plasmonic simulations, while papers indicated as “Simulations/Modelling” are purely theoretical.

System	Far Field Quantities	Reference
Solid substrates (Experimental)		
Au nanoparticles (NPs)	Extinction, scattering	D’Andrea et al. [256]
Single Au dimers and trimers	Scattering	Kleinman et al. [26]
Ag nanopillar array	Extinction	McFarland at al. [126]
Ag nanopillar array	Extinction	Michieli et al. [127]
Au nanocylinder array	Extinction	Guillot et al. [284]
Au elongated NP array	Extinction	Félidj et al. [285]
Au nanocylinder array	Extinction	Colas et al. [286]
Au nanopillar and nanotube array	Extinction	Doherty et al. [287]
Solution (Experimental)		
Ag spherical NPs	Extinction, scattering	Von Raben et al. [279]
	Extinction	Fornasiero et al. [280]
	Extinction	Kerker et al. [281]
	Extinction	Feilchenfeld et al. [282]
	Extinction	Le Ru et al. [283]
Silica (core)-Au (shell) NPs	Extinction, absorbance	Weber et al. [255]
Ag-Au nanocages	Extinction	Pilot et al. [225]
Ag nanowires	Extinction, absorbance	Becucci et al. [288]
Simulations/Modelling		
Ag, Au, Cu spherical NPs	Extinction, absorption, scattering	Messinger et al. [158]
Au nanospheres, Au-silica nanoshells, Au homo and hetero dimers	Extinction, scattering	Cacciola et al. [289]
Au spherical NPs	Extinction	Zuloaga et al. [290]
Ag spherical NPs and dimer	Extinction	Le Ru et al. [116]

**Table 5:** The symbols + and − indicate, as a rule of thumb, the spectral region in which the factors listed in the first column are more or less favored. Concerning the instrument sensitivity, the gratings and optics can be optimized for working in different spectral regions; the detectors can cover the visible region up to approximately 1100 nm (silicon-based CCDs) or regions further to the red (InGaAs based arrays). Considering that silicon detectors are more common and less expensive than their InGaAs counterparts, the “Instrument sensitivity” has been assigned as more favorable in the visible region.

	Visible	Near-Infrared
$G_{SERS}$	−	+
**Analyte cross-section**	+	−
**Instrument sensitivity**	+	−
**Reduced fluorescence interference**	−	+
**Transparency window**	−	+

**Table 6:** Desired features of SERS substrates, proposed by Natan [85] and Lin et al. [37].

Feature	Suggested Benchmark	Notes
High average enhancement	$>10^{5}$	Larger enhancements allow more sensitive and/or faster analysis.
Uniformity	Variations < 20%	Uniform and reproducible substrates make the work of the practitioner much easier, since one does not need to try several spots to find the most efficient one, and results are reproducible from substrate to substrate. Both these features are crucial if quantitative measurements are to be performed. Large areas are particularly useful with portable instruments, since they are normally not coupled to a microscope.
Reproducibility	Variations < 20%	
Large area	Some mm^2^	
Stability		Substrates should preserve a good performance for a sufficient time (say a month) after fabrication. Moreover, they should not be degraded by the solvents (or other agents) they get in touch with under working conditions.
Ease of fabrication/low cost of production		Low-cost and scalable fabrication methods for substrates are crucial for a widespread diffusion of the SERS technique.
Cleanliness of the surface		The surface of the substrate should not have residual contaminants from the fabrication process.

**Table 7:** Summary of the substrates and of the corresponding fabrication methods presented in this section.

Substrate	Synthesis/Fabrication	References
Aggregated NPs in solution (unstructured nanoparticles)	Wet chemistry (NP synthesis)	[39,40,308,309,310,311,312,313,314,315,316,317,318,319,320,321,322,323]
	Laser ablation (NP synthesis)	[324,325]
	Molecular linkers (aggregating method)	[326,327,328]
	Laser tweezers (aggregating method)	[329]
NPs assembled on a surface (structured nanoparticles)	Electrochemical roughening/deposition	[37,330]
	Deposition on functionalized surfaces	[42,331,332,333]
	Ink-jet printing	[334,335,336,337]
	Screen printing	[338,339]
	Pen on paper	[340]
	Electrospinning	[341,342,343]
	Laser direct writing	[344,345]
Ordered array of NPs (structured surfaces)	Anodic alumina template	[346,347,348,349,350,351]
	Electron beam lithography (EBL)	[43,44,45,352,353,354]
	Interference lithography	[355,356]
	Soft lithography	[46,357,358,359,360]
Commercial substrates		[361,362,363,364,365,366,367]

**Table 8:** Summary of the SERS applications in the biomedical field presented in this review (direct protocol).

Analyte	SERS Substrate	Ref.
MicroRNA and family members	Ag NR arrays	[450]
ssRNA bases: adenine cytosine	Ag NPs in microfluidic devices	[451]
RNAs: complimentary duplexes, short hairpin and small RNAs, and to diversify microRNA sequences	Positively charged spermine coated Ag NPs	[452]
ssDNA	Al nanocrystals	[184]
Cell structure distribution of phenylalanine and DNA	Au NPs, 60 nm diameter	[453]
Endosomal system of cultured eukaryotic cells	Au NPs, 30–50 nm diameter	[454]
Gastric cancer detection in blood plasma	Ag NPs, 34 nm diameter	[455]
Phenylalanine, tyrosine, tryptophan adenine, guanine (in DNA)	Ag NPs	[456,457]
Phenylalanine in human breast cancer cells	Au NPs	[456]
RNA biomarkers long amplicons RNA extracted from urine to detect prostate cancer	Ag NPs	[458]
Metabolite secretion from MDCKII cells	Borosilicate nanopipettes decorated with Au NPs	[459]
Cytochromes	Ag electrodes	[460]
Myoglobin	Immobilized Ag NPs, size 100 nm	[461]
Myoglobin and BSA	Ag NPs adsorbed on a nitrocellulose membrane	[462]
Heme-proteins	Ag NPs	[463]
Oligonucleotides	Ag NPs	[464]
Hen egg white lysozyme, avidin, cytochrome c, hemoglobin, BSA	Iodine-modified Ag colloids	[465]
*Escherichia coli*	Ag NPs	[466]
Respiratory human viruses	Ag NR arrays	[467]
*Escherichia coli O157, Salmonella typhimurium, and Staphylococcus aureus*	Ag nanocrystals (60–80 nm diameter) assembled on Ag NPs	[468]
Nine different *Escherichia coli* strains	Ag NPs in microfluidic devices	[63]
*Listeria monocytogenes* bacteria	Ag-Au bimetallic substrates	[469]

BSA: Bovine serum albumin; MDCKII: Madin–Darby canine kidney; NR: Nanorod; RNA: Ribonucleic acid; ss: Single stranded.

**Table 9:** Summary of the SERS applications in the biomedical field presented in this review (indirect protocol).

Analyte	Recognition Unit	SERS Substrate	Ref.
Viral DNA	DNA Hairpin	Au NPs	[470]
RSAD2 gene	DNA Hairpin	Nanowave Chip	[471]
microRNA	Molecular Beacon	Ag NPs	[472]
miR-21	ssDNA	Au NRs on Au substrate	[473]
ssDNA		AuNPs@SiNWAr	[474]
Modifications in ssDNA	DNA targeting BRCA1	Au-coated magnetic NPs on rGO on Au electrode	[475]
Bacterial DNA	Probe, target, and reporter DNA	Au NP-on-wire	[476]
CEA, AFP	Antibodies	Sandwich Au honeycomb array + Au nanostars	[477]
Pathogenic bacteria	Antibiotics	Nanoscopic Ag substrate + electrodeposited Ag-Au layer	[478]
Intracellular pH	Mercaptobenzoic acid (MBA)	Ag-MBA@SiO_2_	[479]
Intracellular pH	(Cr(CO)_3_–ATP)	Au-coated planar substrate	[480]
Intracellular and extracellular redox potential of neural cells	Dopamine	ITO electrode + hexagonally packed Au nanodots	[481]
Pancreatic cancer biomarker (MUC4)	MUC4 Antibody	Au NPs	[482,483]
CEA, human IgG	4-mercaptobenzoic acid (4-MBA) + antibody	Ag NR arrays on glass	[484]
VEGF	DNA Aptamer	Si-encapsulated hollow Au nanospheres and an Au-patterned microarray substrate	[485]
PSA	Antibody	MWCNTs/IL/chitosan + AuNPs-PAMAM	[486]
PSA	PSA Aptamer	Core-satellite magnetic NP (core)/Au NPs satellites	[487]
PSA	PSA Antibody	Sandwich Au Nanospheres on Au substrate	[488]
p53 and EGFR	P53 + EGFR antibodies	Si substrate + Ag nanopillars	[489]
PSA, thrombin, and Mucin-1	Aptamers	Self-assembled Ag NP pyramids on SERS substrate + SERS tags	[490]
Anti-gp41 antibody (HIV biomarker)	gp41	Microfluidic chip encoded with 2D LSPR structures of Au NPs	[491]

AFP: α-fetoprotein; AuNPs@SiNWAr: Silicon nanowire arrays decorated with Au nanoparticles; HIV: Human immunodeficiency viruses; CEA: carcinoembryonic antigen; EGFR: epithelial growth factor receptor; IgG: Immunoglobulin G; IL: Ionic liquid; ITO: Indium tin oxide; LSPR: Localized surface plasmon resonance; MWCNT: Multiwalled carbon nanotube; PAMAM: Poly(amidoamine); PSA: Prostate specific antigen; rGO: Reduced graphene oxide; VEGF: Vascular endothelial growth factor.

## Abbreviations

2B2MP	2-bromo-2-methylpropane
4-ABT	4-aminobenzenethiol
4-MBA	4-mercaptobenzoic acid
4-MBT	4-methylbenzenethiol
AFM	Atomic force microscopy
AFP	α-fetoprotein
Ag-FON	Silver film over nanospheres
AIDS	Acquired immune deficiency syndrome
APTMS	(3-Aminopropyl)trimethoxy silane
ATP	Adenosine triphosphate
AuNPs@SiNWAr	Silicon nanowire arrays decorated with Au nanoparticles
AZO	Aluminum-doped ZnO
BiASERS	Bi-analyte SERS
BPA	Bisphenol A
BPE	Trans-1,2-bis(4-pyridyl)-ethylene
BSA	Bovine serum albumin
BTA	Benzotriazole
BTZ	3-methoxy-4-(5′-azobenzo-triazolyl) phenylamine
CARS	Coherent anti-Stokes Raman scattering
CCD	Charged coupled device
cDNA	Complementary DNA
CEA	Carcino-embryonic antigen
CFU	Colony forming unit
Chit	Chitosan
CT	Charge transfer
CV	Cyclic voltammetry
CV^+^	Crystal Violet cation
DFT	Density functional theory
DMAB	4,4-dimercaptoazobenzene
DNA	Deoxyribonucleic acid
DOX	Doxorubicin
DPSD	Double step potential deposition
ds-DNA	Double stranded DNA
DTNB	5,5′-dithiobis(2-nitrobenzoic acid)
EBL	Electron beam lithography
ED	Electrochemical deposition
EDTA	Ethylenediaminetetraacetic acid
EGFR	Epithelial growth factor receptor
ELISA	Enzyme-linked immunosorbent assay
ER	Electrochemical roughening
FON	Film over nanosphere
GC	Gas chromatography
GERS	Graphene enhanced Raman scattering
GZO	Gallium-doped ZnO
hCG	Human chorionic gonadotropin
HIV	Human immunodeficiency viruses
HOMO	Highest occupied molecular orbital
HPLC	High pressure liquid chromatography
HRS	Hyper Raman scattering
IFI27	Interferon alpha-inducible protein 27
IFI44L	Interferon-induced protein 44-like
IgG	Immunoglobulin G
IL	Ionic liquid
InGaAs	Indium gallium arsenide
IR	Infrared
IRTP	Immortalized rat renal proximal tubule
IT	Indium tin oxide
LOD	Limit of detection
LSPR	Localized surface plasmon resonance
LSV	Linear sweep voltammetry
LUMO	Lowest occupied molecular orbital
MBA	Mercaptobenzoic acid
MDCKII	Madin–Darby canine kidney
MDR	Morphology dependent resonance
MIP	Molecularly imprinted polymer
miRNA	MicroRNA
MS	Molecular sentinel
MS-on-Chip	Molecular sentinel-on-chip
MWCNT	Multiwalled carbon nanotube
NIL	Nanoimprint lithography
NIR	Near infrared
NP	Nanoparticle
NR	Nanorod
PAMAM	Poly(amidoamine)
PAN	Polyacrylonitrile
PATP	p-aminothiophenol
PCB	Polychlorinated biphenyls
PCR	Polymerase chain reaction
PEG	Polyethylene glycol
PMMA	Poly(methyl methacrylate)
PSA	Prostate specific antigen
PVA	Poly(vinyl alcohol)
PVP	Polyvinylpyrrolidone
RDI	Relative diagnostic index
RIE	Reactive ion etching
RNA	Ribonucleic acid
ROS	Reactive oxygen species
SAM	Self-assembled monolayer
SCE	Saturated calomel electrode
SEM	Scanning electron microscopy
SERDS	Shifted excitation Raman difference spectroscopy
SERS	Surface-enhanced Raman scattering
SNR	Signal to noise ratio
SPR	Surface plasmon resonance
SRS	Stimulated Raman scattering
ssDNA	Single stranded DNA
SSE	Sequentially shifted excitation
SSRS	Subtracted shifted Raman spectroscopy
TAED	Template assisted electrochemical deposition
TCO	Transparent conductive oxide
TDVP	Temperature dependent vibrational pumping
TE	Transverse electric
TEM	Transmission electron microscopy
TERS	Tip enhanced Raman scattering
TLC	Thin layer chromatography
TM	Transverse magnetic
TNT	2,4,6-Trinitrotoluene
UPD	Underpotential deposition
UV	Ultraviolet
UV-Vis	UV Visible
VEGF	Vascular endothelial growth factor
WGM	Whispering gallery mode
